# Connexinplexity: the spatial and temporal expression of *connexin* genes during vertebrate organogenesis

**DOI:** 10.1093/g3journal/jkac062

**Published:** 2022-03-24

**Authors:** Rachel M Lukowicz-Bedford, Dylan R Farnsworth, Adam C Miller

**Affiliations:** Institute of Neuroscience, Department of Biology, University of Oregon, Eugene, OR 97403, USA

**Keywords:** Connexin, zebrafish, gap junction, single-cell RNA-seq

## Abstract

Animal development requires coordinated communication between cells. The Connexin family of proteins is a major contributor to intercellular communication in vertebrates by forming gap junction channels that facilitate the movement of ions, small molecules, and metabolites between cells. Additionally, individual hemichannels can provide a conduit to the extracellular space for paracrine and autocrine signaling. Connexin-mediated communication is widely used in epithelial, neural, and vascular development and homeostasis, and most tissues likely use this form of communication. In fact, Connexin disruptions are of major clinical significance contributing to disorders developing from all major germ layers. Despite the fact that Connexins serve as an essential mode of cellular communication, the temporal and cell-type-specific expression patterns of *connexin* genes remain unknown in vertebrates. A major challenge is the large and complex *connexin* gene family. To overcome this barrier, we determined the expression of all *connexins* in zebrafish using single-cell RNA-sequencing of entire animals across several stages of organogenesis. Our analysis of expression patterns has revealed that few *connexins* are broadly expressed, but rather, most are expressed in tissue- or cell-type-specific patterns. Additionally, most tissues possess a unique combinatorial signature of *connexin* expression with dynamic temporal changes across the organism, tissue, and cell. Our analysis has identified new patterns for well-known *connexins* and assigned spatial and temporal expression to genes with no-existing information. We provide a field guide relating zebrafish and human *connexin* genes as a critical step toward understanding how Connexins contribute to cellular communication and development throughout vertebrate organogenesis.

## Introduction

Animal development and homeostasis require coordinated cellular communication. One method of mediating communication is gap junction (GJ) channels. GJs are intercellular channels that provide a direct path of low resistance for ionic and small molecule exchange between cells (Evans and Martin 2009). These channels are formed by the coupling of 2 apposed hemichannels each contributed by adjacent communicating cells (Evans and Martin 2009; Orellana *et al.* 2013; Xing *et al.* 2019). Additionally, hemichannels can work independently within a single cell’s membrane, where they can release small molecules such as ATP and glutamate into the extracellular space for paracrine and autocrine signaling (Orellana *et al.* 2013; Xing *et al.* 2019). The proteins that create GJ channels are evolutionarily unrelated in vertebrates and invertebrates (Beyer and Berthoud 2018). Yet, despite little sequence similarity (Alexopoulos *et al.* 2004), the vertebrate Connexins and the invertebrate Innexin proteins have a similar structure, with both classes creating 4-pass, transmembrane-domain proteins that oligomerize to form each hemichannel within the plasma membrane (Beyer and Berthoud 2018). Moreover, the hemichannels and intercellular GJs created by Connexins and Innexins have similar structure and function (Beyer and Berthoud 2018). Outside of these traditional roles, Connexins can also modulate the formation of tunneling nanotubes that connect nonadjacent cells to facilitate longer distance communication (Soares *et al.* 2015; Okafo *et al.* 2017; Tishchenko *et al.* 2020). These varied functions in cellular communication are likely utilized individually and in combination in all animal tissues (Oyamada *et al.* 2005), yet are best studied in epithelial (Chanson *et al.* 2018), neural (Rozental 2000), and vascular (Figueroa and Duling 2009) systems. In these systems, mutations in human Connexin-encoding genes have been linked to defects in the development, regulation, and function including skin disorders (Richard *et al*. 1998, 2000, 2002; Richard 2005), cataracts (Willoughby *et al.* 2003; Wei *et al.* 2004), deafness (Kelsell *et al.* 1997; Xia *et al.* 1998; Grifa *et al.* 1999), cardiovascular disease (Jongsma and Wilders 2000; Yeh *et al.* 2001; Li *et al.* 2002), and gastrointestinal diseases (Temme *et al.* 1997; Maes *et al.* 2015a, 2015b). While Connexin channels serve as an essential form of cellular communication, the temporal and cell-type-specific expression patterns of *connexin* genes largely remain unknown.

A major challenge in characterizing *connexin* expression is the complexity of the gene family. In humans, there are 20 distinct *connexin* genes, and in other vertebrate lineages, the number of Connexin-encoding genes is similarly large and varies widely (Eastman *et al.* 2006; Cruciani and Mikalsen 2007; Mikalsen *et al.* 2020). Cell culture and in vitro work suggest that *connexin* complexity provides functional diversity governed by 4 general principles: first, hemichannels are created by hexamers of individual Connexin proteins (Tarzemany *et al.* 2017); second, single or multiple Connexin proteins can contribute to hemichannel formation (homo- or heteromeric hemichannels, respectively; Brink *et al.* 1997; He *et al.* 1999; Koval *et al.* 2014); third, GJs form intercellular channels via hemichannel docking at cell–cell junctions; fourth, each contributed hemichannel can contain the same or different Connexin proteins (homo- or heterotypic channels, respectively; Brink *et al.* 1997; He *et al.* 1999; Koval *et al.* 2014). The combinatorial possibilities of the gene family are restrained by molecular engagement rules that limit which Connexins are compatible to form mixed channels (Bruzzone *et al.* 1993; Elfgang *et al.* 1995; Koval 2006; Koval *et al.* 2014). These diverse possibilities culminate in each hemichannel having its own unique permeability properties, dependent upon the pore-lining amino acids and channel gating properties of the individual Connexins (Elfgang *et al.* 1995; Weber *et al.* 2004). These rules suggest that animals might take advantage of Connexin-based complexity in vivo to generate unique functional outcomes, but given the large number of genes, we know little about how vertebrates deploy this gene family.

Most of our knowledge of *connexin* expression in vivo comes from only a handful of well-characterized genes. These examples support the idea that *connexins* can be expressed in distinct tissues, such as in mouse where *gap junction a1*/Connexin 43 (*Gja1*/CX43) is expressed extensively in non-neuronal cells, including epithelia (Grueterich *et al.* 2002), heart (Beardslee *et al.* 1998; Li *et al.* 2002), and glia (Contreras *et al.* 2002). By contrast, *Gjd2*/CX36 is found almost exclusively in neurons (Srinivas *et al.* 1999). Within the same tissue, *connexin* expression can have distinct temporal patterns, such as *Gjb2/*CX26 and *Gjb1*/CX32 that are both found in the developing mouse neocortex at distinct developmental time points (Nadarajah *et al.* 1997). Within the group of well-studied Connexins, there are also a few enticing examples that suggest the rules of Connexin functional complexity found in vitro are relevant to in vivo function. For example, heteromeric channels formed by *Gjb1*/CX32 and *Gjb2/*CX26 are found in the mammary gland and the composition of channels changed during development (Locke *et al.* 2000). Heterotypic GJs composed of *gjd2a*/Cx35.5 and *gjd1a*/Cx34.1 are found at electrical synapses of zebrafish Mauthner cells where each Connexin was required for the localization of the other in the adjacent cell and both were necessary for synaptic transmission (Miller *et al.* 2017; Lasseigne *et al.* 2021). Finally, replacing the coding region of *Gja1*/CX43 with either *Gja5/*CX40 or *Gjb1*/CX32 results in sterility, cardiac malformations and arrhythmias, and mothers unable to nourish their pups, suggesting that each Connexin has unique properties that contribute to cellular homeostasis that are not interchangeable with other Connexins (Plum *et al.* 2000). While these examples provide a glimpse of functional complexity, understanding the expression of this gene family through vertebrate development remains the critical first step to decoding the complexity of *connexin* usage in vivo.

Here, we set out to examine the expression of all *connexins* in a vertebrate model system, the developing zebrafish, using single-cell RNA-sequencing (scRNA-seq) of cells derived from the entire animal during organogenesis [1–5 days postfertilization (dpf); Farnsworth *et al.* 2020]. Our analysis of *connexin* expression patterns revealed several trends, including that few *connexins* are broadly expressed, but rather, most *connexins* are spatially restricted to tissue- or cell-type-specific expression patterns. Most cells contain combinatorial signatures of *connexins* with unique profiles within distinct tissues. Finally, *connexin* expression is dynamic with temporal changes across the organism, tissue, and cell type. Our results reveal the complexity of spatiotemporal *connexin* control, highlighting novel aspects of well-studied *connexins* and revealing patterns for *connexin* genes with no prior expression information. We provide a field guide to relate zebrafish and human *connexins* genes, based on evolutionary homologies and expression similarities. Collectively, this represents an important step toward understanding *connexin* gene contributions in cellular communication throughout organogenesis and provides a foundation for comparative analysis in vertebrates.

## Materials and methods

### Single-cell RNA-sequencing

#### Embryo dissociation and cDNA library prep

As described by Farnsworth *et al.* (2020), larvae from the Tg(olig2: GFP)vu12 and Tg(elavl3: GCaMP6s) backgrounds were pooled (*n* = 15 per replicate), with 2 replicates at each sampled timepoint (1, 2, and 5 dpf). Cells from entire larvae were dissociated using standard protocols (Farnsworth *et al*. 2020). Dissociated cells were then run on a 10X Chromium platform using 10x v.2 chemistry aiming for 10,000 cells per run.

#### Alignment

To ensure that the full transcripts of the Connexin-encoding genes were represented in the dataset, we used gene models with lengthened 3′ UTRs across the zebrafish genome generated and validated by the Lawson Lab (Lawson *et al.* 2020). We ensured that the connexin genes were annotated properly by comparing pooled deep-sequencing information and extended the 3′ UTR regions as needed. Using this updated GTF file, we aligned reads to the zebrafish genome, GRCz11, using the 10X Cellranger pipeline (version 3.1). The updated GTF and other materials can be found at https://www.adammillerlab.com/.

##### Computational analysis

Cells were analyzed using the Seurat (V3.1.5) software package for R (V4.1.0) using standard quality control, normalization, and analysis steps. We performed principal component analysis (PCA) using 115 PCs based on a Jack Straw-determined significance of *P* < 0.01. Uniform manifold approximation and projection (UMAP) analysis was performed on the resulting 49,367 cells with 115 principal components (PC) dimensions and a resolution of 15.0, which produced 238 clusters. Code for this analysis and other materials can be found at https://www.adammillerlab.com/.

#### Cluster annotation

The unique barcode assigned to each cell was extracted from the original Farnsworth dataset (Farnsworth *et al.* 2020) and identified in our updated dataset. For each updated cluster, we analyzed the percentage of cells contributing which were associated with the original Farnsworth’s clusters. Frequently, we found the updated dataset contained clusters with a significant proportion of cells (>80%) from a single Farnsworth cluster, and in such, we transferred the annotation from the original cluster to the updated cluster. We also found instances of a single Farnsworth cluster breaking nearly evenly across 2 of the updated clusters—for example, the original dataset had a single “photoreceptor” cluster (cluster 115), whereas the updated data had 2 clusters (clusters 13 and 14) with cells from original photoreceptor cluster. Further analysis revealed that these 2 new clusters represented likely rods and cones. Finally, we also found updated clusters that did not have a clear previous annotation. In these instances, we analyzed the most differentially expressed genes from that cluster and compared them with canonical markers.

### Fluorescent RNA in situ

Custom RNAscope probes to target connexin genes were designed and ordered through ACD (https://acdbio.com/; for probes, please see reagent table in Supplementary Table 5). For the fluorescent in situs, we used a modified RNAscope protocol (Gross-Thebing *et al.* 2014). Briefly, 1 dpf embryos were fixed for 2 h at room temperature in 4% paraformaldehyde (PFA) and then stored in 100% methanol at −20°C. The tissue was then exposed to protease plus for 30 min, washed with PBS with 1% Triton X (PBSTx), and then hybridized with the 1× probe overnight at 40°C. Standard RNAscope V2 multiplex reagents and Opal fluorophores were used, with the modification that PBSTx that was used for all wash steps. Stained tissue was either mounted (whole mount) or immediately cryo-sectioned and mounted with ProLong Gold Antifade (ThermoFisher). Full protocol can be found at doi.org/10.17504/protocols.io.b47vqzn6.

### Zebrafish husbandry

Fish were maintained by the University of Oregon Zebrafish Facility using standard husbandry techniques (Westerfield 2000). Embryos were collected from natural matings, staged, and pooled. Animals used in the original Farnsworth data were: Tg(olig2: GFP)vu12 and Tg(elavl3: GCaMP6s) (Farnsworth *et al.* 2020), and animals used for RNAscope in situs were ABC-WT. Animal use protocol AUP-18-35 was approved by the University of Oregon IACUC committee and animal work was overseen by Dr. Kathy Snell.

## Results

### Zebrafish have 41 *connexin* genes

To understand *connexin* expression throughout organogenesis, we first set out to ensure the entire *connexin* gene family in zebrafish was identified. Previous efforts (Eastman *et al.* 2006; Watanabe 2017) and a recent phylogenetic approach to identify the full teleost *connexin* family (Mikalsen *et al.* 2020) captured 40 individual *connexin* genes. Through reciprocal BLAST analysis between the zebrafish genome and (1) human and (2) other teleost Connexin sequences, coupled with phylogenetic analysis, we identified the 40 previously noted *connexins* and one previously unreported *connexin*, *gjz1*, which is conserved in mammals but forms an outgroup with the rest of the Connexin proteins (Supplementary Figs. 1 and 2).

Across the family of *connexin* genes, there are 7 human *connexins* for which zebrafish only has a single homolog, 8 human *connexins* for which zebrafish has 2 homologs, 2 zebrafish *connexins* that have no direct homolog but share sequence similarity to human *connexins*, and 16 zebrafish *connexins* that are not present in humans but are conserved in other teleost and mammalian lineages (Mikalsen *et al.* 2020). We summarize these relationships in Table 1, listing zebrafish *connexin* genes and their closest relationship with their human counterparts, providing known human and zebrafish expression patterns and phenotypes for comparison. For clarity, we denote *connexins* by their Greek name and by their predicted molecular weight, a naming structure consistent with HUGO (Bruford *et al.* 2020) and ZFIN standards (Sprague *et al.* 2001; Table 1, Supplementary Table 1). The table is organized to emphasize Connexin similarities based on evolutionary homology, protein sequence, and expression, in alphabetical order of zebrafish *connexin* genes and denotes human similarity across merged rows. There are a limited number of rows where the zebrafish *connexin* gene resembles its human counterpart(s), but the genes are not direct homologs. For example, the human *GJB2/GJB6* genes are duplicated in the human lineage while having only a single similar gene in zebrafish called *gjb8* (Mikalsen *et al.* 2020). Despite not being direct homologs, expression and mutant analyses have found that zebrafish *gjb8* and human *GJB2/GJB6* genes are all involved in inner-ear support cell function and loss of these genes in their respective systems causes deafness (Grifa *et al.* 1999; Snoeckx *et al.* 2005; Chang-Chien *et al.* 2014). The comprehensive list of 41 zebrafish *connexin* genes provided a basis to examine the expression patterns of this gene family.

**Table 1.** A field guide to zebrafish *connexins*.

Zebrafish Connexin gene/protein	Phenotypes associated with zebrafish Connexin gene/protein	Updated scRNA-seq tissue/cluster	Human Connexin gene/protein	Diseases associated with human Connexin gene/protein
*gja1a/*Cx40.8	*No known phenotype*	Neural crest, connective tissue, and nervous system	GJA1/CX43	Bone, skin, eye, teeth, heart, and digit abnormalities (Dasgupta *et al.* 2001; Paznekas *et al.* 2003; Paznekas *et al.* 2009; Brice *et al.* 2013; Hu *et al.* 2013)
*gja1b/*Cx43	Smaller body shape and shortened fins, shorter vertebrae, disrupted regeneration, and diminished motile cilia (Haffter *et al*. 1996; Henke *et al*. 2017; Misu *et al*. 2016; Hoptak-solga *et al*. 2008; Zhang *et al*. 2020)	Broadly expressed		
*gja2/*Cx39.9	Decreased skeletal slow muscle contractability (Hirata *et al.* 2012)	Skeletal muscle	—	—
*gja3/*Cx46	Heart abnormalities (Chi *et al.* 2008, 2010)	Lens, heart	GJA3/CX46	Cataracts (Mackay *et al.* 1999; Burdon *et al.* 2004; Yang *et al.* 2010; Yao *et al.* 2011)
*gja4/*Cx39.4	Disrupted pigment patterns (Watanabe *et al.* 2016)	Endothelial and pigment cells	GJA4/CX37	Cardiovascular abnormalities (Yeh *et al.* 2001)
*gja5a/*Cx45.6	Faster vessel growth (Denis *et al.* 2019)	Muscle and endothelial	GJA5/CX40	Cardiovascular abnormalities (Groenewegen *et al.* 2003; Makita *et al.* 2005; Gollob *et al.* 2006; Yang *et al.* 2010; Wirka *et al.* 2011)
*gja5b/*Cx41.8	Leopard pigment patterns and faster vessel growth (Frohnhöfer *et al*. 2016; Watanabe *et al*. 2016; Henke *et al*. 2017; Watanabe 2017; Denis *et al*. 2019)	Pigment cells and endothelial		
—	—	—	GJA6P/CX43px	—
*gja8a/*Cx79.8	*No known phenotype*	Lens	GJA8/CX50	Cataracts (Berry *et al.* 1999; Polyakov *et al.* 2001; Willoughby *et al.* 2003; Hansen *et al.* 2007)
*gja8b/*Cx44.1	Cataracts (Ping *et al.* 2021)	Lens		
*gja9a/*Cx55.5	Disrupted perception of light stimulation (Klaassen *et al.* 2011)	Nervous system and integument	GJA9/CX58	*No known implications*
*gja9b/*Cx52.9	*No known phenotype*	Retina		
*gja10a/*Cx52.7	*No known phenotype*	Low expression in this dataset	GJA10/CX62	*No known implications*
*gja10b/*Cx52.6	*No known phenotype*	Retina		
*gja11/*Cx34.5	*No known phenotype*	Low expression in this dataset	—	—
*gja12.1/*Cx28.9	*No known phenotype*	Liver, intestine, and kidney	—	—
*gja12.2/*Cx28.1	*No known phenotype*	Intestine	—	—
*gja13.1/*Cx32.3	*No known phenotype*	Liver, intestine, and kidney	—	—
*gja13.2/*Cx32.2	*No known phenotype*	Macrophage	—	—
*gjb1a/*Cx27.5	*No known phenotype*	Schwann cell	GJB1/CX32	Neuropathy (*X-linked Charcot-Marie-Tooth*; Bergoffen *et al.* 1993; Ionasescu *et al.* 1996)
*gjb1b/*Cx31.7	Disrupted spacing of Muller glia cells (Charlton-Perkins *et al.* 2019)	Schwann cell		
*gjb3/*Cx35.4	*No known phenotype*	Integument	GJB3/CX31	Deafness and skin abnormalities (Xia *et al*. 1998; López-Bigas *et al*. 2001; Richard *et al*. 1998, 2000; Richard 2005)
*gjb7/C*x28.8	*No known phenotype*	Integument, hair cell, and olfactory neurons	GJB7/CX25	*No known implications*
*gjb8/*Cx30.3	Disrupted inner-ear development (Chang-Chien *et al.* 2014)	Integument, pigment cell, endothelial, and hair cell	GJB2/CX26	Deafness and skin abnormalities (Kelsell *et al*. 1997; Willems 2000; Richard *et al*. 2002; Richard 2005; Iossa *et al*. 2011)
			GJB6/CX30	Deafness and skin abnormalities (Grifa *et al*. 1999; del Castillo *et al*. 2002; Lamartine *et al*. 2000; Richard 2005)
*gjb9a/*Cx28.6	*No known phenotype*	Integument	—	—
*gjb9b/*Cx30.9	*No known phenotype*	Integument, macrophage		
*gjb10/*Cx34.4	Impaired cardiac function (Okamoto *et al.* 2020)	Integument, neural crest, and nervous system	GJB4/CX30.3	Skin abnormalities (Macari *et al.* 2000; Richard 2005)
			GJB5/CX31.1	*No known implications*
*gjc1/*Cx52.8	*No known phenotype*	Muscle, neural crest, and nervous system	GJC1/CX45	*No known implications*
*gjc2/*Cx47.1	*No known phenotype*	Schwann cell	GJC2/CX47	Myelin disorders and lymphatic abnormalities (Uhlenberg *et al*. 2004; Orthmann-Murphy *et al*. 2007; Ferrell *et al*. 2010)
—	—	—	GJC3/CX29	*No known implications*
*gjc4a.1/*Cx44.2	*No known phenotype*	Vasculature and integument	—	—
*gjc4a.2/*Cx44.5	*No known phenotype*	Integument		
*gjc4b/*Cx43.4	Disrupted left/right symmetry and abnormal Kupffer's vesicle development (Hatler *et al.* 2009)	Broadly expressed		
*gjd1a/*Cx34.1	Loss of electrical synapses and disrupted startle response (Miller *et al.* 2017)	Nervous system and retina	GJD2/CX36	Epilepsy associated (Wang *et al.* 2017)
*gjd1b/*Cx34.7		Nervous system, retina, and muscle		
*gjd2a/*Cx35.5	Myopia, loss of electrical synapses, and disrupted startle response (Miller *et al.* 2017; Quint *et al.* 2021)	Nervous system and retina		
*gjd2b/*Cx35.1	Myopia (Quint *et al.* 2021)	Nervous system and retina		
*—*	*—*	*—*	GJD3/CX31.9	*No known implications*
*gjd4/*Cx46.8	*No known phenotype*	Skeletal muscle	GJD4/CX40.1	*No known implications*
*gjd5/*Cx40.5	*No known phenotype*	Low expression in this dataset	*—*	*—*
*gjd6/*Cx36.7	Abnormal cardiac muscle tissue development (Sultana *et al.* 2008)	Heart	*—*	*—*
*gje1a/Cx23.9*	*No known phenotype*	Lens	GJE1/CX23	*No known implications*
*gje1b/Cx20.3*	*No known phenotype*	Skeletal muscle and nervous system		
*gjz1/Cx26.3*	*No known phenotype*	Nervous system	*—*	*—*

### The *connexin* gene family is broadly expressed, but spatially distinct

**Fig. 1.**
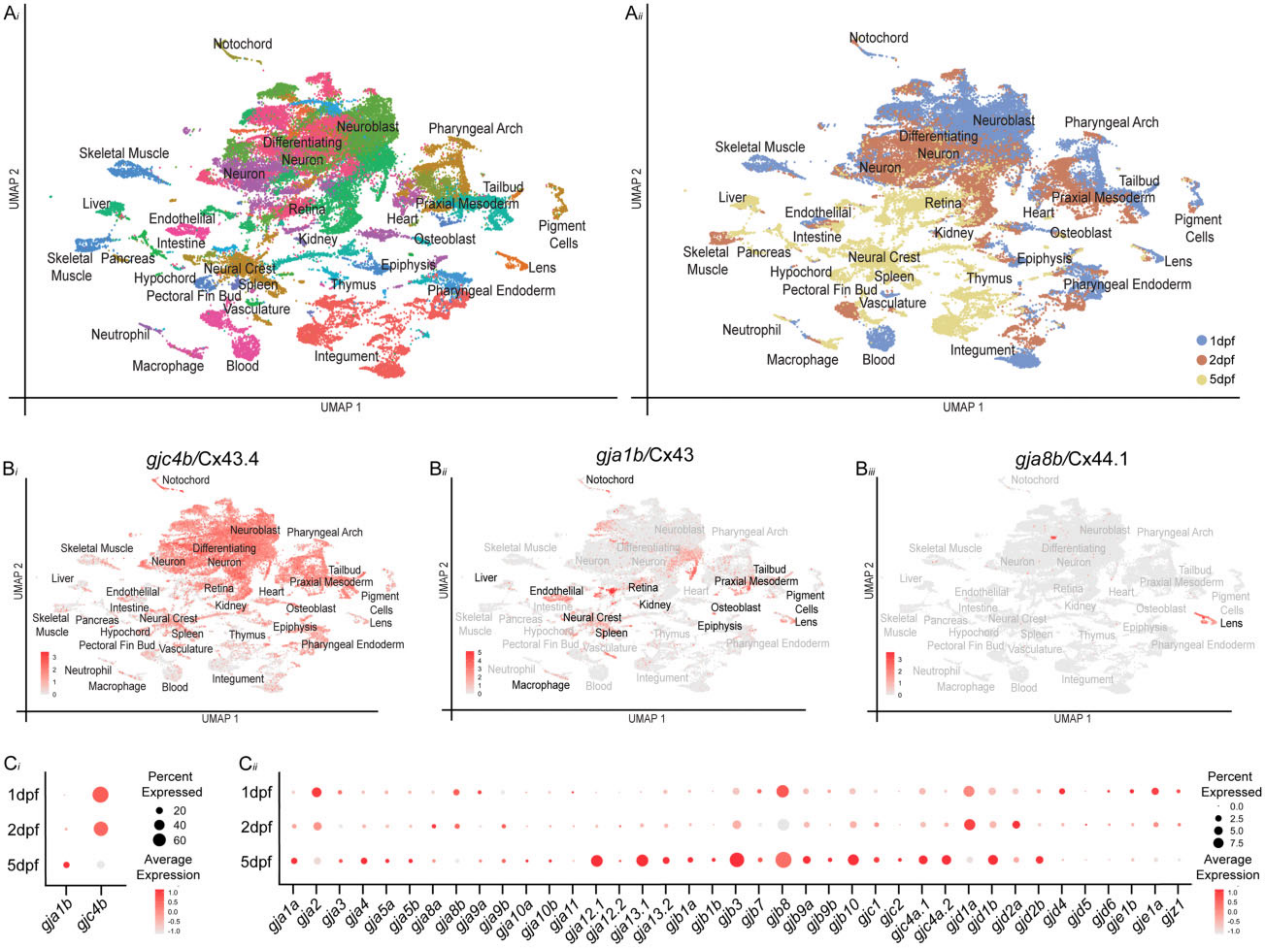
scRNA-seq dataset of zebrafish organogenesis and *connexin* expression. a*i*) Clustered cell types, where each dot represents a single cell and each color represents a set of transcriptionally related cells. a*ii*) The age of animals from which cells were dissociated denoted by color—1 dpf cells are blue, 2 dpf cells are orange, and 5 dpf cells yellow. b*i*–b*iii*) Expression of well-studied *connexins* in the dataset, where gray represents low expression and red represents the highest level of expression. b*i*) *gjc4b*/Cx43.4 is expressed broadly across the dataset. b*ii*) *gja1b/*Cx43 is expressed in a large number of clusters, with notable patterns in liver, endothelial, macrophage, neural crest, spleen, retina, kidney, epiphysis, osteoblast, mesoderm, tailbud, pigment cells and lens clusters. b*iii*) *gja8b*/Cx44.1 is expressed in lens clusters. c*i*) Broadly expressed *connexins*, *gja1b*/Cx43 and *gjc4b*/Cx43.4 and (c*ii*) the remaining *connexin* family shown for each sampled time point. Here, all cells from the corresponding age are pooled and the percent of cells expressing a given *connexin* are represented through dot size while the relative expression level is denoted through color intensity.

Next, we examined the spatiotemporal expression patterns of the zebrafish *connexin* genes through organogenesis using scRNA-seq. We used our recent scRNA-seq atlas dataset in which cells were dissociated from whole embryos at 1, 2, and 5 dpf, and resultant single-cell expression profiles were captured using the 10X platform (Farnsworth *et al.* 2020). In our initial analysis of the data, we found that many of the *connexin* genes lacked expression information. An examination of the *connexin* gene models generated by Ensembl (GRCz11_93) that were used for mapping single-cell reads revealed that most annotations were truncated at or near the end of the protein-coding sequence, with most lacking 3′UTRs leading to a failure in capturing the 3′-biased 10X sequencing information (Supplementary Fig. 3). To amend this, we used a recently updated gene annotation file that extends gene models (Lawson *et al.* 2020), evaluated and updated each *connexin* gene model in reference to bulk RNA-seq data (Miller *et al*. 2013), and imported the Greek gene names. Using this updated gene annotation file, we processed the scRNA-seq data using Cellranger (Zheng *et al.* 2017) and evaluated clustering and transcriptional profiles with Seurat (Satija *et al.* 2015). Analysis of the updated scRNA-seq dataset captures transcriptional profiles that appear to represent all major tissues of the developing zebrafish (Fig. 1, a*i* and a*ii*) and contains 49,367 cells and 238 clusters. This is 5,355 more cells and 18 more clusters than the original analysis (Farnsworth *et al.* 2020), as expected due to the richer transcriptional information captured from the updated gene model (Lawson *et al.* 2020). In our original analysis, we extensively annotated each cluster, assigning the most likely anatomical annotation based on comparing the differentially expressed genes for each cluster to RNA in situ patterns (Farnsworth *et al.* 2020). We transferred these previous annotations to our updated analysis by identifying cell-specific barcodes from the original dataset, identifying them in the updated clusters, and transferring the cluster annotations (Fig. 1, a*i* and a*ii*; Supplementary Tables 1 and 2). As a result, we identified all 220 original clusters (Farnsworth *et al.* 2020) and annotated the remaining clusters by analyzing RNA in situ expression information for the most differentially expressed genes (Supplementary Table 3). The updated scRNA-seq dataset greatly improves the capture of *connexin* expression throughout the atlas (Supplementary Fig. 3), allowing us to examine their spatiotemporal expression pattern during zebrafish organogenesis.

Using the updated scRNA-seq organogenesis dataset, we examined the expression of each *connexin* related to its clusters, its correlation with marker gene expression, and with cluster annotations (Supplementary Fig. 4, a–oo, Supplementary Table 4). Overall, *connexin* genes had a variety of expression patterns, varying from nearly ubiquitous to cluster-specific and showing a variety of temporal profiles, including constant expression over time or temporal specificity (Fig. 1, b and c, Supplementary Fig. 4, a–oo). To begin to evaluate the dataset’s utility, we first turned our attention to several well-studied *connexin* genes. First, *gjc4b*/Cx43.4 displayed the broadest expression, with particularly high levels in the nervous system, and with diminishing expression from 1 to 5 dpf (Fig. 1, b*i* and c*i*; Supplementary Fig. 5; Supplementary Table 4). This is similar to expression reports for *gjc4b*/Cx43.4 that used RNA in situ and transgenic methods (Thisse *et al.* 2001; Baxendale *et al.* 2012; Wierson *et al.* 2020). *gja1b*/Cx43 is another well-described *connexin*, with broad expression in the cardiovascular system, non-neuronal cells of the retina and central nervous system, mesenchymal cells such as chondrocytes, and within the digestive system including the pancreas (Thisse and Thisse 2004; Chatterjee *et al.* 2005; Iovine *et al.* 2005; Hoptak-solga *et al.* 2008; Yang *et al.* 2020). We find that the expression of *gja1b/*Cx43 within the updated clusters largely matches these reported expression patterns (Fig.1 b*ii*; Supplementary Fig. 4b; Supplementary Table 4). We also find expected patterns for *connexins* that have well-known, spatially restricted expression. For example, *gja8b*/Cx44.1 is expressed almost exclusively in the early developing lens(Cason *et al.* 2001; Thisse and Thisse 2005; Yoshikawa *et al.* 2017; Farnsworth *et al.* 2021), and in the scRNA-seq dataset, we find expression of *gja8b*/Cx44.1 within clusters with transcriptional profiles consistent with lens cells (Fig. 1b*iii*; Supplementary Fig. 5; Supplementary Table 4). Furthermore, we find *gja2*/Cx39.9 expression in presumptive skeletal muscle cells, *gjd6/*Cx36.7 specifically in presumptive cardiac muscle, and both *gja9b*/Cx52.9 and *gja10b*/Cx52.6 in presumptive horizontal cells, all well-matching published reports on the expression of these genes (Sultana *et al.* 2008; Hirata *et al.* 2012; Yoshikawa *et al.* 2017; Greb *et al.* 2018; Farnsworth *et al.* 2021; Supplementary Fig. 5; Supplementary Table 4). Taken together, we conclude that the data represented in the updated dataset provide a useful resource for determining the spatiotemporal patterns of *connexin* expression during zebrafish organogenesis.

### *Connexins* exhibit complex and combinatorial patterns of expression

**Fig. 2.**
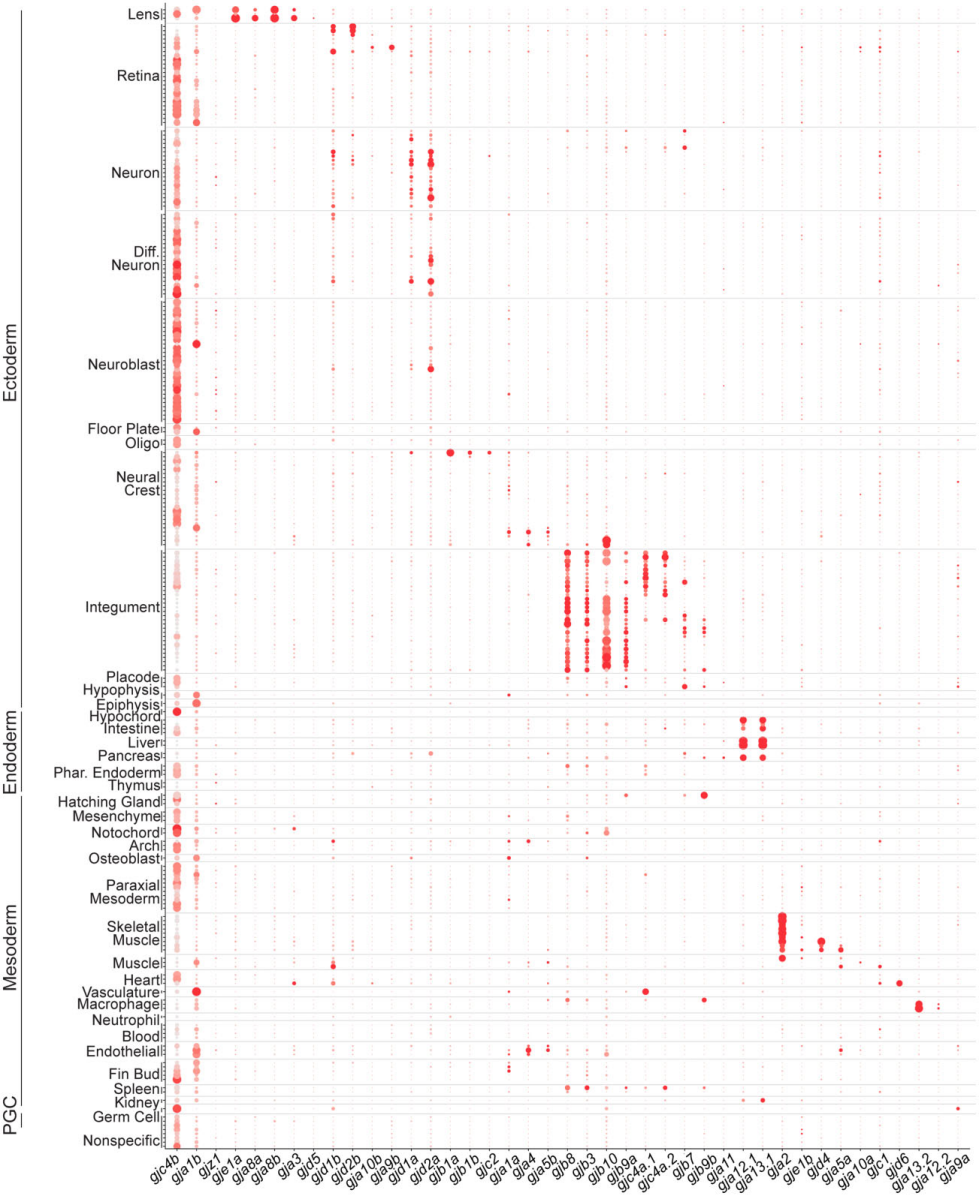
Connexin expression during zebrafish organogenesis. Clusters are organized by annotations and grouped into tissues and germ layers denoted on the *y*-axis. Along the *x*-axis, *connexins* are arranged based on spatial expression patterns. Each dot represents a single cluster. The percent of cells expressing a given *connexin* are represented through dot size while the relative expression level is denoted through color intensity. Diff. Neuron, differentiating neuron; Oligo, oligodendrocyte; Phar. Endoderm, pharyngeal endoderm; Arch, pharyngeal arch; PGC, primordial germ cell.

To examine the relationship of *connexin* gene expression relative to one another, we organized the scRNA-seq clusters by their tissue annotations and plotted both expression levels and percentage of cells within each cluster (Fig. 2). When arranged in this fashion, the complexity of *connexin* expression within putative tissues and cell types is revealed. In particular, unique combinatorial patterns of *connexins* are observed within tissues developing from all germ layers. For example, within neural clusters (ectoderm), we find that there are 4 broadly expressed *connexins*, yet each displays bias to either the retina, *gjd1b/*Cx34.7 and *gjd2b/*Cx35.1, or central nervous system, *gjd1a/*Cx34.1 and *gjd2a/*Cx35.5 (Fig. 2; Supplementary Fig. 4, af–ai; Supplementary Table 4). Within the skeletal muscle clusters (mesoderm), a unique set of *connexins* are expressed and display a nested hierarchy of expression, with *gja2/*Cx39.9 in all skeletal muscle clusters, *gja5a*/Cx45.6 and *gjd4*/Cx46.8 restricted to slow muscle clusters, and *gje1b*/Cx20.3 restricted to fast muscle clusters (Fig. 2; Supplementary Fig. 4, c, jj, f, and nn). We also observed temporally complex patterns of expression. For example, within presumptive intestinal epithelial cells (endoderm), we find that *gjc4b*/Cx43.4 expression diminishes from 1 to 5 dpf, while *gja13.1*/Cx32.3 begins expression at 2 dpf and continues at 5 dpf and *gja12.1*/Cx28.9 becomes coexpressed at 5 dpf (Fig. 2; Supplementary Figs. 4, q and o and 6). Finally, we observed that primordial germ cells (PGCs) express several different *connexins*, including *gja9a*/Cx55.5, *gjb8*/Cx30.3, *gjc4b*/Cx43.4, and *gjd1b*/Cx34.7 (Fig. 2; Supplementary Figs. 4, w, j, gg, and ee and 7). These observations highlight aspects of the complexity of *connexin* spatial and temporal expression patterns within and across tissues and cell types during zebrafish organogenesis.

### Cell-type-specific expression of *connexins* in the integument in vivo

**Fig. 3.**
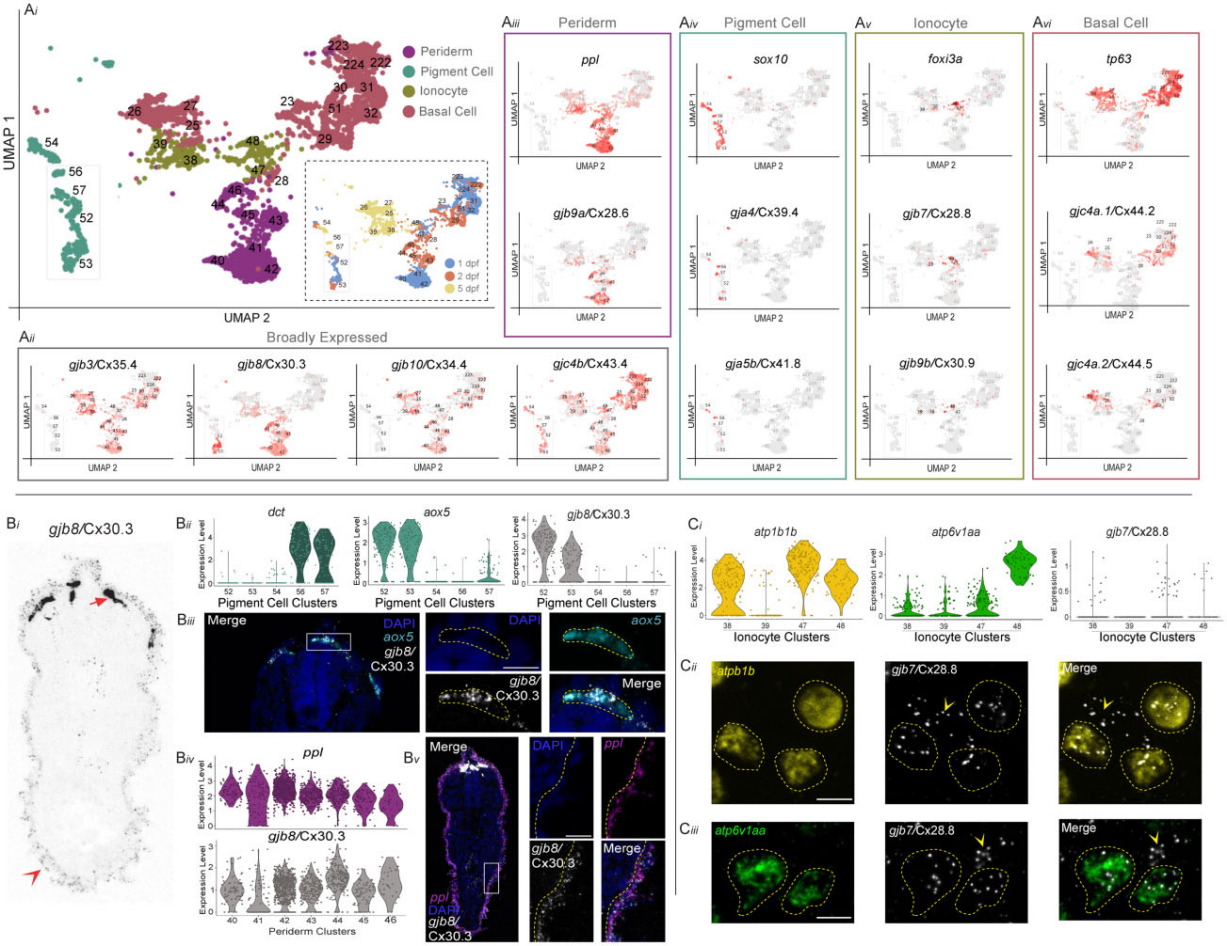
Connexin expression in the zebrafish integument during organogenesis. a*i*) The developing integument includes periderm, pigment cells, ionocytes, and basal cells. Relevant integument clusters were subsetted from the scRNA-seq dataset. Inset shows the age of animals from which cells were dissociated. a*ii*) Four *connexins* are broadly expressed in integument clusters, *gjb3/*Cx35.4*, gjb8/*Cx30.3*, gjb10/*Cx34.4, and *gjc4b/*Cx43.4. Gray represents low expression and red represents the highest level of expression. a*iii*) Periderm marker *ppl* and *gjb9a/*Cx28.6 are expressed in clusters 40–46. a*iv*) Neural crest-derived pigment cell marker *sox10* and *gja4/*Cx39.4 are expressed in clusters 52–57, while *gja5b/*Cx41.8 is only expressed in clusters 54 and 56. a*v*) Ionocyte marker *foxi3a* and *gjb7/*Cx28.8 are expressed in clusters 38, 39, 47, and 48. a*vi*) Basal cell marker *tp63* and *gjc4a.1/*Cx44.2 are expressed in clusters 23, 25–32, 51, 222–224, while *gjc4a.2/*Cx44.5 is only expressed in clusters 25–29. b*i*) Fluorescent RNA in situ for *gjb8/*Cx30.3 in a transverse cross-section of a 1 dpf zebrafish embryo, contrast is inverted for clarity. Dorsal is up, section is from the trunk. Strong expression of *gjb8/*Cx30.3 in neural crest cells is denoted with arrow and weaker, but distinct, periderm expression is denoted with arrowhead. b*ii*) Within the pigment cell clusters the melanocyte marker *dct* is expressed in clusters 56 and 57, whereas xanthophore marker *aox5* is primarily expressed in clusters 52 and 53. *gjb8/*Cx30.3 is predominantly expressed in clusters 52 and 53. b*iii*) Transverse cross-section of a 1 dpf zebrafish embryo stained with DAPI (blue) and fluorescent RNA in situ against *aox5* (cyan) and *gjb8/*Cx30.3 (white), with white box denoting the zoomed panels at the right. Scale bar = 10 µM. b*iv*) Expression of *ppl* and *gjb8/*Cx30.3 within the periderm clusters. b*v*) Transverse cross-section of a 1 dpf zebrafish embryo stained with DAPI (blue) and fluorescent RNA in situ against *ppl* (purple) and *gjb8/*Cx30.3 (white) with white box denoting the zoomed panels at the right. c*i*) Within the ionocyte clusters the Na+,K+-ATPase-rich cell and H+-ATPase-rich cell markers *atp1b1b* and *atp6v1aa*, respectively, are expressed in conjunction with low expression of *gjb7/*Cx28.8. c*ii*) Fluorescent RNA in situ in a 1 dpf zebrafish embryo against *atp1b1b* (yellow), *gjb7/*Cx28.8 (white), with merged signal (right). *atp1b1b* expressing cells are outlined with a dashed yellow line, and *gjb7/*Cx28.8 signal outside of those cells are marked with yellow arrowhead. Scale bar = 10 µM. c*iii*) Fluorescent RNA in situ in a 1 dpf zebrafish embryo against *atp6v1aa* (green), *gjb7/*Cx28.8 (white), with merged signal (right). *atp6v1aa* expressing cells are outlined with a dashed yellow line, and *gjb7*/Cx28.8 signal outside of those cells are marked with yellow arrowhead. Scale bar = 10 µM.

To validate that the *connexin* expression identified in the updated atlas related to in vivo tissues and cell types, we examined the integument, or the embryonic skin, as it represented one of the most striking trends of combinatorial expression (Fig. 2). Throughout zebrafish organogenesis, the integument is composed of distinct cellular populations including the periderm (the outermost epidermal layer), the basal cells (a keratinocyte stem cell population), the ionocytes (epithelial cells that maintain osmotic homeostasis), and the pigment cells (neural crest-derived cells that provide pigmentation; Guellec *et al.* 2004; Eisenhoffer *et al.* 2017). These individual cell populations are molecularly identifiable using distinct markers including *ppl* (periderm; Thisse *et al.* 2001; Thisse and Thisse 2004), *tp63* (basal cells; Lee and Kimelman 2002), *foxi3a* (ionocytes; Jänicke *et al.* 2007), and *sox10* (pigment cells; Budi *et al.* 2008; Eisenhoffer *et al.* 2017; Fig. 3; Supplementary Fig. 8). We used these canonical markers in conjunction with our annotations (Supplementary Fig. 8; Supplementary Table 2) to identify clusters that represent all 4 cell types of the integument (Fig. 3a*i*). We identified all *connexins* that are significantly expressed within these presumptive integument clusters (Fig. 3; Supplementary Fig. 8). We found that *gjb3/*Cx35.4, *gjb8/*Cx30.3, *gjb10/*Cx34.4, and *gjc4b/*Cx43.4 are expressed broadly across these clusters (Fig. 3a*ii*). We then looked for *connexins* enriched in subsets of clusters and found unique and specific patterns of expression. Within periderm clusters, we discovered *gjb9a*/Cx28.6, which has not previously been documented in the skin (Fig. 3a*iii*). Within the presumptive neural crest-derived pigment clusters, we found *gja4*/Cx39.4 and *gja5b*/Cx41.8, which are both known to contribute to adult zebrafish skin patterns (Watanabe *et al.* 2016; Watanabe 2017; Fig. 3a*iv*). Within ionocyte clusters, we identified novel expression for 2 *connexins*, *gjb7/*Cx28.8 and *gjb9b*/Cx30.9 (Fig. 3a*v*). Finally, within presumptive basal cell clusters, we found novel expression for 2 *connexins*, *gjc4a.1/*Cx44.2 and *gjc4a.2*/Cx44.5 (Fig. 3a*vi*). These results suggest that the integument uses a complex set of *connexins* throughout organogenesis.

We next examined a subset of the identified integument *connexins* in vivo. We first tested a broadly expressed *connexin*, *gjb8/*Cx30.3, to see if it was expressed in the pigment cells and periderm using fluorescent RNA in situ on 1 dpf embryos. Transverse cross-sections through the trunk revealed prominent *gjb8*/Cx30.3 staining in dorsally located cells near the neural tube and additional dim staining was observed in a single layer of cells surrounding the entire embryo (Fig. 3b*i*). We first confirmed *gjb8*/Cx30.3’s expression in pigment cells by subsetting the 5 clusters that appear to represent pigment cells, including melanophores (Kelsh *et al.* 2000; Parichy *et al.* 2000; *dct+*, clusters 56, 57, Fig. 3b*ii*; Supplementary Table 2) and xanthophores (Parichy *et al.* 2000; *aox5*+, clusters 52, 53, Fig. 3b*ii*; Supplementary Table 2). We find that *gjb8*/Cx30.3 is highly expressed in only the presumptive xanthophore clusters (Fig. 3b*ii*). We then performed fluorescent RNA in situ for *aox5* and *gjb8*/Cx30.3 in a 1 dpf embryo and found robust colocalization of these transcripts, confirming that *gjb8*/Cx30.3 is expressed in xanthophore cells (Fig. 3b*iii*). We then examined *gjb8*/Cx30.3’s expression in the periderm through subsetting the 7 presumptive periderm clusters (*ppl+*, clusters 40–46, Supplementary Table 2) and find expression of *gjb8*/Cx30.3 in all clusters (Fig. 3b*iv*). Indeed, fluorescent RNA in situ for *ppl* and *gjb8*/Cx30.3 reveal robust colocalization of these transcripts in the outermost epithelial layer (Fig. 3b*v*), confirming that *gjb8*/Cx30.3 is expressed in the developing periderm.

We next tested a *connexin* with more specific expression within the integument clusters, *gjb7/*Cx28.8, which has expression specific to the presumptive ionocytes (Fig. 3a*v*). Developing *foxi3a*+ ionocytes form Na+,K+-ATPase-rich (NaR) cells or H+-ATPase-rich (HR) cells, which are characterized by the expression of the specific ATPase genes *atp1b1b* and *atp6v1aa*, respectively (Jänicke *et al.* 2007). First, we subsetted all ionocyte clusters (Jänicke *et al.* 2007; *foxi3a*+, 38, 39, 47, 48) and found unique expression combinations of *atp1b1b* and *atp6v1aa* across clusters and low expression of *gjb7*/Cx28.8 in 3 of 4 clusters (Fig. 3c*i*). Fluorescent RNA in situ revealed colocalization of *gjb7*/Cx28.8 with both *atp1b1b* (Fig. 3c*ii*) and *atp6v1aa* (Fig. 3c*iii*), confirming that *gjb7*/Cx28.8 is expressed in ionocytes. Together, these data confirm the predictive power of the scRNA-seq dataset for *connexin* expression in the integument and support the utility of the dataset as a novel tool for the discovery of investigating *connexin* complexity in vertebrate development.

## Discussion

Here, we reveal the details of *connexin* gene-family expression during zebrafish organogenesis showing that *connexin* usage is widespread yet displays gene-specific variations across tissue, cell type, and developmental time. The large gene family of *connexins* in zebrafish (41 genes) is expressed in complex patterns ranging from nearly ubiquitous to cell-type specific, with unique combinatorial and nested expression sets restricted to individual tissues. Temporally, *connexins* display sustained, increasing, and diminishing expression profiles across development, dependent upon gene and tissue. Together, these data reveal the complexity of expression of this critical gene family in a model vertebrate and demonstrate that this critical form of communication is likely to be used by all tissues during organogenesis. These data provide a critical framework facilitating analysis of how these genes contribute to cellular communication in tissues developing from all germ layers, providing a basis to understand *connexins* in development and in modeling human disease.

We find that all cells express *connexins*, but each tissue expresses a unique combination of the gene family with the composition of the expressed set evolves over developmental time. This spatiotemporal complexity of *connexin* family usage likely contributes to both functional redundancy within tissues as well as functional diversity. The many *connexins* expressed might allow for a myriad of combinatorial interactions amongst Connexin proteins, which could contribute to heteromeric hemichannels and heterotypic GJs. Importantly, Connexins can only interact with potential partners if they are expressed in the same cell or between interacting cells, thus the work here constrains the combinatorial problem of complex usage by revealing the details of the expression patterns through organogenesis. For example, *gjd2a*/Cx35.5 and *gjd1a/*Cx34.1 have been shown to form heterotypic GJs (unique Connexins on each side of the GJ) at electrical synapses of the Mauthner cell neural circuit (Miller *et al.* 2017). The data here show extensive overlapping expression of these 2 *connexins* throughout the central nervous system, suggesting complex hemichannels and GJs could be common throughout the brain. Given that each Connexin-mediated hemichannel has its own unique set of compatibilities and permeability properties, this dataset provides a platform for future research to explore whether *connexins* expressed within the same tissue or cell type form functional channels, and how the molecular identity of these channels influences function.

This dataset presents a powerful resource for zebrafish and *connexin* biology. We establish *connexin* expression in cells previously unknown to express *connexins*, such as the ionocytes of the skin. Within our dataset, there are numerous other cell types with striking *connexin* expression patterns that have under-appreciated *connexin* usage inviting exploration, including macrophages (*gja13.2*/Cx32.2) and PGCs (*gja9a*/Cx55.5, *gjb8*/Cx30.3, *gjc4b*/Cx43.4, and *gjd1b*/Cx34.7). Another strength of this dataset is the exploration of expression across multiple cell types, tissues, and timepoints simultaneously. For example, *gja3*/Cx46 has only been examined in the heart (Chi *et al.* 2008, 2010), yet, in our dataset, we find robust *gja3*/Cx46 expression in both heart and lens clusters, which suggests an enticing link to human GJA3/CX46, in which mutations are associated with cataracts (Mackay *et al.* 1999; Burdon *et al.* 2004; Yao *et al.* 2011). Finally, this dataset provides putative expression to many *connexin* genes that had no previous expression information (22/41 genes). For example, *gjb1a*/Cx27.5 and *gjc2*/Cx47.1 are both highly expressed in the Schwann cell cluster. While neither of these genes had previously known expression information, mutations of their human orthologs GJB1/CX32 and GJC2/CX47 contribute to neuropathy and myelin disorders (López-Bigas *et al.* 2001; Uhlenberg *et al.* 2004; Orthmann-Murphy *et al.* 2007). The identification of tissues and cell-type expression patterns for the entire gene family creates a basis to explore *connexin*-related diseases in zebrafish and provide comparisons to human biology. Through exploring the *connexin* family expression across diverse cell types and tissues, we can begin to envision a holistic view of Connexins utilization and usage in cellular communication throughout organogenesis.

## Data availability

All data generated or analyzed during this study are included in the published article and its supplementary information files. Sequences used in this study were deposited to the NCBI SRA and can be found using the identifier PRJNA564810. Additional files, including the updated GTF, analysis, and code, can be found at https://www.adammillerlab.com/.

Supplemental material is available at *G3* online.

## Supplementary Material

jkac062_Supp_Table1

jkac062_Supp_Table2

jkac062_Supp_Table3

jkac062_Supp_Table4

jkac062_Supp_Table5

jkac062_Supp_Figures

jkac062_Supplemental_Figure_Legend

## References

[jkac062-B1] Alexopoulos H , Böttger A, Fischer S, Levin A, Wolf A, Fujisawa T, Hayakawa S, Gojobori T, Davies JA, David CN, et al; Inx Homarus, Inx Hirudo. Evolution of gap junctions: the missing link? Curr Biol. 2004;14(20):R879–R880. 15498476 10.1016/j.cub.2004.09.067

[jkac062-B2] Baxendale S , Holdsworth CJ, Meza Santoscoy PL, Harrison MRM, Fox J, Parkin CA, Ingham PW, Cunliffe VT. Identification of compounds with anti-convulsant properties in a zebrafish model of epileptic seizures. Dis Model Mech. 2012;5(6):773–784. 22730455 10.1242/dmm.010090 PMC3484860

[jkac062-B3] Beardslee MA , Laing JG, Beyer EC, Saffitz JE. Rapid turnover of connexin43 in the adult rat heart. Circ Res. 1998;83(6):629–635. 9742058 10.1161/01.res.83.6.629

[jkac062-B4] Bergoffen J , Scherer SS, Wang S, Scott MO, Bone LJ, Paul DL, Chen K, Lensch MW, Chance PF, Fischbeck KH. Connexin mutations in X-linked Charcot-Marie-Tooth disease. Science. 1993;262(5142):2039–2042. 8266101 10.1126/science.8266101

[jkac062-B5] Berry V , Mackay D, Khaliq S, Francis PJ, Hameed A, Anwar K, Qasim Mehdi S, Newbold RJ, Ionides A, Shiels A, et al Connexin 50 mutation in a family with congenital “zonular nuclear” pulverulent cataract of Pakistani origin. Hum Genet. 1999;105(1–2):168–170. 10480374 10.1007/s004399900094

[jkac062-B6] Beyer EC , Berthoud VM. Gap junction gene and protein families: connexins, innexins, and pannexins. Biochim Biophys Acta Biomembr. 2018;1860(1):5–8. 28559187 10.1016/j.bbamem.2017.05.016 PMC5704981

[jkac062-B7] Brice G , Ostergaard P, Jeffery S, Gordon K, Mortimer PS, Mansour S. A novel mutation in GJA1 causing oculodentodigital syndrome and primary lymphoedema in a three generation family. Clin Genet. 2013;84(4):378–381. 23550541 10.1111/cge.12158

[jkac062-B8] Brink PR , Cronin K, Banach K, Peterson E, Westphale EM, Seul KH, Ramanan SV, Beyer EC. Evidence for heteromeric gap junction channels formed from rat connexin43 and human connexin37. Am J Physiol. 1997;273(4):C1386–C1396. 9357785 10.1152/ajpcell.1997.273.4.C1386

[jkac062-B9] Bruford EA , Braschi B, Denny P, Jones TEM, Seal RL, Tweedie S. Guidelines for human gene nomenclature. Nat Genet. 2020;52(8):754–758. 32747822 10.1038/s41588-020-0669-3 PMC7494048

[jkac062-B10] Bruzzone R , Haefliger JA, Gimlich RL, Paul DL. Connexin40, a component of gap junctions in vascular endothelium, is restricted in its ability to interact with other connexins. Mol Biol Cell. 1993;4(1):7–20. 8382974 10.1091/mbc.4.1.7 PMC300896

[jkac062-B11] Budi EH , Patterson LB, Parichy DM. Embryonic requirements for ErbB signaling in neural crest development and adult pigment pattern formation. Development. 2008;135(15):2603–2614. 18508863 10.1242/dev.019299 PMC2704560

[jkac062-B12] Burdon KP , Wirth MG, Mackey DA, Russell-Eggitt IM, Craig JE, Elder JE, Dickinson JL, Sale MM. A novel mutation in the Connexin 46 gene causes autosomal dominant congenital cataract with incomplete penetrance. J Med Genet. 2004;41(8):e106. 15286166 10.1136/jmg.2004.018333 PMC1735867

[jkac062-B13] Cason N , White TW, Cheng S, Goodenough DA. Molecular cloning, expression analysis, and functional characterization of connexin44.1 : a zebrafish lens gap junction protein. Dev Dyn. 2001;247:238–247. 10.1002/dvdy.1133 11376491

[jkac062-B14] Chang-Chien J , Yen Y-C, Chien K-H, Li S-Y, Hsu T-C, Yang J-J. The connexin 30.3 of zebrafish homologue of human connexin 26 may play similar role in the inner ear. Hear Res. 2014;313:55–66. 24811980 10.1016/j.heares.2014.04.010

[jkac062-B15] Chanson M , Watanabe M, O’Shaughnessy E, Zoso A, Martin P. Connexin communication compartments and wound repair in epithelial tissue. Int J Mol Sci. 2018;19(5):1354. 10.3390/ijms19051354 PMC5983803 29751558

[jkac062-B16] Charlton-Perkins M , Almeida AD, MacDonald RB, Harris WA. Genetic control of cellular morphogenesis in Müller glia. Glia. 2019;67(7):1401–1411. 30924555 10.1002/glia.23615 PMC6563441

[jkac062-B17] Chatterjee B , Chin AJ, Valdimarsson G, Finis C, Sonntag JM, Choi BY, Tao L, Balasubramanian K, Bell C, Krufka A, et al Developmental regulation and expression of the zebrafish connexin43 gene. Dev Dyn. 2005;233(3):890–906. 15895415 10.1002/dvdy.20426

[jkac062-B18] Chi NC , Bussen M, Brand-Arzamendi K, Ding C, Olgin JE, Shaw RM, Martin GR, Stainier DYR. Cardiac conduction is required to preserve cardiac chamber morphology. Proc Natl Acad Sci USA. 2010;107(33):14662–14667. 20675583 10.1073/pnas.0909432107 PMC2930423

[jkac062-B19] Chi NC , Shaw RM, Jungblut B, Huisken J, Ferrer T, Arnaout R, Scott I, Beis D, Xiao T, Baier H, et al Genetic and physiologic dissection of the vertebrate cardiac conduction system. PLoS Biol. 2008;6(5):e109. 18479184 10.1371/journal.pbio.0060109 PMC2430899

[jkac062-B20] Contreras JE , Sánchez HA, Eugenin EA, Speidel D, Theis M, Willecke K, Bukauskas FF, Bennett MVL, Sáez JC. Metabolic inhibition induces opening of unapposed connexin 43 gap junction hemichannels and reduces gap junctional communication in cortical astrocytes in culture. Proc Natl Acad Sci U S A. 2002;99(1):495–500. 11756680 10.1073/pnas.012589799 PMC117588

[jkac062-B21] Cruciani V , Mikalsen S-O. Evolutionary selection pressure and family relationships among connexin genes. Biol Chem. 2007;388(3):253–264. 17338632 10.1515/BC.2007.028

[jkac062-B22] Dasgupta C , Martinez A-M, Zuppan CW, Shah MM, Bailey LL, Fletcher WH. Identification of connexin43 (α1) gap junction gene mutations in patients with hypoplastic left heart syndrome by denaturing gradient gel electrophoresis (DGGE). Mutat Res. 2001;479(1):173–186. 11470490 10.1016/s0027-5107(01)00160-9

[jkac062-B23] del Castillo I , Villamar M, Moreno-Pelayo MA, del Castillo FJ, Álvarez A, Tellería D, Menéndez I, Moreno F. A deletion involving the connexin 30 gene in nonsyndromic hearing impairment. N Engl J Med. 2002;346(4):243–249. 11807148 10.1056/NEJMoa012052

[jkac062-B24] Denis J-F , Diagbouga MR, Molica F, Hautefort A, Linnerz T, Watanabe M, Lemeille S, Bertrand JY, Kwak BR. KLF4-induced connexin40 expression contributes to arterial endothelial quiescence. Front Physiol. 2019;10:80. 30809154 10.3389/fphys.2019.00080 PMC6379456

[jkac062-B25] Eastman SD , Chen THP, Falk MM, Mendelson TC, Iovine MK. Phylogenetic analysis of three complete gap junction gene families reveals lineage-specific duplications and highly supported gene classes. Genomics. 2006;87(2):265–274. 16337772 10.1016/j.ygeno.2005.10.005

[jkac062-B26] Eisenhoffer GT , Slattum G, Ruiz OE, Otsuna H, Bryan CD, Lopez J, Wagner DS, Bonkowsky JL, Chien C-B, Dorsky RI, et al A toolbox to study epidermal cell types in zebrafish. J Cell Sci. 2017;130(1):269–277. 27149923 10.1242/jcs.184341 PMC5394773

[jkac062-B27] Elfgang C , Eckert R, Lichtenberg-Frate H, Butterweck A, Traub O, Klein RA, Hulser DF, Willecke K. Specific permeability and selective formation of gap junction channels in connexin-transfected HeLa cells. J Cell Biol. 1995;129(3):805–817. 7537274 10.1083/jcb.129.3.805 PMC2120441

[jkac062-B28] Evans WH , Martin PEM. Gap junctions: structure and function (Review). Mol Membr Biol. 2009;19(2):121–136. 10.1080/09687680210139839 12126230

[jkac062-B29] Farnsworth DR , Posner M, Miller AC. Single cell transcriptomics of the developing zebrafish lens and identification of putative controllers of lens development. Exp Eye Res. 2021;206:108535. 33705730 10.1016/j.exer.2021.108535 PMC8092445

[jkac062-B30] Farnsworth DR , Saunders LM, Miller AC. A single-cell transcriptome atlas for zebrafish development. Dev Biol. 2020;459(2):100–108. 31782996 10.1016/j.ydbio.2019.11.008 PMC7080588

[jkac062-B31] Ferrell RE , Baty CJ, Kimak MA, Karlsson JM, Lawrence EC, Franke-Snyder M, Meriney SD, Feingold E, Finegold DN. GJC2 missense mutations cause human lymphedema. Am J Hum Genet. 2010;86(6):943–948. 20537300 10.1016/j.ajhg.2010.04.010 PMC3032064

[jkac062-B32] Figueroa XF , Duling BR. Gap junctions in the control of vascular function. Antioxid Redox Signal. 2009;11(2):251–66. 18831678 10.1089/ars.2008.2117 PMC2933153

[jkac062-B33] Frohnhöfer HG , Geiger-Rudolph S, Pattky M, Meixner M, Huhn C, Maischein H-M, Geisler R, Gehring I, Maderspacher F, Nüsslein-Volhard C, et al Spermidine, but not spermine, is essential for pigment pattern formation in zebrafish. Biol Open. 2016;5(6):736–744. 27215328 10.1242/bio.018721 PMC4920196

[jkac062-B34] Gollob MH , Jones DL, Krahn AD, Danis L, Gong X-Q, Shao Q, Liu X, Veinot JP, Tang ASL, Stewart AFR, et al Somatic mutations in the connexin 40 gene (GJA5) in atrial fibrillation. N Engl J Med. 2006;354(25):2677–2688. 16790700 10.1056/NEJMoa052800

[jkac062-B35] Greb H , Klaassen LJ, Schultz K, Kamermans M, Zoidl G, Weiler R, Janssen-Bienhold U. An alternative splice variant of zebrafish Cx52.6 is expressed in retinal horizontal cells. Neuroscience. 2018;388:191–202. 30048782 10.1016/j.neuroscience.2018.07.024

[jkac062-B36] Grifa A , Wagner CA, D'Ambrosio L, Melchionda S, Bernardi F, Lopez-Bigas N, Rabionet R, Arbones M, Monica MD, Estivill X, et al Mutations in GJB6 cause nonsyndromic autosomal dominant deafness at DFNA3 locus. Nat Genet. 1999;23(1):16–18. 10471490 10.1038/12612

[jkac062-B37] Groenewegen WA , Firouzi M, Bezzina CR, Vliex S, van Langen IM, Sandkuijl L, Smits JPP, Hulsbeek M, Rook MB, Jongsma HJ, et al A cardiac sodium channel mutation cosegregates with a rare connexin40 genotype in familial atrial standstill. Circ Res. 2003;92(1):14–22. 12522116 10.1161/01.res.0000050585.07097.d7

[jkac062-B38] Gross-Thebing T , Paksa A, Raz E. Simultaneous high-resolution detection of multiple transcripts combined with localization of proteins in whole-mount embryos. BMC Biol. 2014;12:55. 25124741 10.1186/s12915-014-0055-7 PMC4172952

[jkac062-B39] Grueterich M , Espana E, Tseng SCG. Connexin 43 expression and proliferation of human limbal epithelium on intact and denuded amniotic membrane. Invest Ophthalmol Vis Sci. 2002;43(1):63–71. 11773014

[jkac062-B40] Guellec DLE , Morvan-Dubois G, Sire J. Skin development in bony fish with particular emphasis on collagen deposition in the dermis of the zebrafish (*Danio rerio*). Int J Dev Biol. 2004;48(2–3):217–231. 15272388 10.1387/ijdb.15272388

[jkac062-B41] Haffter P , Odenthal J, Mullins MC, Lin S, Farrell MJ, Vogelsang E, Haas F, Brand M, van Eeden FJM, Furutani-Seiki M, et al Mutations affecting pigmentation and shape of the adult zebrafish. Dev Genes Evol. 1996;206(4):260–276. 24173565 10.1007/s004270050051

[jkac062-B42] Hansen L , Yao W, Eiberg H, Kjaer KW, Baggesen K, Hejtmancik JF, Rosenberg T. Genetic heterogeneity in microcornea-cataract: five novel mutations in CRYAA, CRYGD, and GJA8. Invest Ophthalmol Vis Sci. 2007;48(9):3937–3944. 17724170 10.1167/iovs.07-0013

[jkac062-B43] Hatler JM , Essner JJ, Johnson RG. A gap junction connexin is required in the vertebrate left–right organizer. Dev Biol. 2009;336(2):183–191. 19799895 10.1016/j.ydbio.2009.09.035

[jkac062-B44] He DS , Jiang JX, Taffet SM, Burt JM. Formation of heteromeric gap junction channels by connexins 40 and 43 in vascular smooth muscle cells. Proc Natl Acad Sci U S A. 1999;96(11):6495–6500. 10339616 10.1073/pnas.96.11.6495 PMC26910

[jkac062-B45] Henke K , Daane JM, Hawkins MB, Dooley CM, Busch-Nentwich EM, Stemple DL, Harris MP. Genetic screen for postembryonic development in the zebrafish (*Danio rerio*): dominant mutations affecting adult form. Genetics. 2017;207(2):609–623. 28835471 10.1534/genetics.117.300187 PMC5629327

[jkac062-B46] Hirata H , Wen H, Kawakami Y, Naganawa Y, Ogino K, Yamada K, Saint-Amant L, Low SE, Cui WW, Zhou W, et al Connexin 39.9 protein is necessary for coordinated activation of slow-twitch muscle and normal behavior in zebrafish. J Biol Chem. 2012;287(2):1080–1089. 22075003 10.1074/jbc.M111.308205 PMC3256877

[jkac062-B47] Hoptak-Solga AD , Nielsen S, Jain I, Thummel R, Hyde DR, Iovine MK. Connexin43 (GJA1) is required in the population of dividing cells during fin regeneration. Dev Biol. 2008;317(2):541–548. 18406403 10.1016/j.ydbio.2008.02.051 PMC2429987

[jkac062-B48] Hu Y , Chen I-P, de Almeida S, Tiziani V, Do Amaral CMR, Gowrishankar K, Passos-Bueno MR, Reichenberger EJ. A novel autosomal recessive GJA1 missense mutation linked to craniometaphyseal dysplasia. PLoS One. 2013;8(8):e73576. 23951358 10.1371/journal.pone.0073576 PMC3741164

[jkac062-B49] Ionasescu V , Ionasescu R, Searby C. Correlation between connexin 32 gene mutations and clinical phenotype in X-linked dominant Charcot-Marie-tooth neuropathy. Am J Med Genet. 1996;63(3):486–491. 8737658 10.1002/(SICI)1096-8628(19960614)63:3<486::AID-AJMG14>3.0.CO;2-I

[jkac062-B50] Iossa S , Marciano E, Franzé A. GJB2 gene mutations in syndromic skin diseases with sensorineural hearing loss. Curr Genomics. 2011;12(7):475–785. 22547955 10.2174/138920211797904098 PMC3219843

[jkac062-B51] Iovine MK , Higgins EP, Hindes A, Coblitz B, Johnson SL. Mutations in connexin43 (GJA1) perturb bone growth in zebrafish fins. Dev Biol. 2005;278(1):208–219. 15649473 10.1016/j.ydbio.2004.11.005

[jkac062-B52] Jänicke M , Carney TJ, Hammerschmidt M. Foxi3 transcription factors and Notch signaling control the formation of skin ionocytes from epidermal precursors of the zebrafish embryo. Dev Biol. 2007;307(2):258–271. 17555741 10.1016/j.ydbio.2007.04.044

[jkac062-B53] Jongsma HJ , Wilders R. Gap junctions in cardiovascular disease. Circ Res. 2000;86(12):1193–1197. 10864907 10.1161/01.res.86.12.1193

[jkac062-B54] Kelsell DP , Dunlop J, Stevens HP, Lench NJ, Liang JN, Parry G, Mueller RF, Leigh IM. Connexin 26 mutations in hereditary non-syndromic sensorineural deafness. Nature. 1997;387(6628):80–83. 9139825 10.1038/387080a0

[jkac062-B55] Kelsh RN , Schmid B, Eisen JS. Genetic analysis of melanophore development in zebrafish embryos. Dev Biol. 2000;225(2):277–293. 10985850 10.1006/dbio.2000.9840

[jkac062-B56] Klaassen LJ , Sun Z, Steijaert MN, Bolte P, Fahrenfort I, Sjoerdsma T, Klooster J, Claassen Y, Shields CR, Ten Eikelder HMM, et al Synaptic transmission from horizontal cells to cones is impaired by loss of connexin hemichannels. PLoS Biol. 2011;9(7):e1001107. 21811399 10.1371/journal.pbio.1001107 PMC3139627

[jkac062-B57] Koval M. Pathways and control of connexin oligomerization. Trends Cell Biol. 2006;16(3):159–166. 16490353 10.1016/j.tcb.2006.01.006 PMC7119061

[jkac062-B58] Koval M , Molina SA, Burt JM. Mix and match: investigating heteromeric and heterotypic gap junction channels in model systems and native tissues. FEBS Lett. 2014;588(8):1193–1204. 24561196 10.1016/j.febslet.2014.02.025 PMC3992227

[jkac062-B59] Lamartine J , Munhoz Essenfelder G, Kibar Z, Lanneluc I, Callouet E, Laoudj D, Lemaître G, Hand C, Hayflick SJ, Zonana J, et al Mutations in GJB6 cause hidrotic ectodermal dysplasia. Nat Genet. 2000;26(2):142–144. 11017065 10.1038/79851

[jkac062-B60] Lasseigne AM , Echeverry FA, Ijaz S, Michel JC, Martin EA, Marsh AJ, Trujillo E, Marsden KC, Pereda AE, Miller AC. Electrical synaptic transmission requires a postsynaptic scaffolding protein. eLife. 2021;10(e66898):1–38. 10.7554/eLife.66898 PMC8081524 33908867

[jkac062-B61] Lawson ND , Li R, Shin M, Grosse A, Yukselen O, Stone OA, Kucukural A, Zhu L. An improved zebrafish transcriptome annotation for sensitive and comprehensive detection of cell type-specific genes. eLife. 2020;9(e55792):1–28. 10.7554/eLife.55792 PMC7486121 32831172

[jkac062-B62] Lee H , Kimelman D. A dominant-negative form of p63 is required for epidermal proliferation in zebrafish. Dev Cell. 2002;2(5):607–616. 12015968 10.1016/s1534-5807(02)00166-1

[jkac062-B63] Li WEI , Waldo K, Linask KL, Chen T, Wessels A, Parmacek MS, Kirby ML, Lo CW. An essential role for connexin43 gap junctions in mouse coronary artery development. Development. 2002;129(8):2031–2042. 11934868 10.1242/dev.129.8.2031

[jkac062-B64] Locke D , Perusinghe N, Newman T, Jayatilake H, Evans WH, Monaghan P. Developmental expression and assembly of connexins into homomeric and heteromeric gap junction hemichannels in the mouse mammary gland. J Cell Physiol. 2000;183(2):228–237. 10737898 10.1002/(SICI)1097-4652(200005)183:2<228::AID-JCP9>3.0.CO;2-Y

[jkac062-B65] López-Bigas N , Olivé M, Rabionet R, Ben-David O, Martínez-Matos JA, Bravo O, Banchs I, Volpini V, Gasparini P, Avraham KB, et al Connexin 31 (GJB3) is expressed in the peripheral and auditory nerves and causes neuropathy and hearing impairment. Hum Mol Genet. 2001;10(9):947–952. 11309368 10.1093/hmg/10.9.947

[jkac062-B66] Macari F , Landau M, Cousin P, Mevorah B, Brenner S, Panizzon R, Schorderet DF, Hohl D, Huber M. Mutation in the gene for connexin 30.3 in a family with erythrokeratodermia variabilis. Am J Hum Genet. 2000;67(5):1296–1301. 11017804 10.1016/s0002-9297(07)62957-7 PMC1288569

[jkac062-B67] Mackay D , Ionides A, Kibar Z, Rouleau G, Berry V, Moore A, Shiels A, Bhattacharya S. Connexin46 mutations in autosomal dominant congenital cataract. Am J Hum Genet. 1999;64(5):1357–1364. 10205266 10.1086/302383 PMC1377871

[jkac062-B68] Maes M , Cogliati B, Crespo Yanguas S, Willebrords J, Vinken M. Roles of connexins and pannexins in digestive homeostasis. Cell Mol Life Sci. 2015a;72(15):2809–2821. 26084872 10.1007/s00018-015-1961-8 PMC4563918

[jkac062-B69] Maes M , Crespo Yanguas S, Willebrords J, Cogliati B, Vinken M. Connexin and pannexin signaling in gastrointestinal and liver disease. Transl Res. 2015b;166(4):332–343. 26051630 10.1016/j.trsl.2015.05.005 PMC4570182

[jkac062-B70] Makita N , Sasaki K, Groenewegen WA, Yokota T, Yokoshiki H, Murakami T, Tsutsui H. Congenital atrial standstill associated with coinheritance of a novel SCN5A mutation and connexin 40 polymorphisms. Heart Rhythm. 2005;2(10):1128–1134. 16188595 10.1016/j.hrthm.2005.06.032

[jkac062-B71] Mikalsen S-O , Tausen M, Kongsstovu SÍ. Phylogeny of teleost connexins reveals highly inconsistent intra- and interspecies use of nomenclature and misassemblies in recent teleost chromosome assemblies. BMC Genomics. 2020;21(1):1–19. 10.1186/s12864-020-6620-2 PMC7066803 32160866

[jkac062-B3354083] Miller AC , Obholzer ND, Shah AN, Megason SG, Moens CB. RNA-seq-based mapping and candidate identification of mutations from forward genetic screens. Genome Res. 2013;23(4):679–686. 23299976 10.1101/gr.147322.112 PMC3613584

[jkac062-B72] Miller AC , Whitebirch AC, Shah AN, Marsden KC, Granato M, O’Brien J, Moens CB. A genetic basis for molecular asymmetry at vertebrate electrical synapses. eLife. 2017;6(e25364):1–24. 10.7554/eLife.25364 PMC5462537 28530549

[jkac062-B73] Misu A , Yamanaka H, Aramaki T, Kondo S, Skerrett IM, Iovine MK, Watanabe M. Two different functions of connexin43 confer two different bone phenotypes in zebrafish. J Biol Chem. 2016;291(24):12601–12611. 27129238 10.1074/jbc.M116.720110 PMC4933477

[jkac062-B74] Nadarajah B , Jones AM, Evans WH, Parnavelas JG. Differential expression of connexins during neocortical development and neuronal circuit formation. J Neurosci. 1997;17(9):3096–3111. 9096144 10.1523/JNEUROSCI.17-09-03096.1997 PMC6573667

[jkac062-B75] Okafo G , Prevedel L, Eugenin E. Tunneling nanotubes (TNT) mediate long-range gap junctional communication: implications for HIV cell to cell spread. Sci Rep. 2017;7(1):1–9. 29192225 10.1038/s41598-017-16600-1 PMC5709493

[jkac062-B76] Okamoto R , Goto I, Nishimura Y, Kobayashi I, Hashizume R, Yoshida Y, Ito R, Kobayashi Y, Nishikawa M, Ali Y, et al Gap junction protein beta 4 plays an important role in cardiac function in humans, rodents, and zebrafish. PLoS One. 2020;15(10):e0240129. 33048975 10.1371/journal.pone.0240129 PMC7553298

[jkac062-B77] Orellana JA , Martinez AD, Retamal MA. Gap junction channels and hemichannels in the CNS: regulation by signaling molecules. Neuropharmacology. 2013;75:567–582. 23499663 10.1016/j.neuropharm.2013.02.020

[jkac062-B78] Orthmann-Murphy JL , Enriquez AD, Abrams CK, Scherer SS. Loss-of-function GJA12/Connexin47 mutations cause Pelizaeus–Merzbacher-like disease. Mol Cell Neurosci. 2007;34(4):629–641. 17344063 10.1016/j.mcn.2007.01.010 PMC1937038

[jkac062-B79] Oyamada M , Oyamada Y, Takamatsu T. Regulation of connexin expression. Biochim Biophys Acta. 2005;1719(1–2):6–23. doi:10.1016/j.bbamem.2005.11.002. 16359940

[jkac062-B80] Parichy DM , Ransom DG, Paw B, Zon LI, Johnson SL. An orthologue of the kit-related gene fms is required for development of neural crest-derived xanthophores and a subpopulation of adult melanocytes in the zebrafish, *Danio rerio*. Development. 2000;127(14):3031–3044. 10862741 10.1242/dev.127.14.3031

[jkac062-B81] Paznekas WA , Boyadjiev SA, Shapiro RE, Daniels O, Wollnik B, Keegan CE, Innis JW, Dinulos MB, Christian C, Hannibal MC, et al Connexin 43 (GJA1) mutations cause the pleiotropic phenotype of oculodentodigital dysplasia. Am J Hum Genet. 2003;72(2):408–418. 12457340 10.1086/346090 PMC379233

[jkac062-B82] Paznekas WA , Karczeski B, Vermeer S, Lowry RB, Delatycki M, Laurence F, Koivisto PA, Van Maldergem L, Boyadjiev SA, Bodurtha JN, et al GJA1 mutations, variants, and connexin 43 dysfunction as it relates to the oculodentodigital dysplasia phenotype. Hum Mutat. 2009;30(5):724–733. 19338053 10.1002/humu.20958 PMC13138855

[jkac062-B83] Ping X , Liang J, Shi K, Bao J, Wu J, Yu X, Tang X, Zou J, Shentu X. Rapamycin relieves the cataract caused by ablation of Gja8b through stimulating autophagy in zebrafish. Autophagy. 2021;17(11):3323–3337. 33472493 10.1080/15548627.2021.1872188 PMC8632074

[jkac062-B84] Plum A , Hallas G, Magin T, Dombrowski F, Hagendorff A, Schumacher B, Wolpert C, Kim J-S, Lamers WH, Evert M, et al Unique and shared functions of different connexins in mice. Curr Biol. 2000;10(18):1083–1091. 10996788 10.1016/s0960-9822(00)00690-4

[jkac062-B85] Polyakov AV , Shagina IA, Khlebnikova OV, Evgrafov OV. Mutation in the connexin 50 gene (GJA8) in a Russian family with zonular pulverulent cataract. Clin Genet. 2001;60(6):476–478. 11846744 10.1034/j.1399-0004.2001.600614.x

[jkac062-B86] Quint WH , Tadema KCD, Vrieze ED, Lukowicz RM, Broekman S, Winkelman BHJ, Hoevenaars M, Gruiter HD, Wijk EV, Schaeffel F, et al Loss of Gap Junction Delta-2 (GJD2) gene orthologs leads to refractive error in zebrafish. Commun Biol. 2021;2:1–14. 10.1038/s42003-021-02185-z PMC8175550 34083742

[jkac062-B87] Richard G. Connexin disorders of the skin. Clin Dermatol. 2005;23(1):23–32. 15708286 10.1016/j.clindermatol.2004.09.010

[jkac062-B88] Richard G , Brown N, Smith LE, Terrinoni A, Melino G, Mackie RM, Bale SJ, Uitto J. The spectrum of mutations in erythrokeratodermias–novel and de novo mutations in GJB3. Hum Genet. 2000;106(3):321–329. 10798362 10.1007/s004390051045

[jkac062-B89] Richard G , Rouan F, Willoughby CE, Brown N, Chung P, Ryynänen M, Jabs EW, Bale SJ, DiGiovanna JJ, Uitto J, et al Missense mutations in GJB2 encoding connexin-26 cause the ectodermal dysplasia keratitis-ichthyosis-deafness syndrome. Am J Hum Genet. 2002;70(5):1341–1348. 11912510 10.1086/339986 PMC447609

[jkac062-B90] Richard G , Smith LE, Bailey RA, Itin P, Hohl D, Epstein EH, DiGiovanna JJ, Compton JG, Bale SJ. Mutations in the human connexin gene GJB3 cause erythrokeratodermia variabilis. Nat Genet. 1998;20(4):366–369. 9843209 10.1038/3840

[jkac062-B91] Rozental R , Giaume C, Spray DC. Gap junctions in the nervous system. Brain Res Brain Res Rev. 2000;32(1):11–15. 10928802 10.1016/s0165-0173(99)00095-8

[jkac062-B92] Satija R , Farrell JA, Gennert D, Schier AF, Regev A. Spatial reconstruction of single-cell gene expression data. Nat Biotechnol. 2015;33(5):495–502. 25867923 10.1038/nbt.3192 PMC4430369

[jkac062-B93] Snoeckx RL , Huygen PLM, Feldmann D, Marlin S, Denoyelle F, Waligora J, Mueller-Malesinska M, Pollak A, Ploski R, Murgia A, et al GJB2 mutations and degree of hearing loss: a multicenter study. Am J Hum Genet. 2005;77(6):945–957. 16380907 10.1086/497996 PMC1285178

[jkac062-B94] Soares AR , Martins-Marques T, Ribeiro-Rodrigues T, Vasco J, Catarino S, Pinho MJ, Zuzarte M, Anjo SI, Manadas B, Sluijter JPG, et al Gap junctional protein Cx43 is involved in the communication between extracellular vesicles and mammalian cells. Nature. 2015;5(13243):1–13. 10.1038/srep13243 PMC4541155 26285688

[jkac062-B95] Sprague J , Doerry E, Douglas S, Westerfield M. The Zebrafish Information Network (ZFIN): a resource for genetic, genomic and developmental research. Nucleic Acids Res. 2001;29(1):87–90. 11125057 10.1093/nar/29.1.87 PMC29808

[jkac062-B96] Srinivas M , Rozental R, Kojima T, Dermietzel R, Mehler M, Condorelli DF, Kessler J. A, Spray DC. Functional properties of channels formed by the neuronal gap junction protein connexin36. J Neurosci. 1999;19(22):9848–9855. 10559394 10.1523/JNEUROSCI.19-22-09848.1999 PMC6782942

[jkac062-B97] Sultana N , Nag K, Hoshijima K, Laird DW, Kawakami A, Hirose S. Zebrafish early cardiac connexin, Cx36.7/Ecx, regulates myofibril orientation and heart morphogenesis by establishing Nkx2.5 expression. Proc Natl Acad Sci U S A. 2008;105(12):4763–4768. 18337497 10.1073/pnas.0708451105 PMC2290751

[jkac062-B98] Tarzemany R , Jiang G, Jiang JX, Larjava H, Häkkinen L. Connexin 43 hemichannels regulate the expression of wound healing-associated genes in human gingival fibroblasts. Sci Rep. 2017;7(1):15. 29074845 10.1038/s41598-017-12672-1 PMC5658368

[jkac062-B99] Temme A , Buchmann A, Gabriel H-D, Nelles E, Schwarz M, Willecke K. High incidence of spontaneous and chemically induced liver tumors in mice deficient for connexin32. Curr Biol. 1997;7(9):713–716. 9285723 10.1016/s0960-9822(06)00302-2

[jkac062-B100] Thisse B , Pflumio S, Fürthauer M, Loppin B, Heyer V, Degrave A, Woehl R, Lux A, Steffan T, Charbonnier XQ, et al Expression of the zebrafish genome during embryogenesis. ZFIN Direct Data Submission. 2001.

[jkac062-B101] Thisse B , Thisse C. Fast release clones: a high throughput expression analysis. ZFIN Direct Data Submission. 2004.

[jkac062-B102] Thisse C , Thisse B. High throughput expression analysis of ZF-Models consortium clones. ZFIN Direct Data Submission. 2005.

[jkac062-B103] Tishchenko A , Azor DD, Vidal-Brime L. Cx43 and associated cell signaling pathways. Cancers. 2020;12(10):1–25. 33003486 10.3390/cancers12102798 PMC7601615

[jkac062-B104] Uhlenberg B , Schuelke M, Rüschendorf F, Ruf N, Kaindl AM, Henneke M, Thiele H, Stoltenburg-Didinger G, Aksu F, Topaloğlu H, et al Mutations in the gene encoding gap junction protein α12 (connexin 46.6) cause Pelizaeus-Merzbacher–like disease. Am J Hum Genet. 2004;75(2):251–260. 15192806 10.1086/422763 PMC1216059

[jkac062-B105] Wang J , Lin Z-J, Liu L, Xu H-Q, Shi Y-W, Yi Y-H, He N, Liao W-P. Epilepsy-associated genes. Seizure. 2017;44:11–20. 28007376 10.1016/j.seizure.2016.11.030

[jkac062-B106] Watanabe M. Gap junction in the teleost fish lineage: duplicated connexins may contribute to skin pattern formation and body shape determination. Front Cell Dev Biol. 2017;5:13–18. 28271062 10.3389/fcell.2017.00013 PMC5318405

[jkac062-B107] Watanabe M , Sawada R, Aramaki T, Skerrett IM, Kondo S. The physiological characterization of connexin41.8 and connexin39.4, which are involved in the striped pattern formation of zebrafish. J Biol Chem. 2016;291(3):1053–1063. 26598520 10.1074/jbc.M115.673129 PMC4714190

[jkac062-B108] Weber PA , Chang HC, Spaeth KE, Nitsche JM, Nicholson BJ. The permeability of gap junction channels to probes of different size is dependent on connexin composition and permeant-pore affinities. Biophys J. 2004;87(2):958–973. 15298902 10.1529/biophysj.103.036350 PMC1304503

[jkac062-B109] Wei C , Xu X, Lo CW. Connexins and cell signaling in development and disease. Annu Rev Cell Dev Biol. 2004;20:811–838. 15473861 10.1146/annurev.cellbio.19.111301.144309

[jkac062-B110] Westerfield M. A Guide for the Laboratory Use of Zebrafish Danio (Brachydanio) Rerio. 5th ed. Eugene: University of Oregon Press; 2000.

[jkac062-B111] Wierson WA , Welker JM, Almeida MP, Mann CM, Webster DA, Torrie ME, Weiss TJ, Kambakam S, Vollbrecht MK, Lan M, et al Efficient targeted integration directed by short homology in zebrafish and mammalian cells. eLife. 2020;9(e53968):1–25. 10.7554/eLife.53968 PMC7228771 32412410

[jkac062-B112] Willems PJ. Genetic Causes of Hearing Loss. N Engl J Med. 2000;342(15):1101–1109. 10760311 10.1056/NEJM200004133421506

[jkac062-B113] Willoughby CE , Arab Sara Gandhi R, Zeinali S, Arab Seddigheh Luk D, Billingsley G, Munier FL, Héon E. A novel mutation in an Iranian family with progressive autosomal dominant congenital nuclear cataract. J Med Genet. 2003;40(11):e124e. 14627691 10.1136/jmg.40.11.e124 PMC1735309

[jkac062-B114] Wirka RC , Gore S, Van Wagoner DR, Arking DE, Lubitz SA, Lunetta KL, Benjamin EJ, Alonso A, Ellinor PT, Barnard J, et al A common connexin-40 gene promoter variant affects connexin-40 expression in human atria and is associated with atrial fibrillation. Circ Arrhythm Electrophysiol. 2011;4(1):87–93. 21076161 10.1161/CIRCEP.110.959726 PMC3057452

[jkac062-B115] Xia J , Liu C, Tang B, Pan Q, Huang L, Dai H, Zhang B, Xie W, Hu D, Zheng D, et al Mutations in the gene encoding gap junction protein β-3 associated with autosomal dominant hearing impairment. Nat Genet. 1998;20(4):370–373. 9843210 10.1038/3845

[jkac062-B116] Xing L , Yang T, Cui S, Chen G. Connexin hemichannels in astrocytes: role in CNS disorders. Front Mol Neurosci. 2019;12:23. 30787868 10.3389/fnmol.2019.00023 PMC6372977

[jkac062-B117] Yang YHC , Briant LJB, Raab C, Mullapudi ST, Maischein H-M, Kawakami K, Stainier DYR. Innervation modulates the functional connectivity between pancreatic endocrine cells. bioRxiv. 2020. 10.1101/2020.11.04.368084 PMC9007585 35373736

[jkac062-B118] Yang Y-Q , Zhang X-L, Wang X-H, Tan H-W, Shi H-F, Jiang W-F, Fang W-Y, Liu X. Connexin40 nonsense mutation in familial atrial fibrillation. Int J Mol Med. 2010;26(4):605–610. 20818502 10.3892/ijmm_00000505

[jkac062-B119] Yao K , Wang W, Zhu Y, Jin C, Shentu X, Jiang J, Zhang Y, Ni S. A novel GJA3 mutation associated with congenital nuclear pulverulent and posterior polar cataract in a Chinese family. Hum Mutat. 2011;32(12):1367–1370. 21681855 10.1002/humu.21552

[jkac062-B120] Yeh H-I , Chou Y, Liu H-F, Chang S-C, Tsai C-H. Connexin37 gene polymorphism and coronary artery disease in Taiwan. Int J Cardiol. 2001;81(2–3):251–255. 11744143 10.1016/s0167-5273(01)00574-5

[jkac062-B121] Yoshikawa S , Vila A, Segelken J, Lin Y, Mitchell CK, Nguyen D, Brien JO. Zebrafish connexin 79.8 (Gja8a): a lens connexin used as an electrical synapse in some neurons. Devel Neurobio. 2017;77(5):548–561. 10.1002/dneu.22418 PMC5226913 27402207

[jkac062-B122] Zhang J , Chandrasekaran G, Li W, Kim D, Jeong IY, Lee S, Liang T, Bae JY, Choi I, Kang H, et al Wnt-PLC-IP3-Connexin-Ca2+ axis maintains ependymal motile cilia in zebrafish spinal cord. Nat Commun. 2020;11(1860):1–14. 32312952 10.1038/s41467-020-15248-2 PMC7170879

[jkac062-B123] Zheng GXY , Terry JM, Belgrader P, Ryvkin P, Bent ZW, Wilson R, Ziraldo SB, Wheeler TD, McDermott GP, Zhu J, et al Massively parallel digital transcriptional profiling of single cells. Nat Commun. 2017;8(1):14049. 28091601 10.1038/ncomms14049 PMC5241818

