# Supplementary material for: Connexinplexity: the spatial and temporal expression of connexin genes during vertebrate organogenesis
Source: G3 (Bethesda). 2022 Mar 24;12(5):jkac062. doi: 10.1093/g3journal/jkac062 (PMC9073686; doi:10.1093/g3journal/jkac062)

Supplemental Figure 1

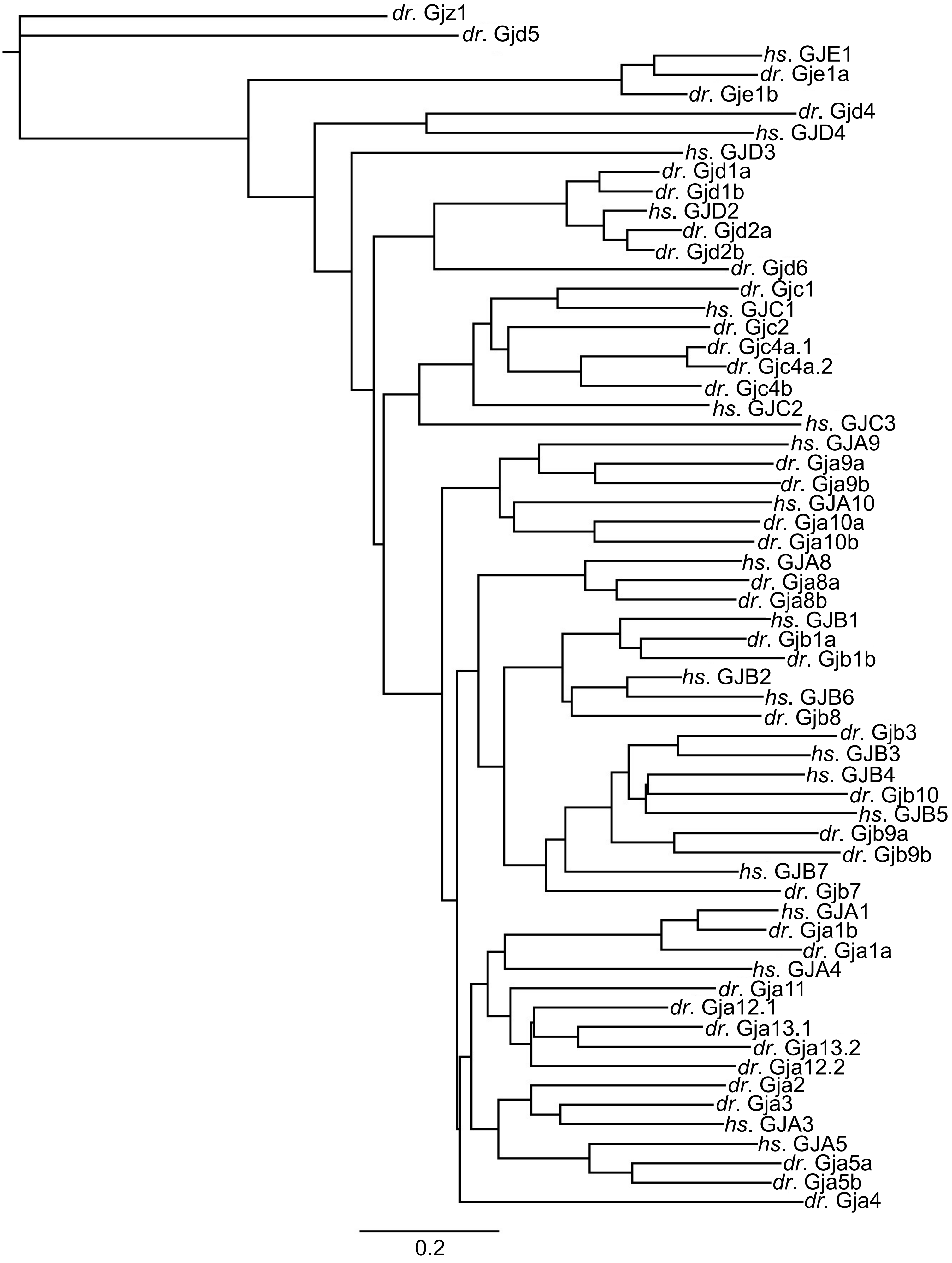

Supplemental Figure 2

A

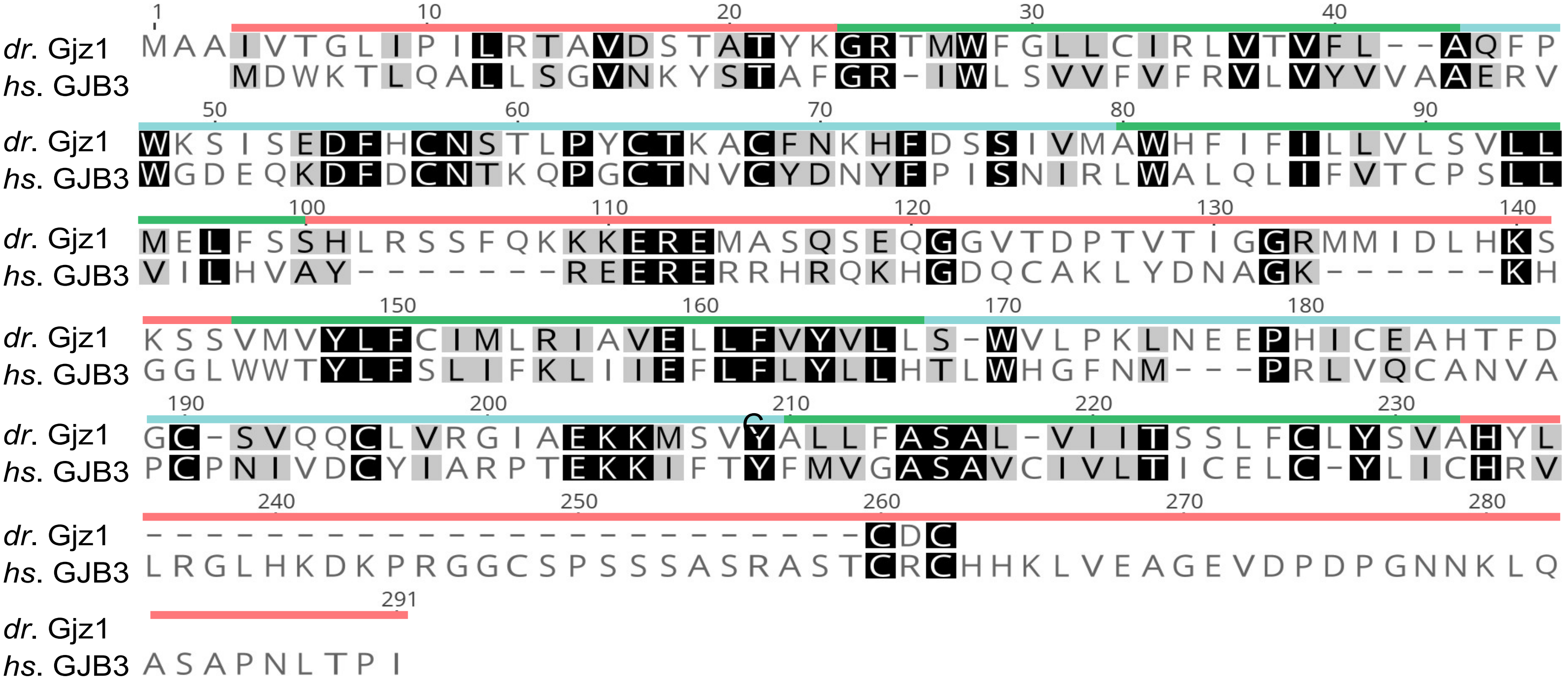

B

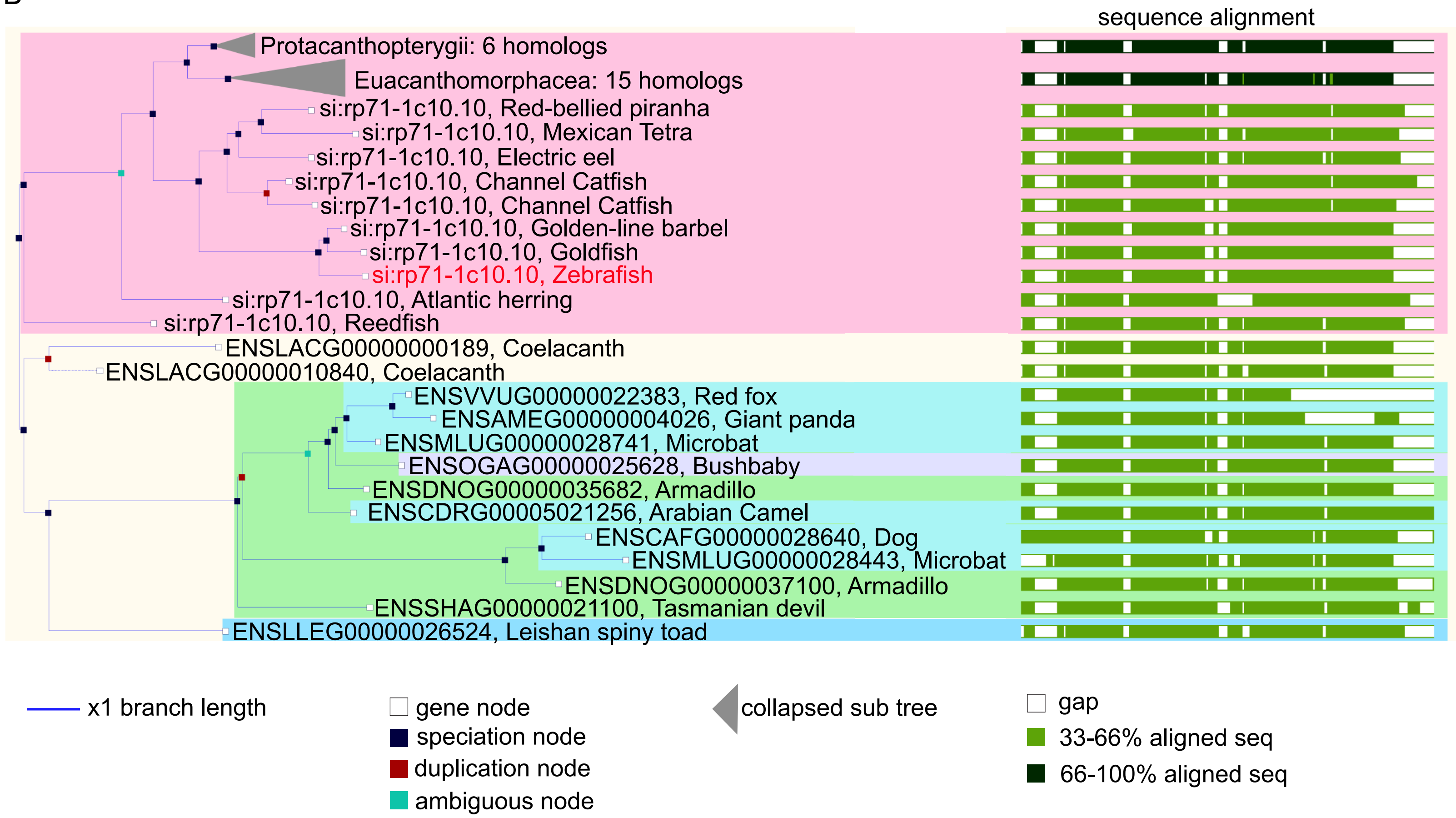

Supplemental Figure 3

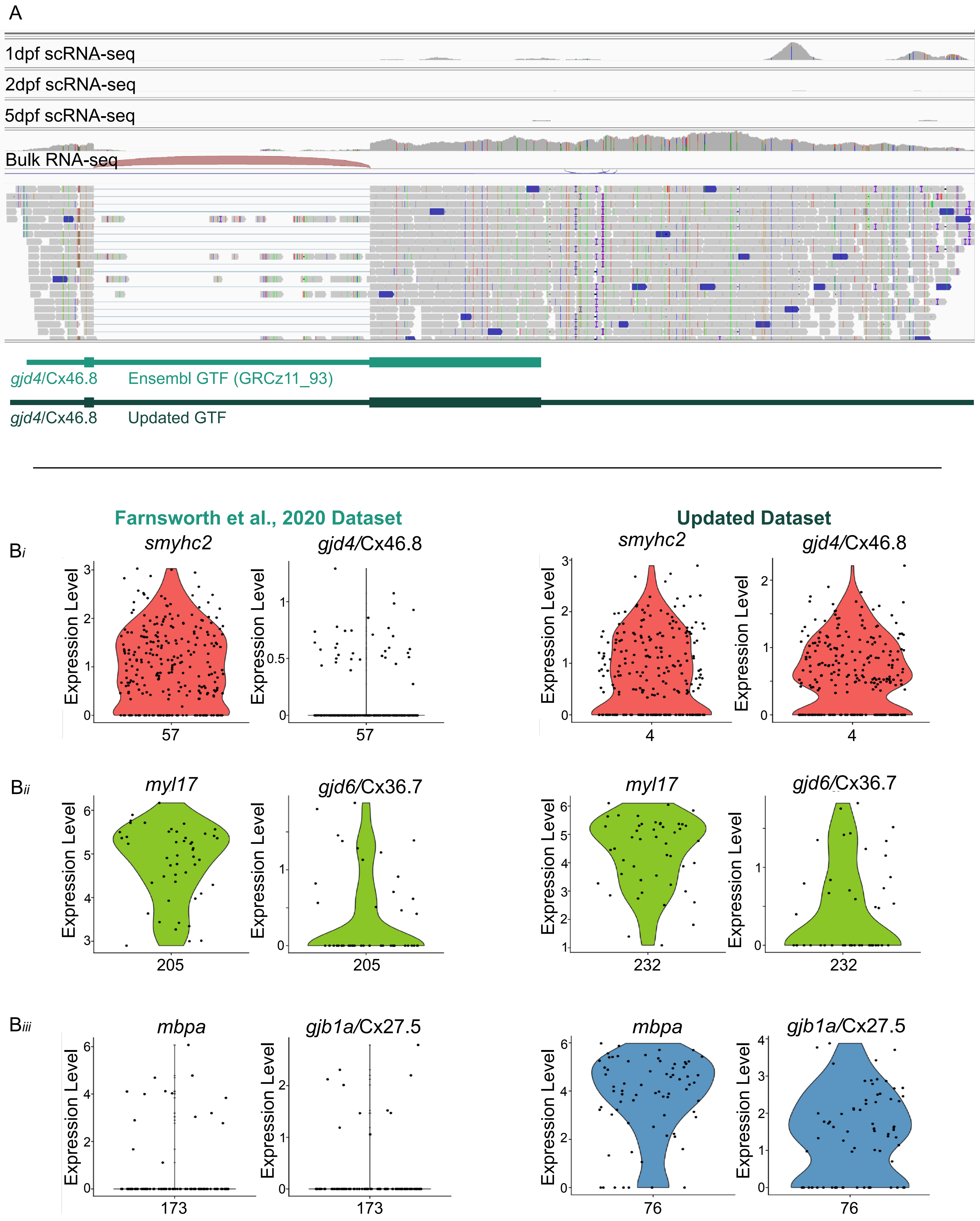

Supplemental Figure 4

A

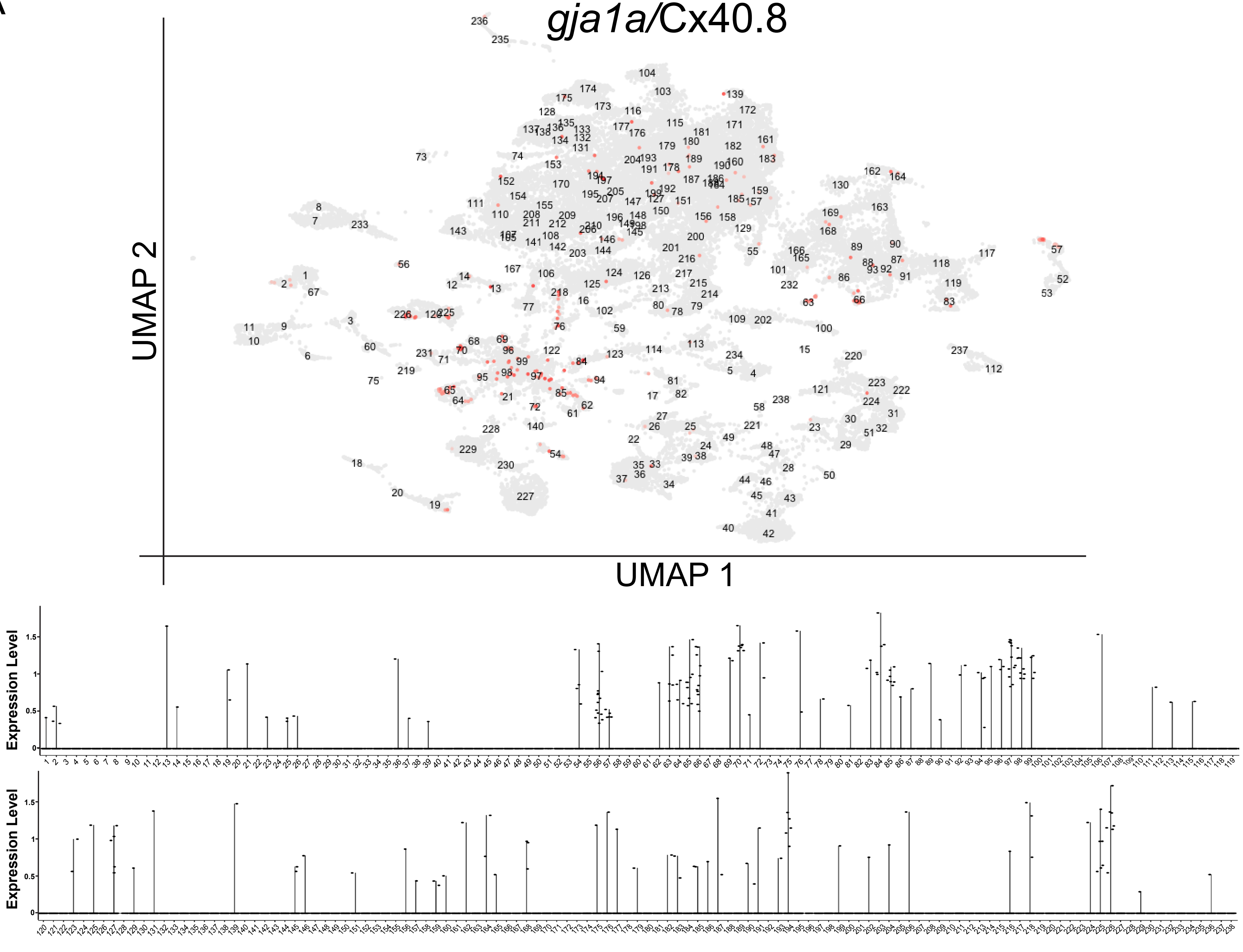

B

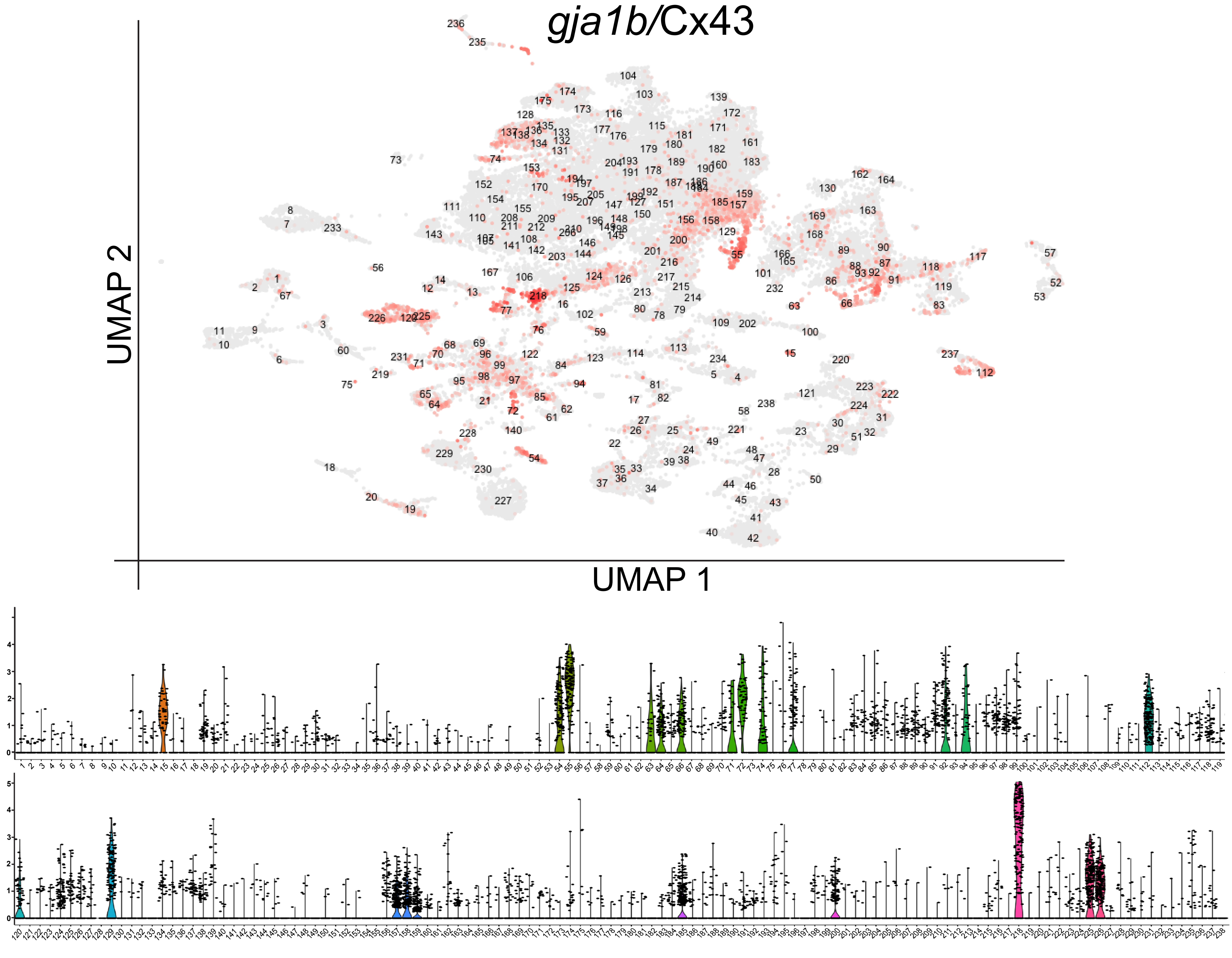

C

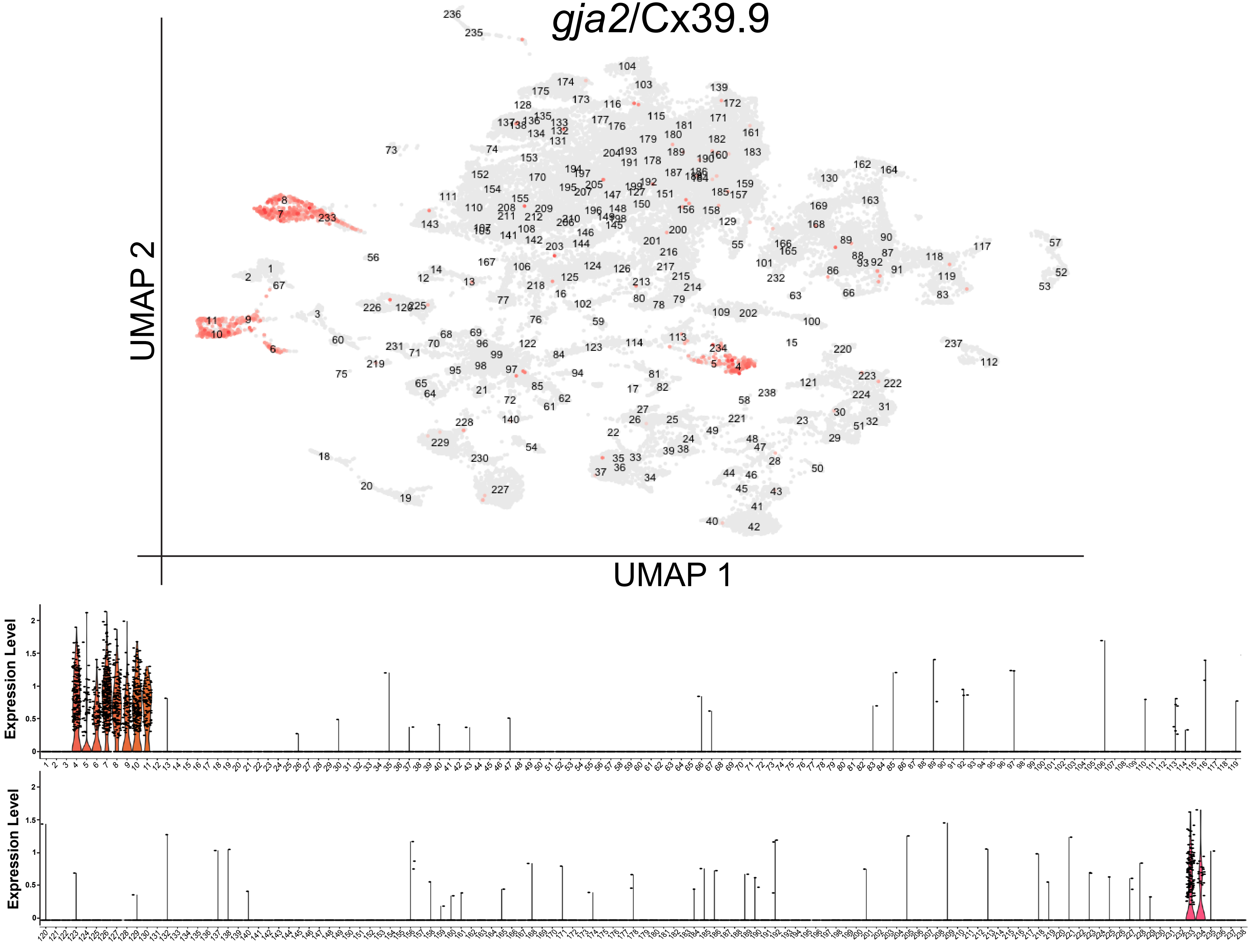

D

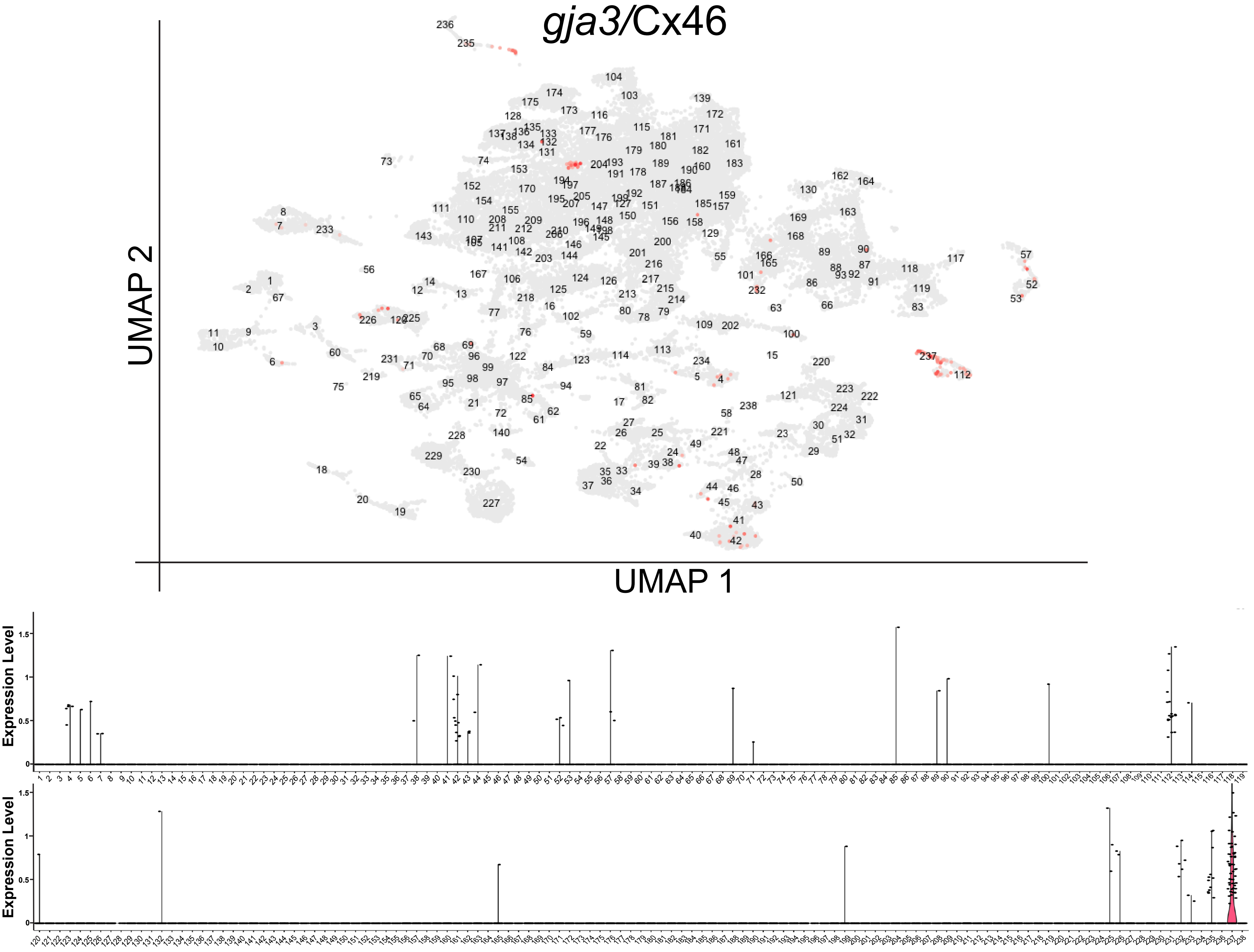

E

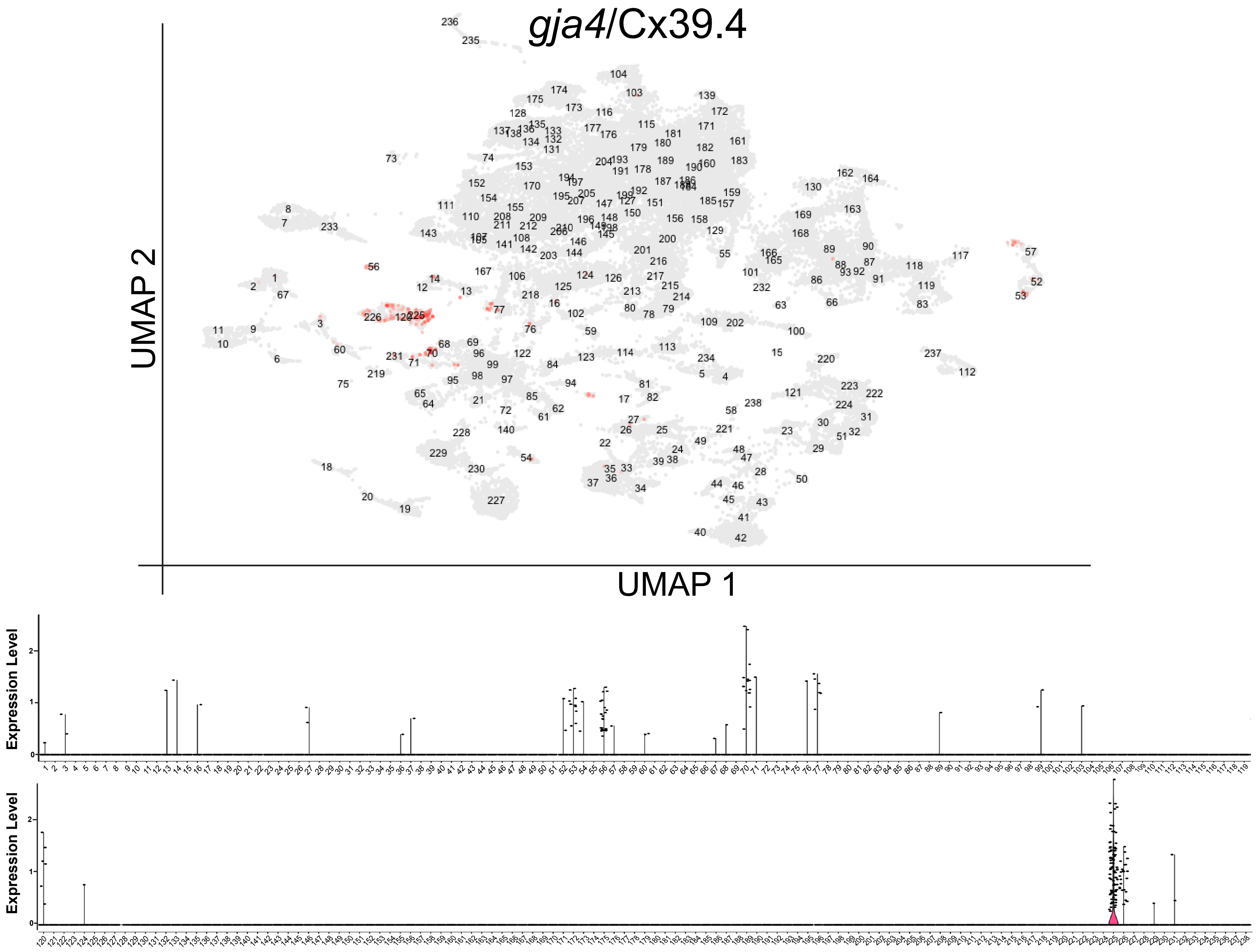

F

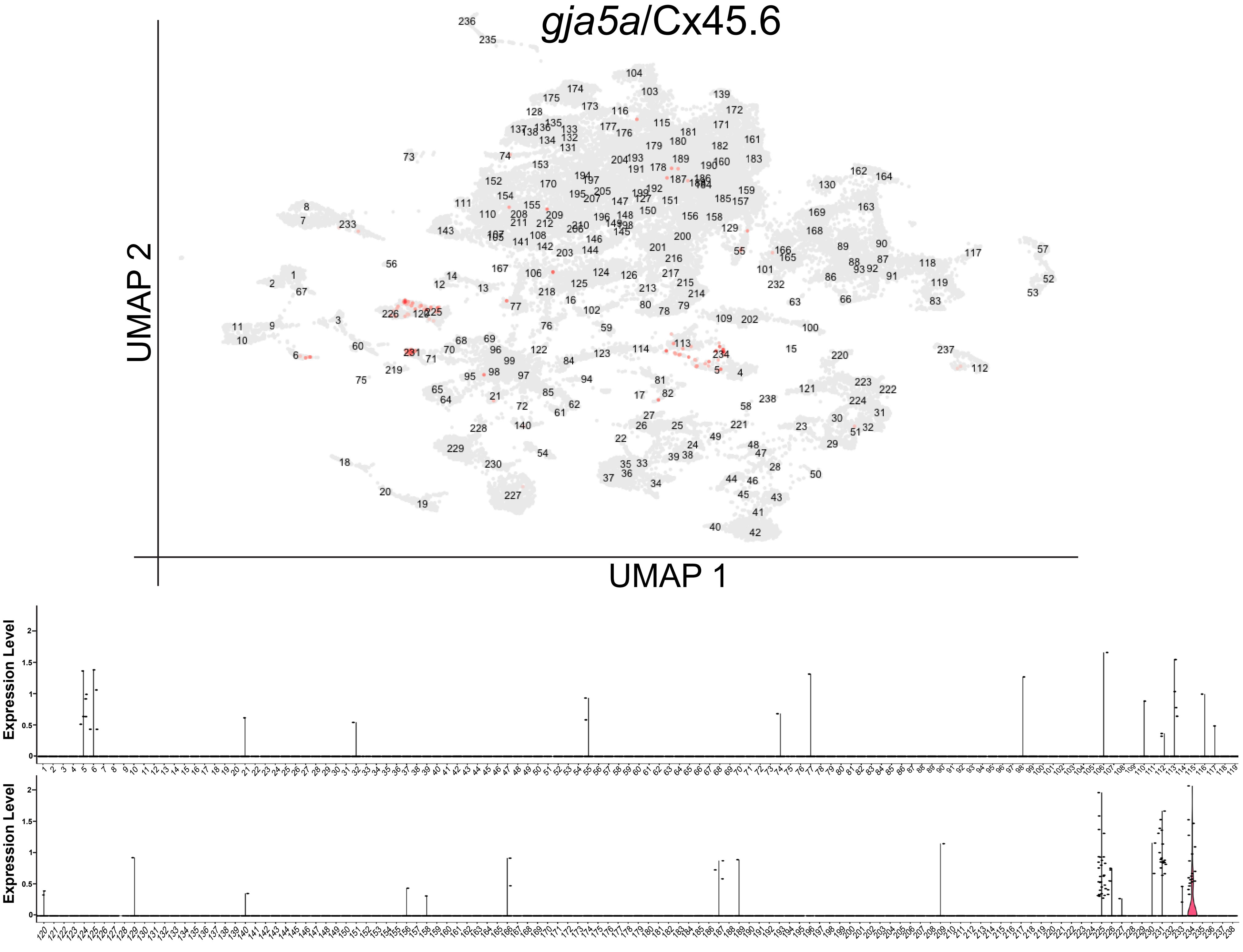
gja5a/Cx45.6
UMAP 2
UMAP 1
Expression Level
Expression Level

G

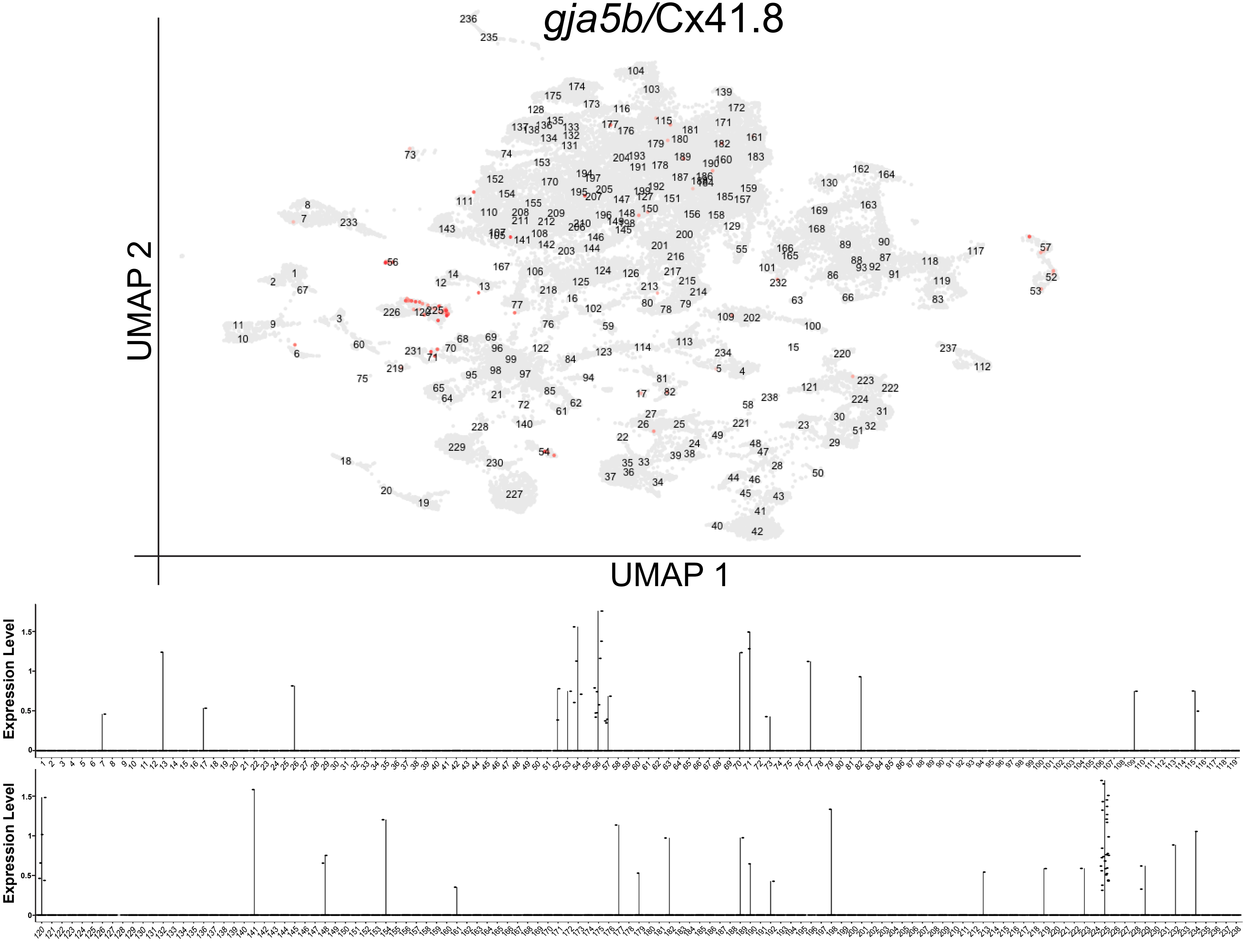
gja5b/Cx41.8
UMAP 2
UMAP 1
Expression Level
Expression Level

H

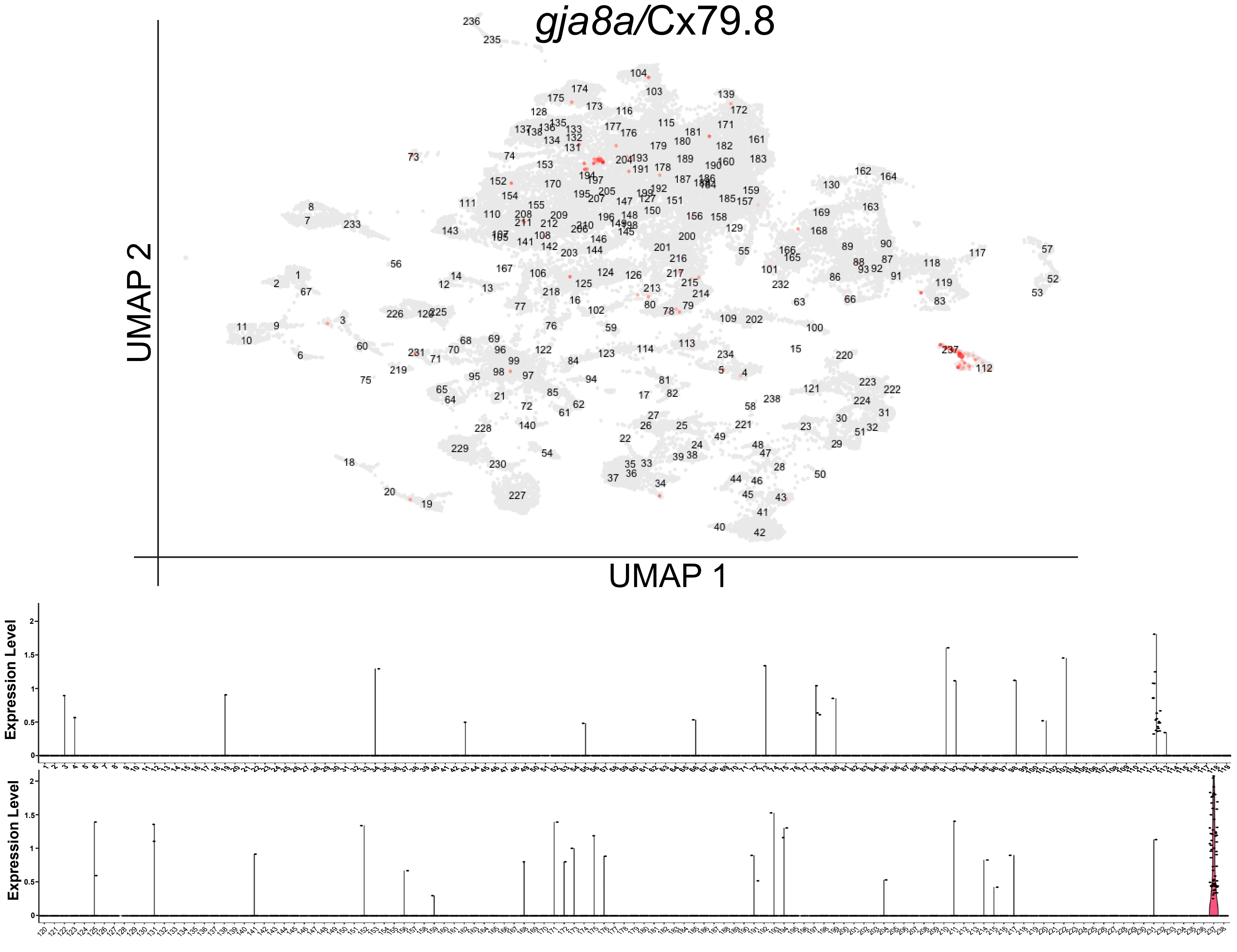
gja8a/Cx79.8
UMAP 2
UMAP 1
Expression Level
Expression Level

I

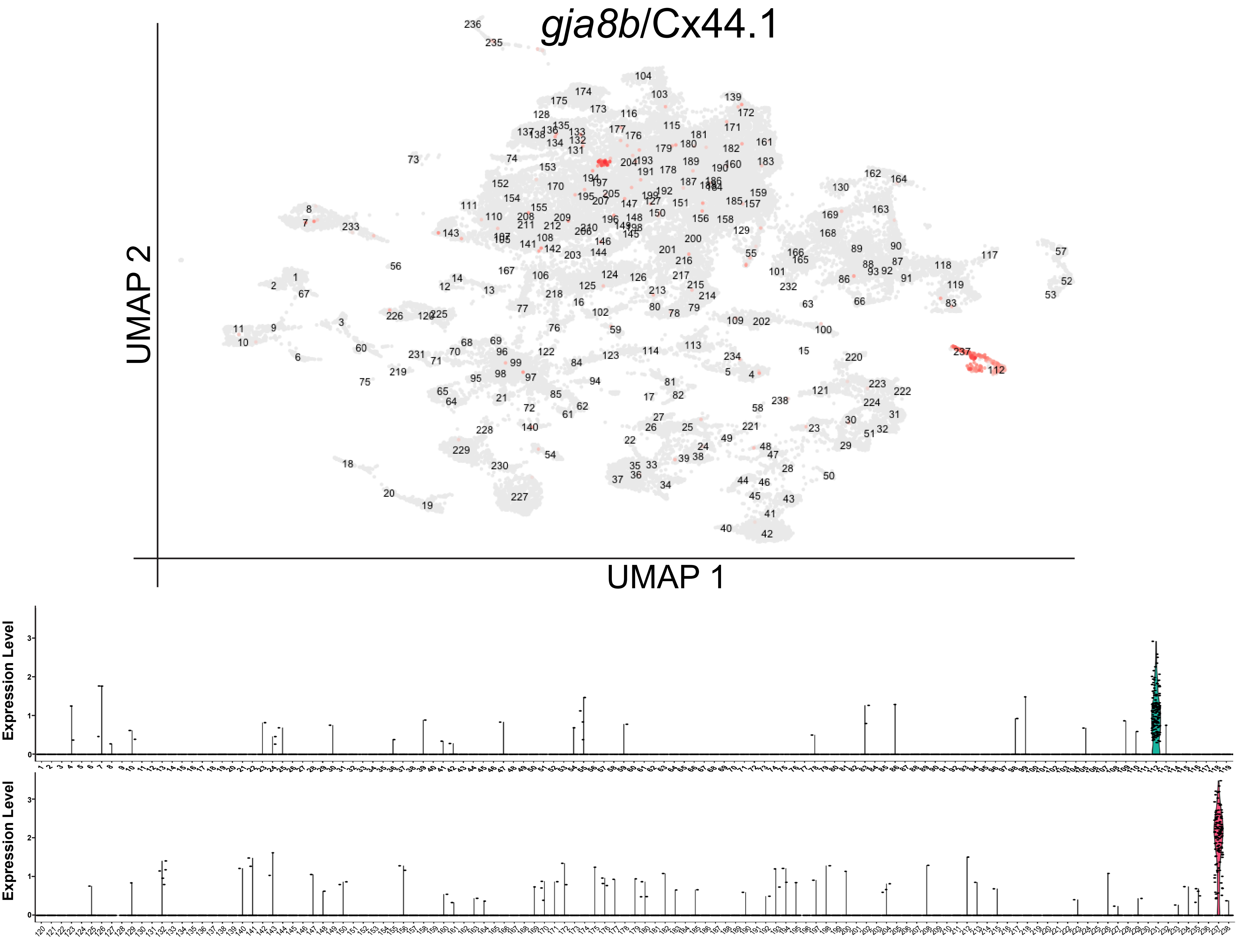

gja8b/Cx44.1
UMAP 2
UMAP 1
Expression Level
Expression Level

J

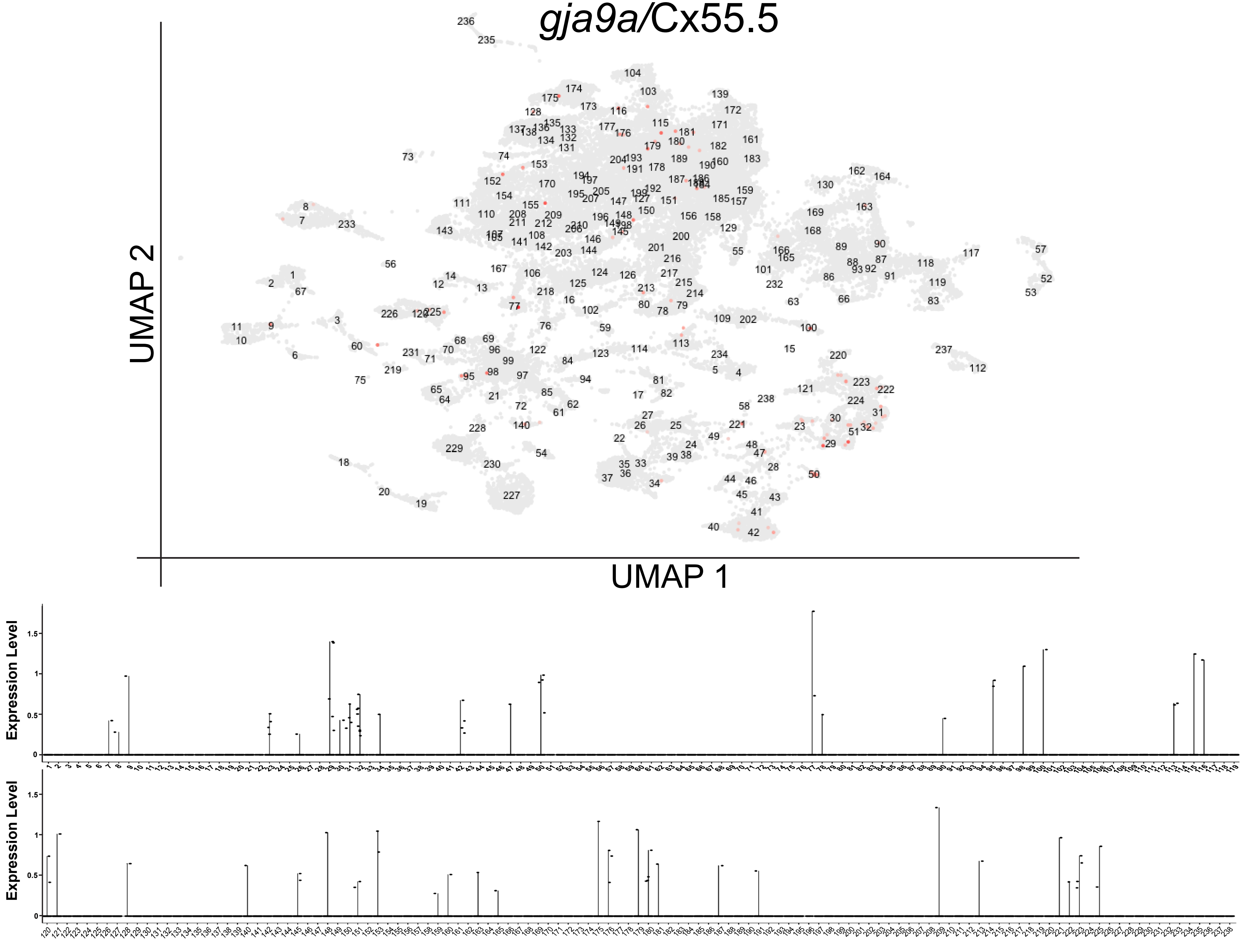

gja9a/Cx55.5
UMAP 2
UMAP 1
Expression Level
Expression Level

K

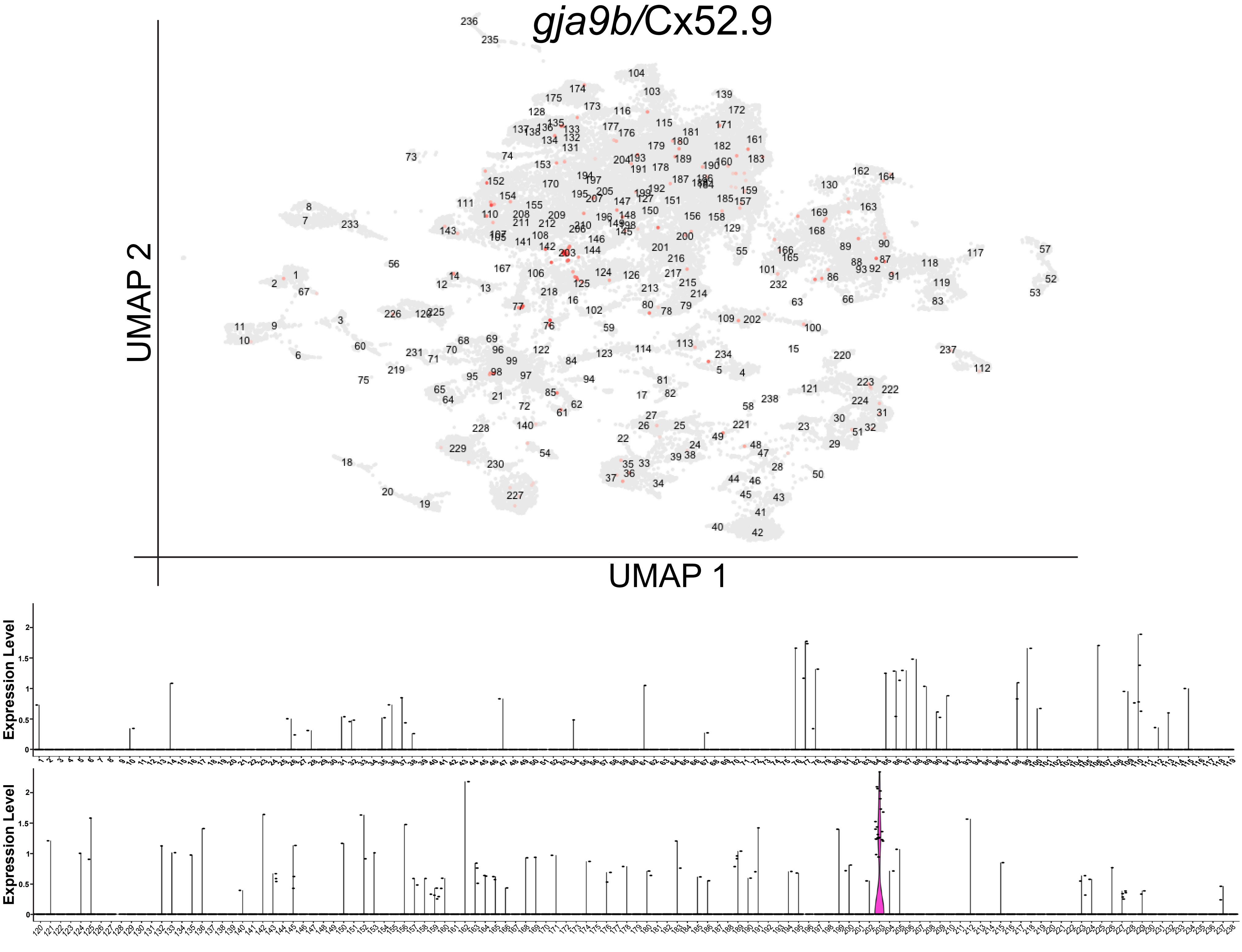

gja9b/Cx52.9
UMAP 2
UMAP 1
Expression Level
0
0.5
1
1.5
2
Expression Level
0
0.5
1
1.5
2

L

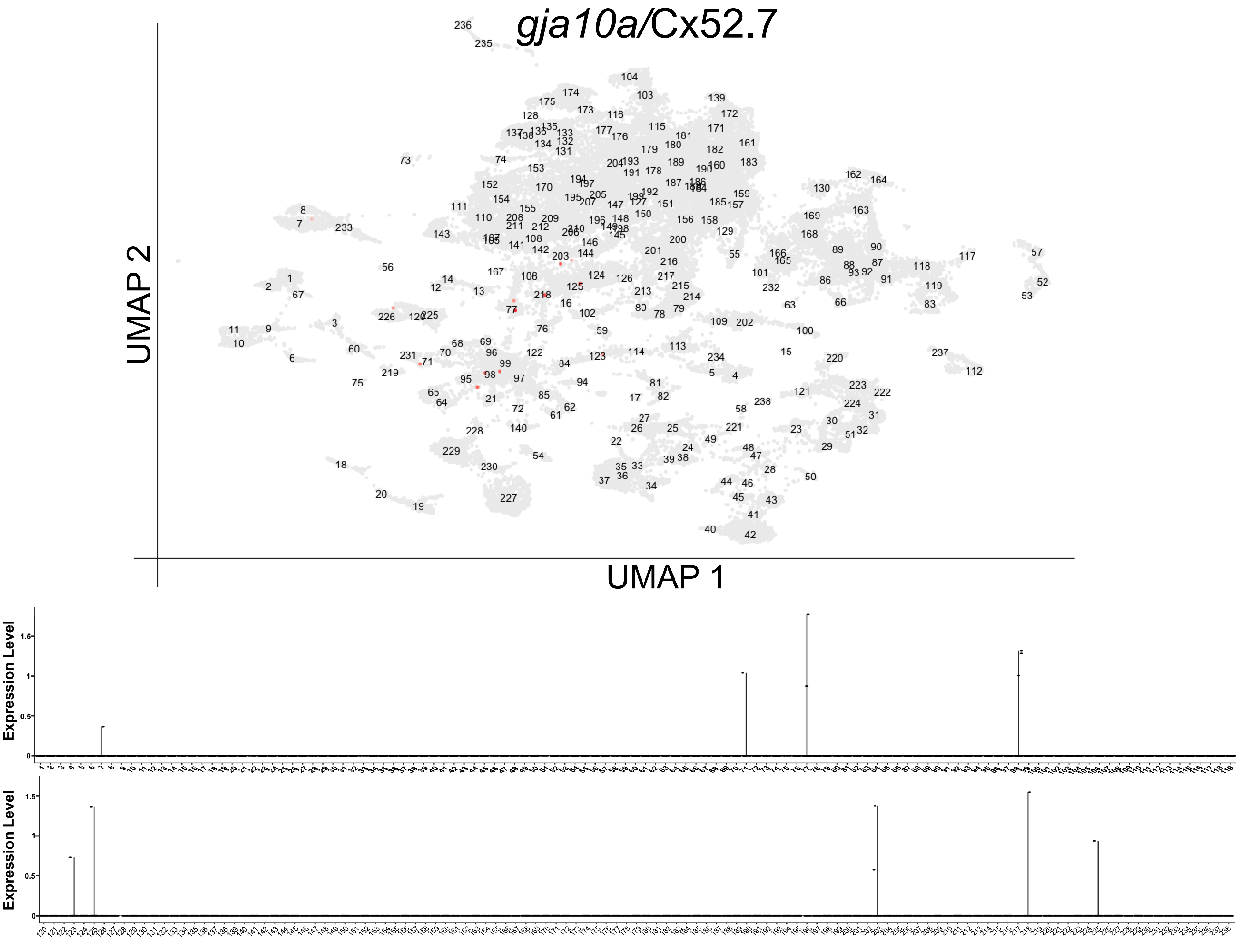
gja10a/Cx52.7
UMAP 2
UMAP 1
Expression Level
Expression Level

M

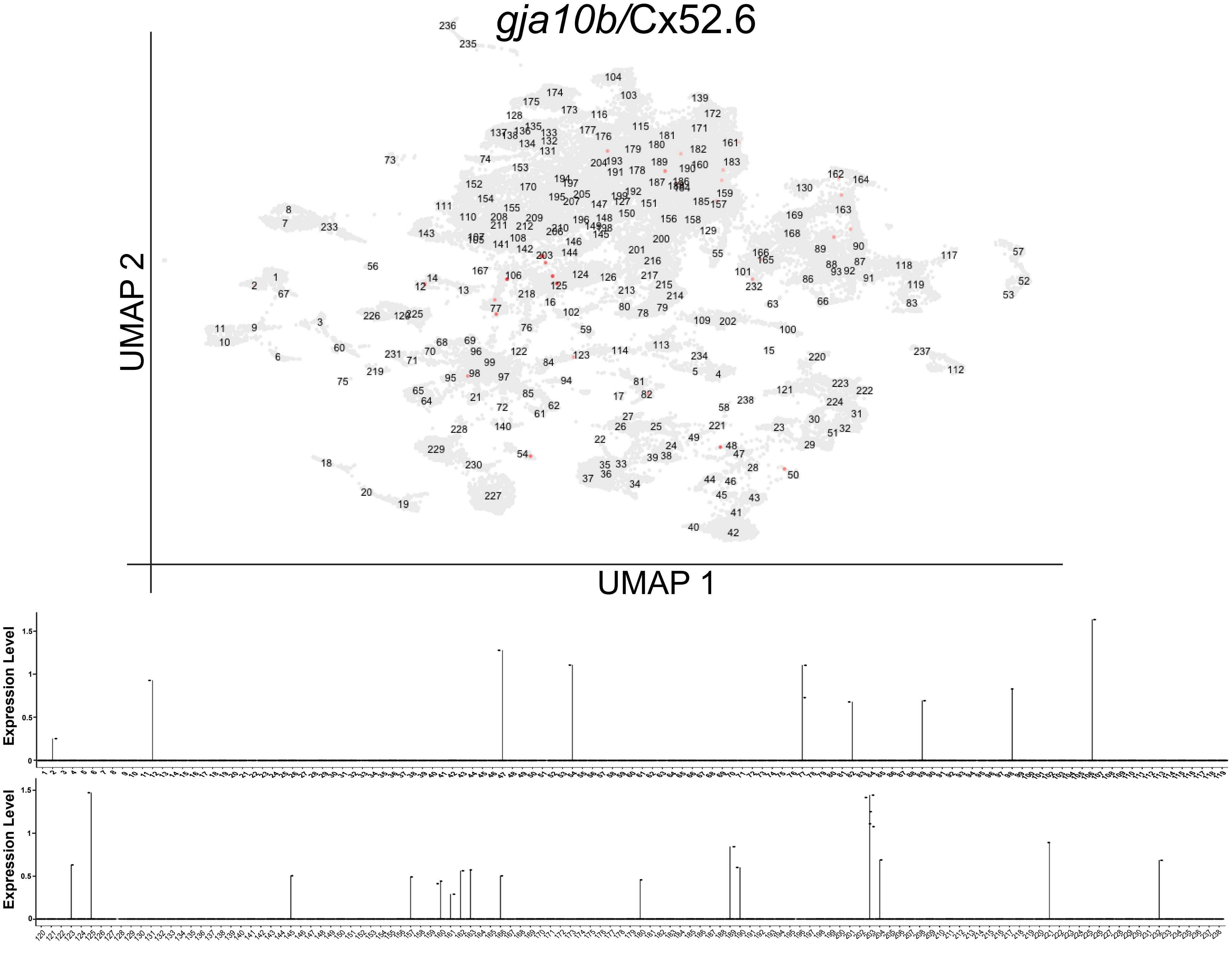

gja10b/Cx52.6
UMAP 2
UMAP 1
Expression Level
Expression Level

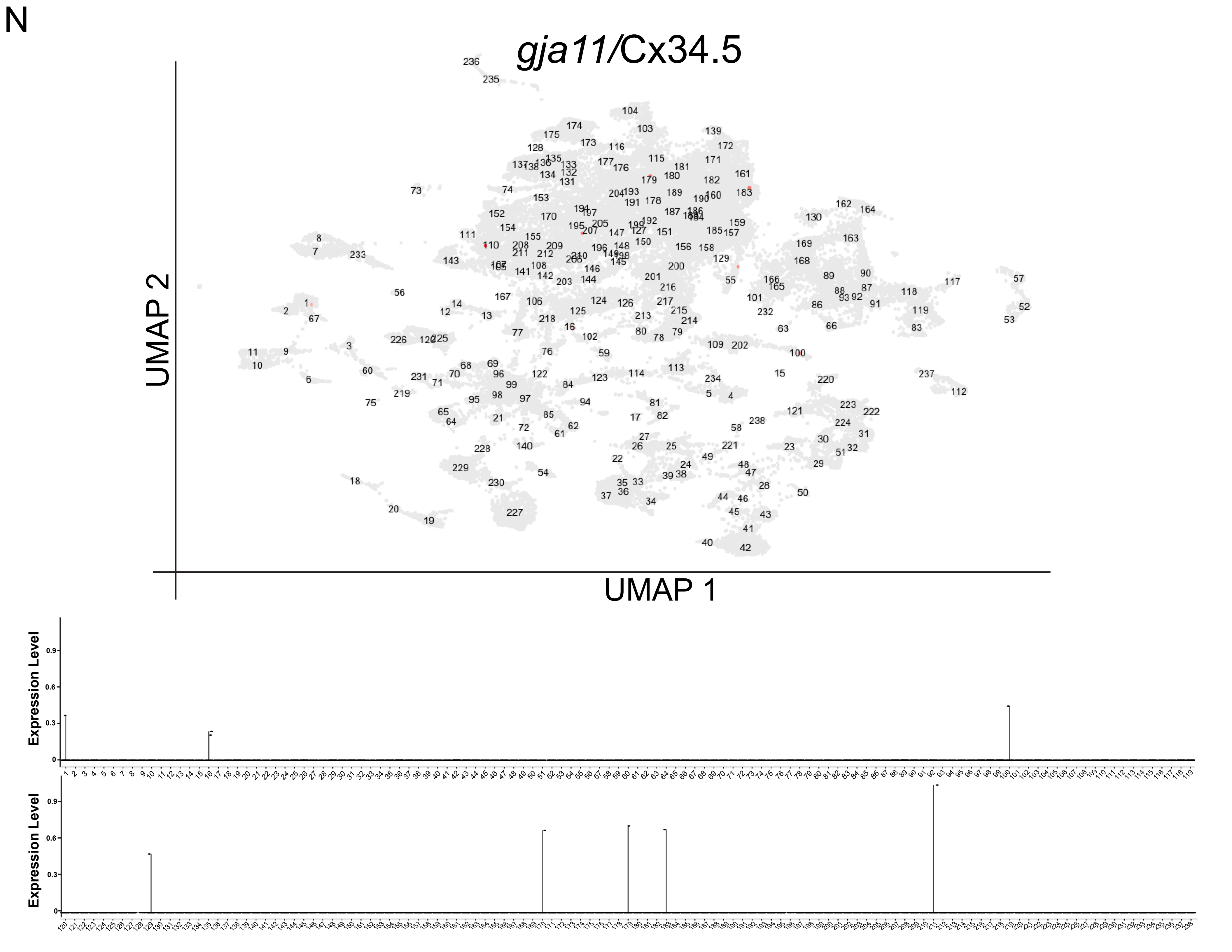
N
gja11/Cx34.5
UMAP 2
UMAP 1
Expression Level
0.9
0.6
0.3
0
Expression Level
0.9
0.6
0.3
0

O

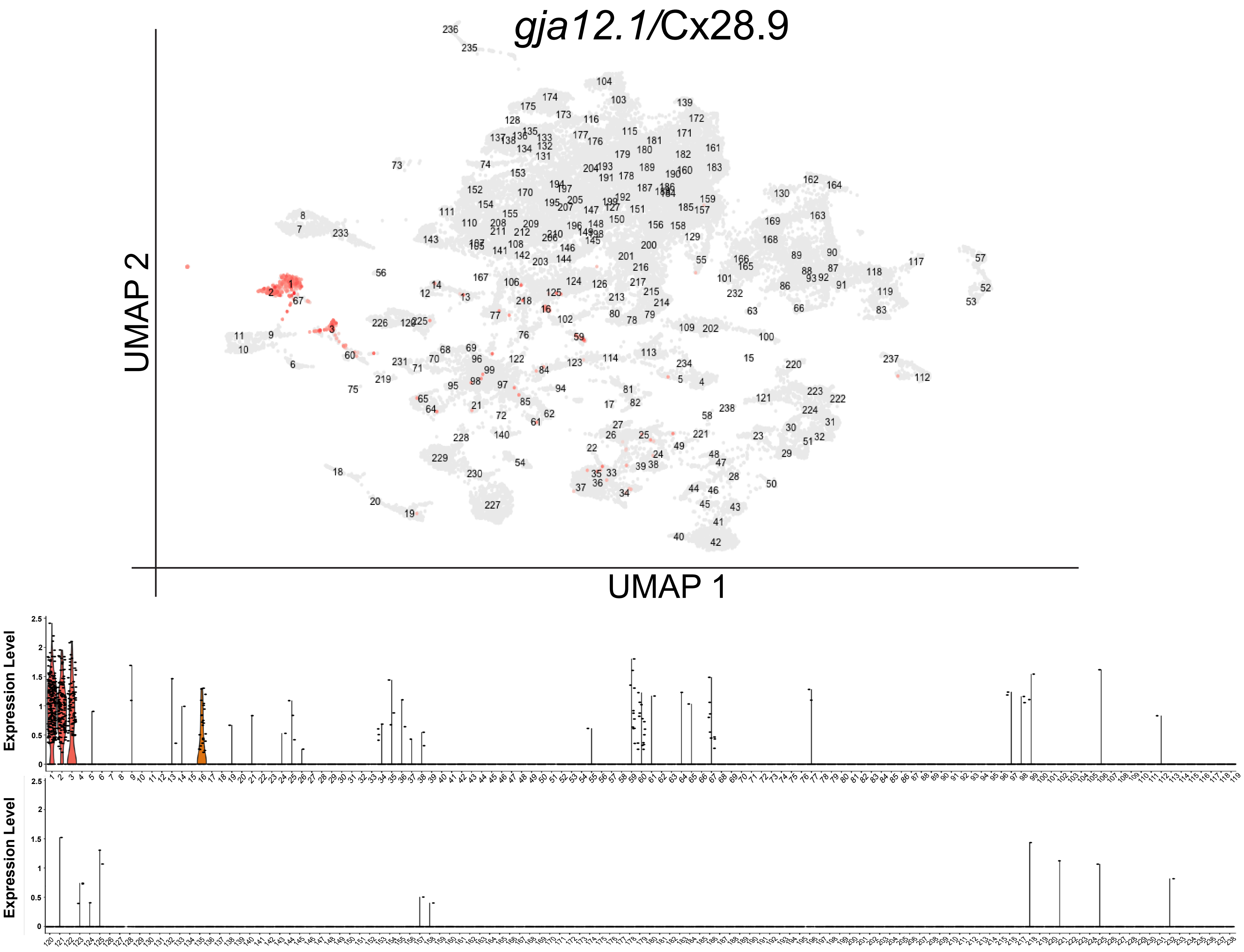

gja12.1/Cx28.9
UMAP 2
UMAP 1
Expression Level
Expression Level

P

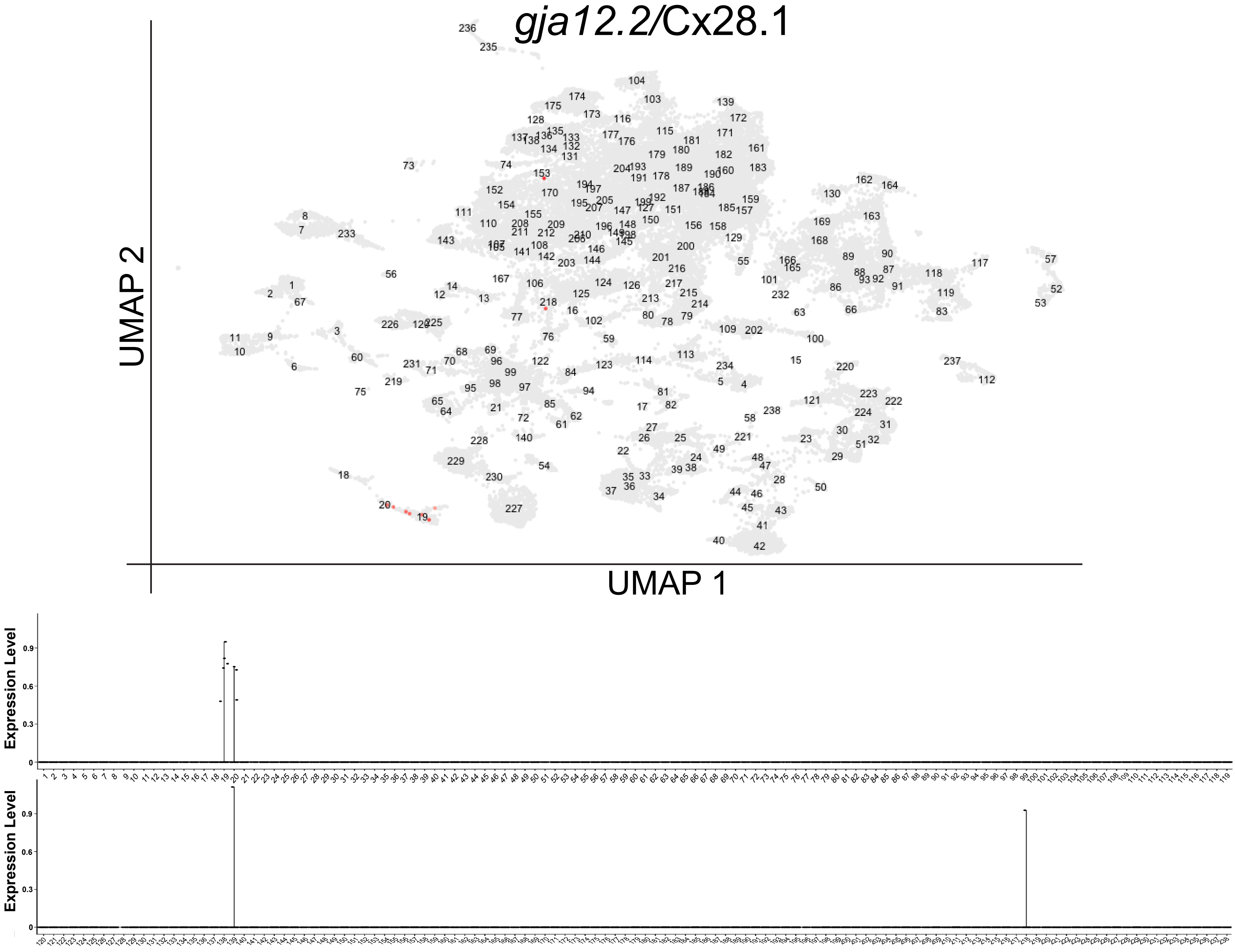
gja12.2/Cx28.1
UMAP 2
UMAP 1
Expression Level
0.9
0.6
0.3
0
Expression Level
0.9
0.6
0.3
0

Q

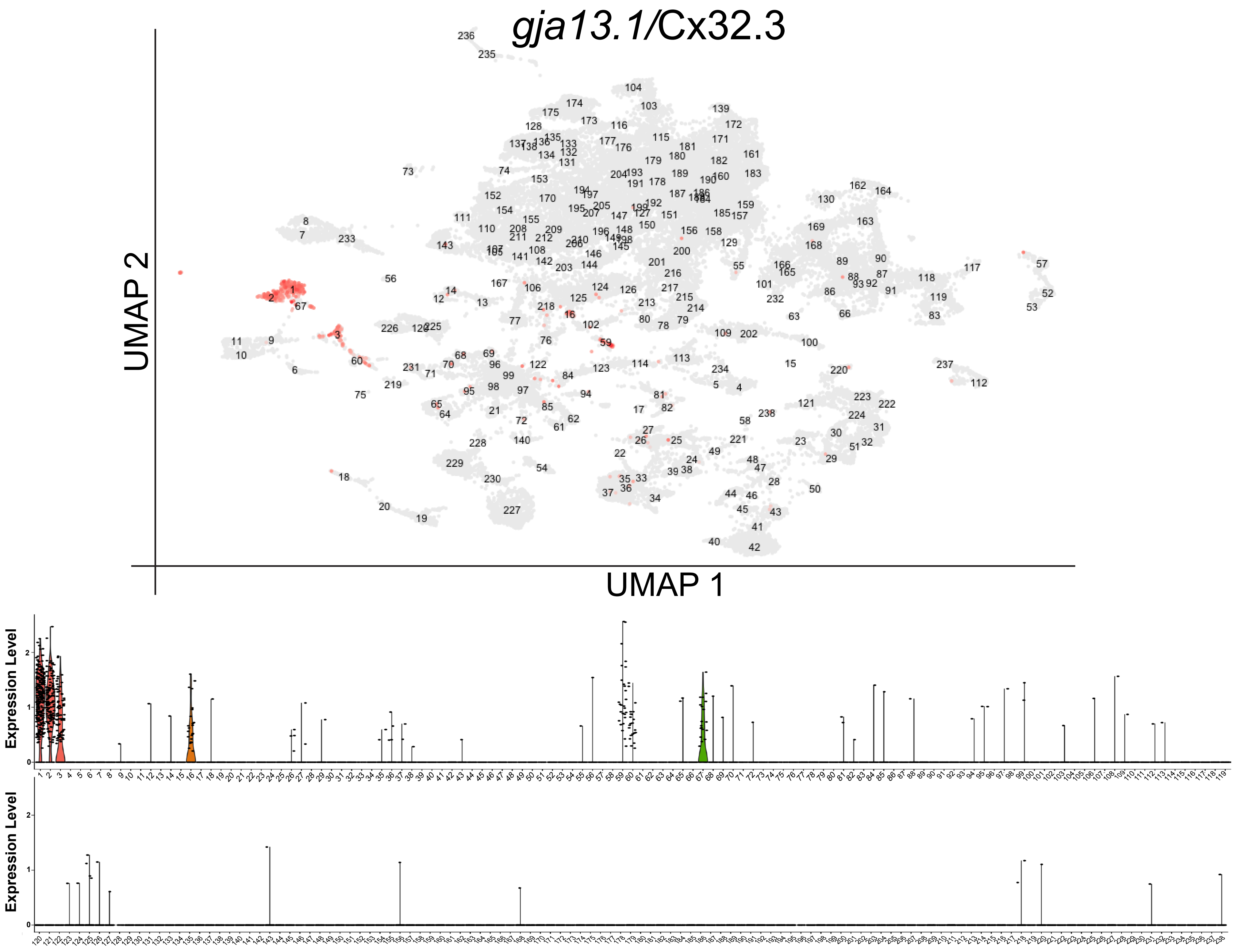
gja13.1/Cx32.3
UMAP 2
UMAP 1
Expression Level
Expression Level

R

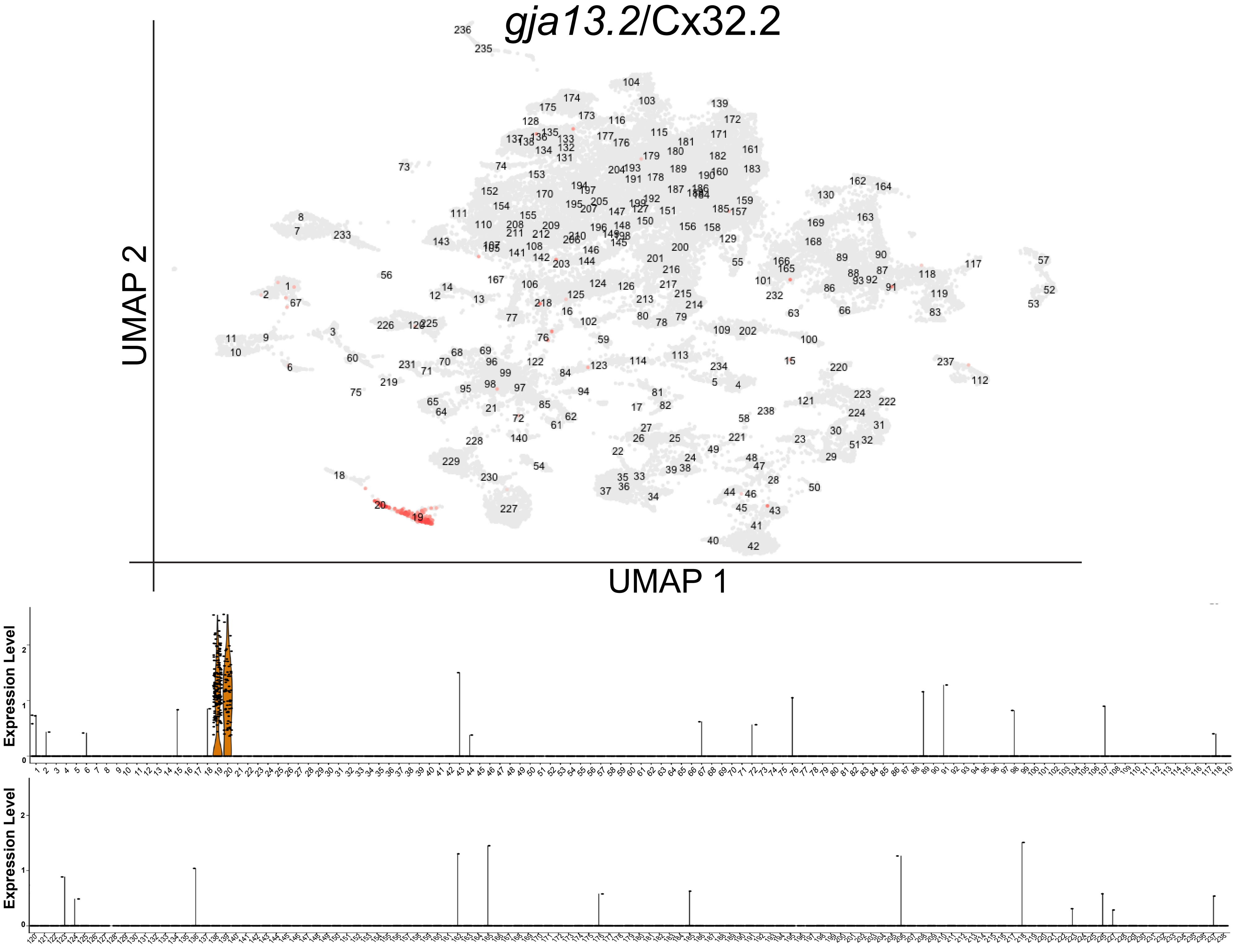

gja13.2/Cx32.2
UMAP 2
UMAP 1
Expression Level
0
1
2
Expression Level
0
1
2

S

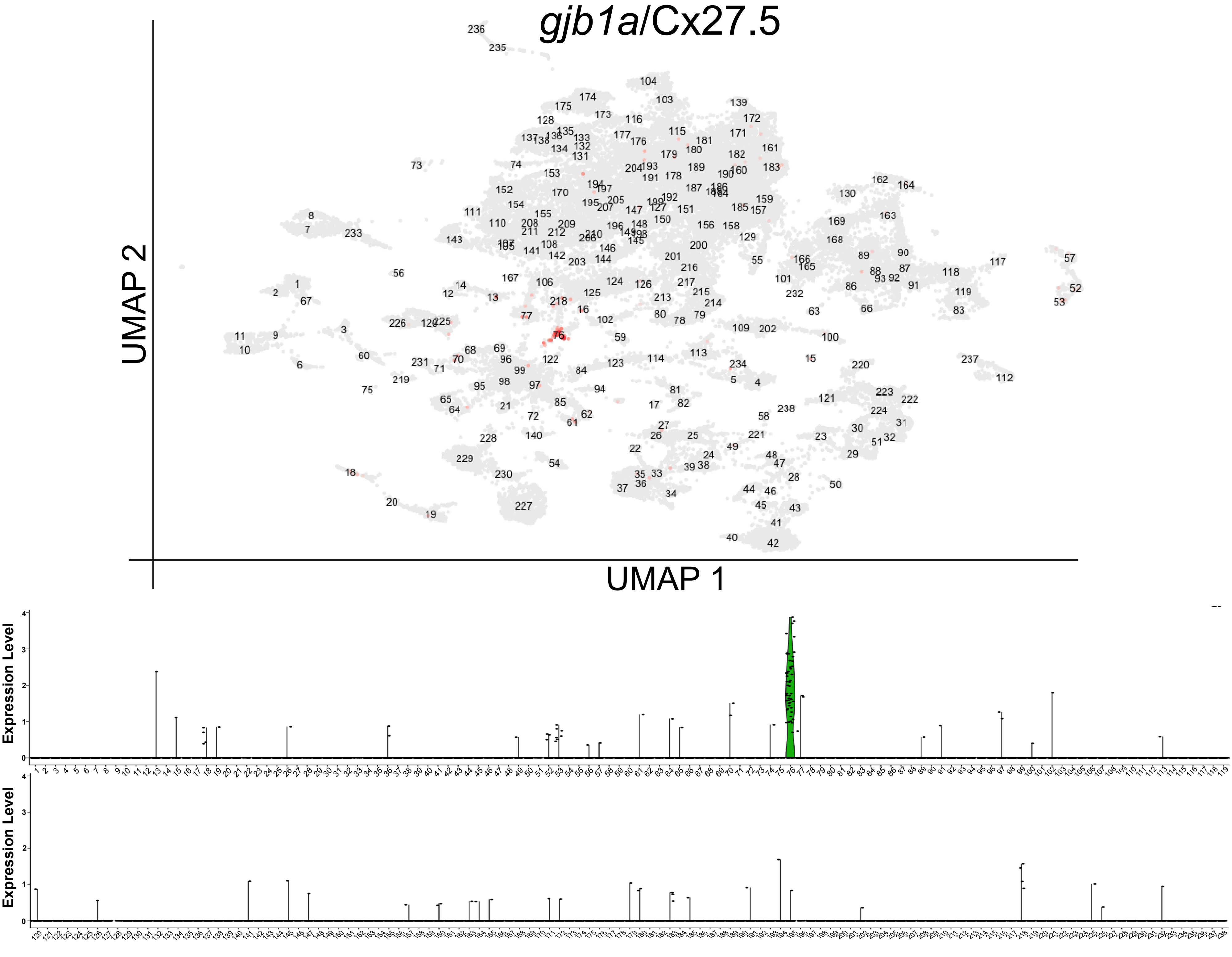
gjb1a/Cx27.5
UMAP 2
UMAP 1
Expression Level
Expression Level

T

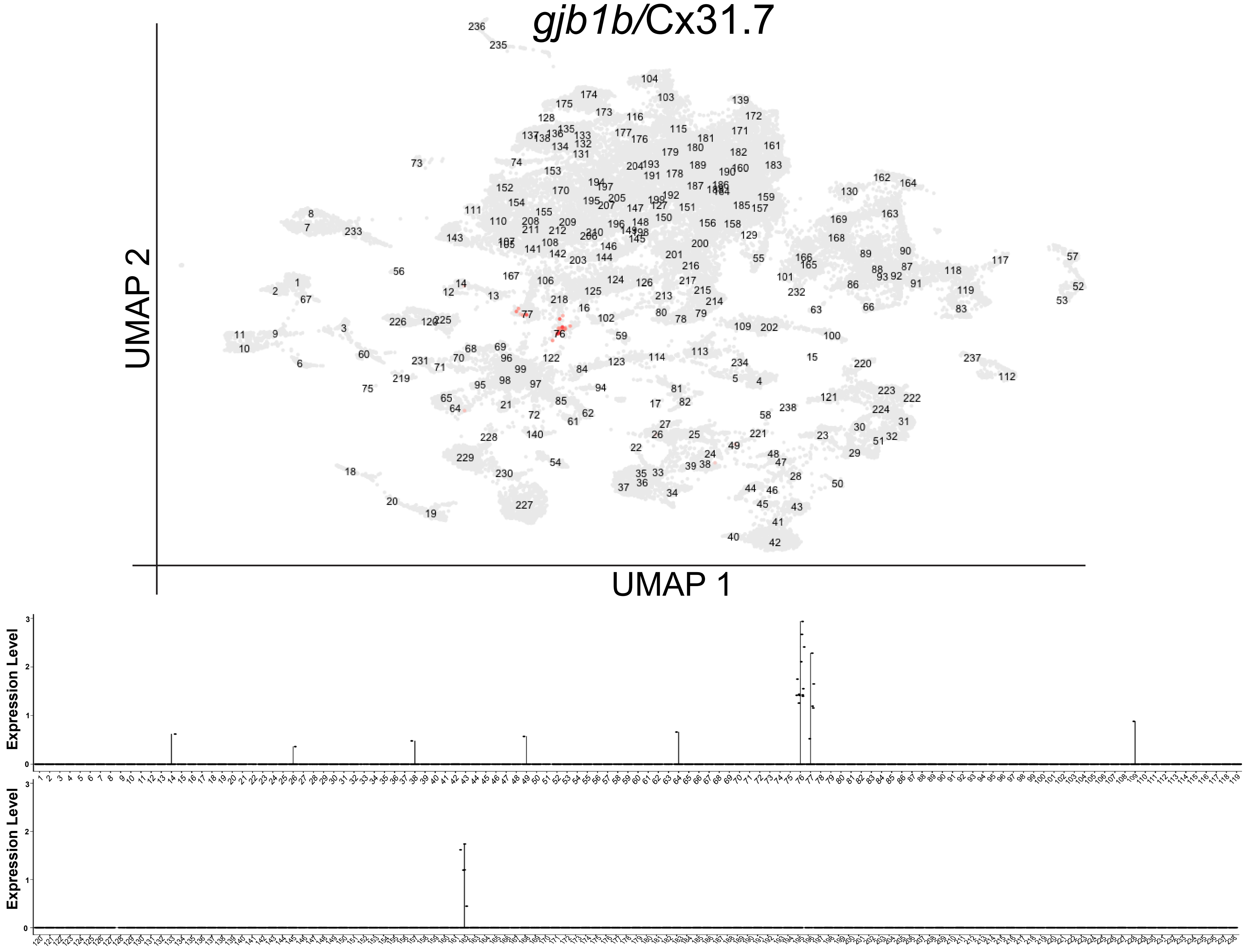

gjb1b/Cx31.7
UMAP 2
UMAP 1
Expression Level
Expression Level

U

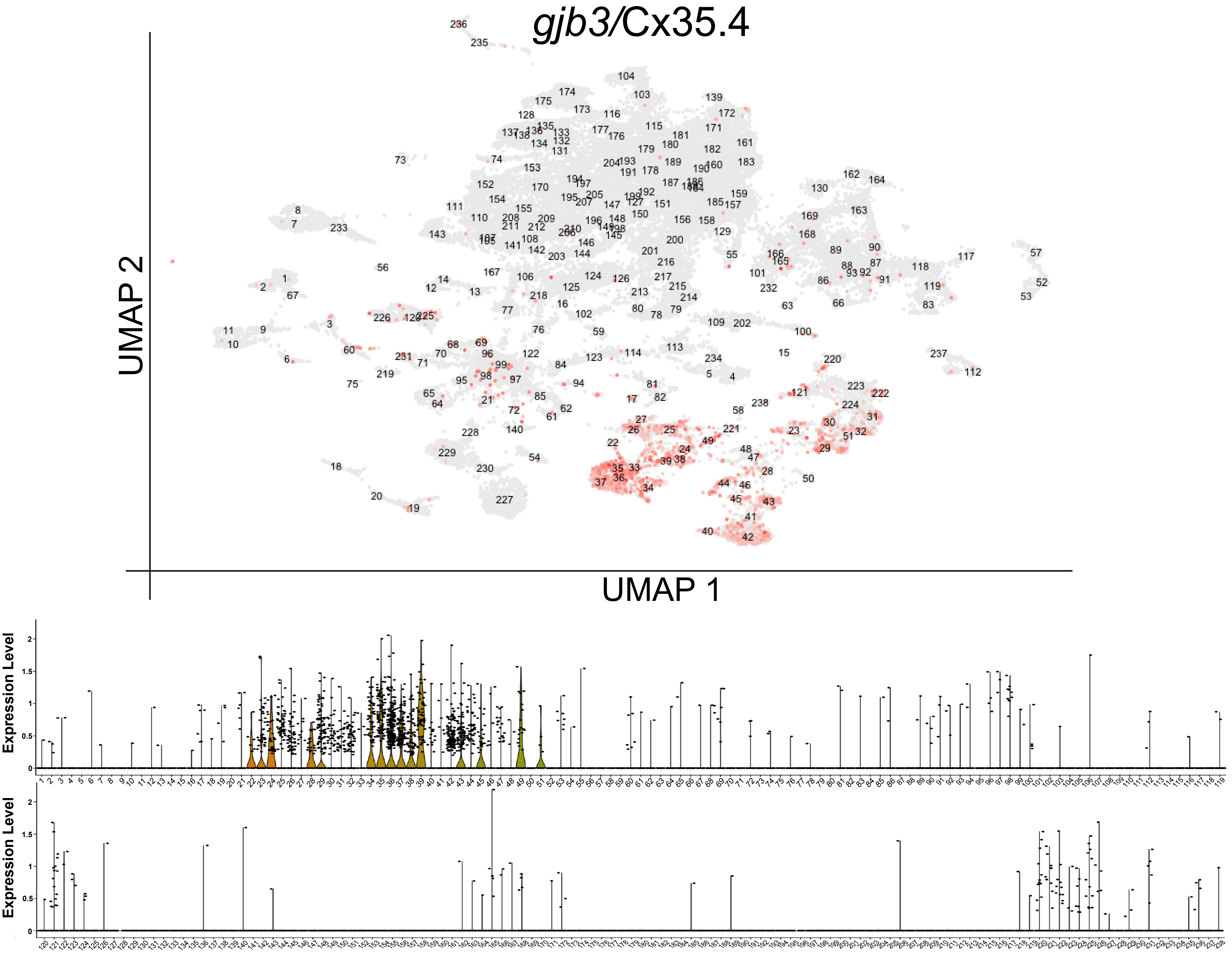

V

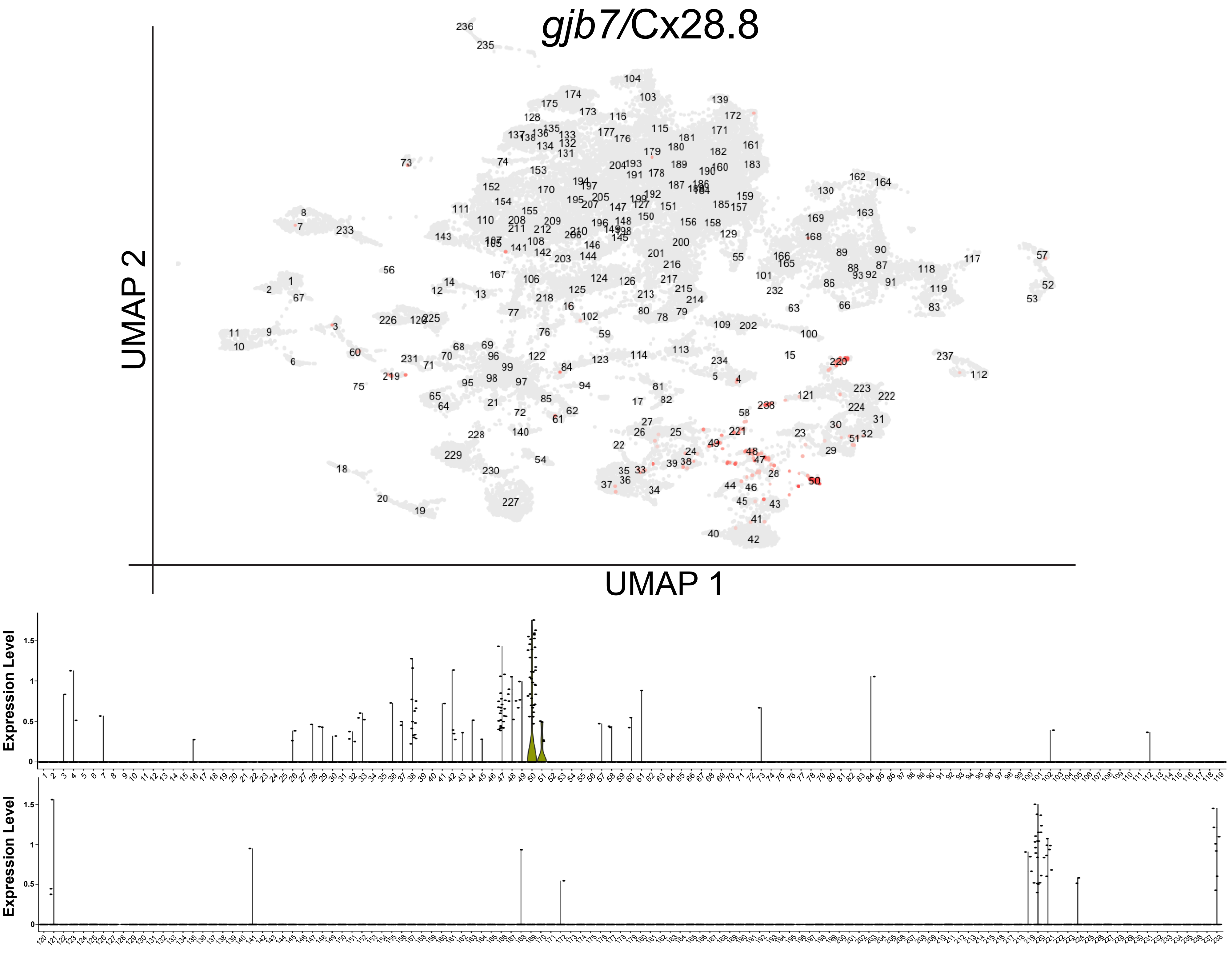

W

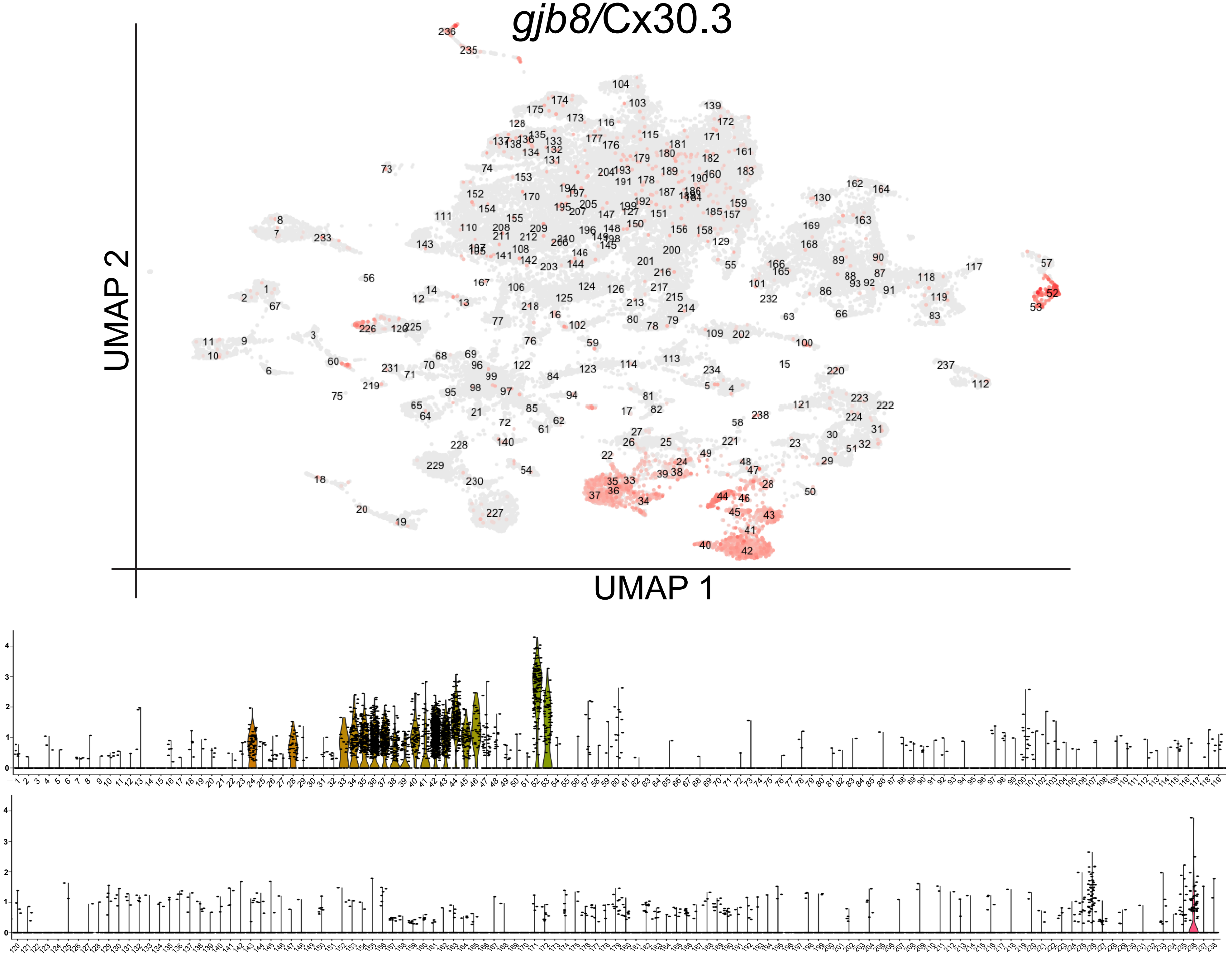

X

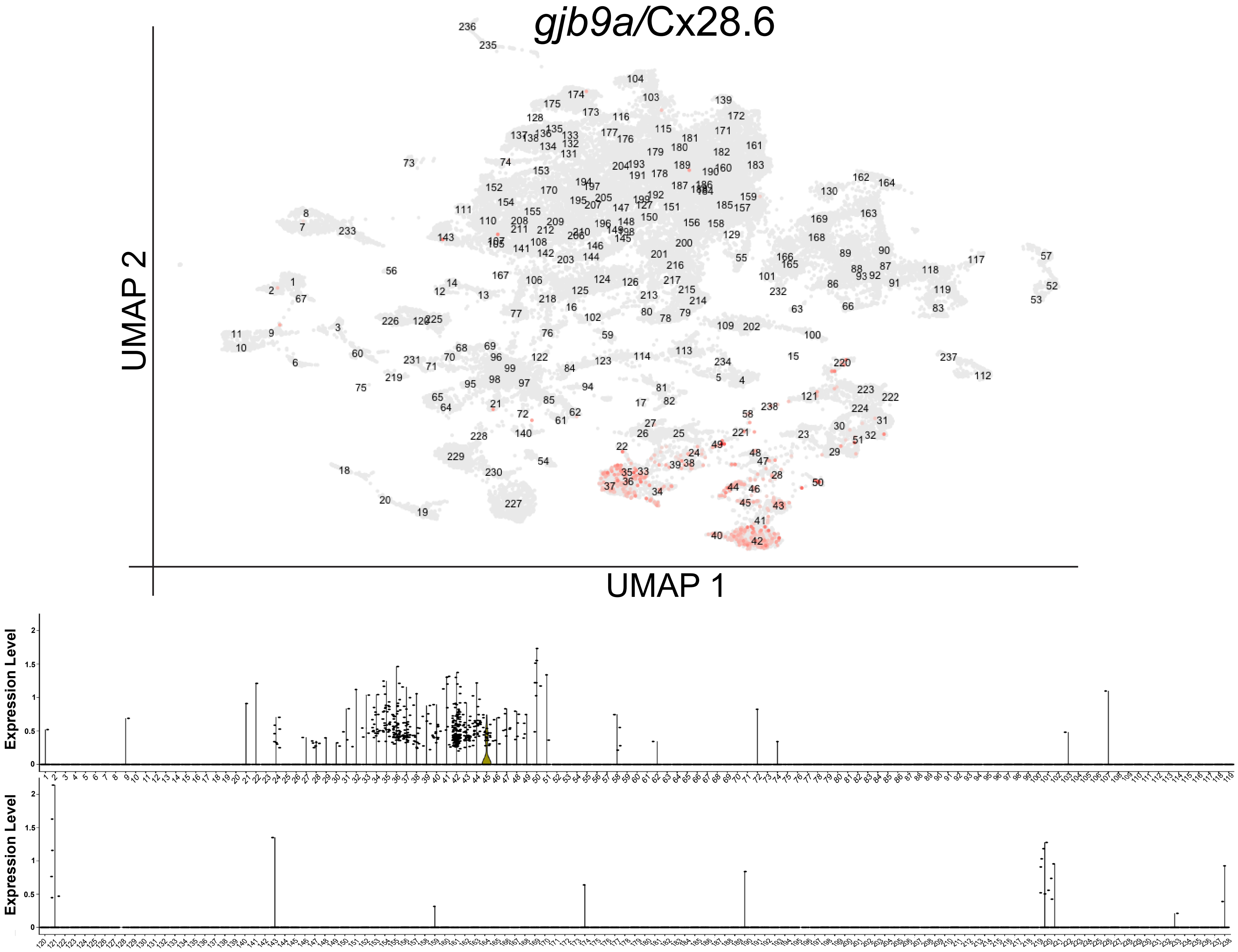

gjb9a/Cx28.6
UMAP 2
UMAP 1
Expression Level
Expression Level

Y

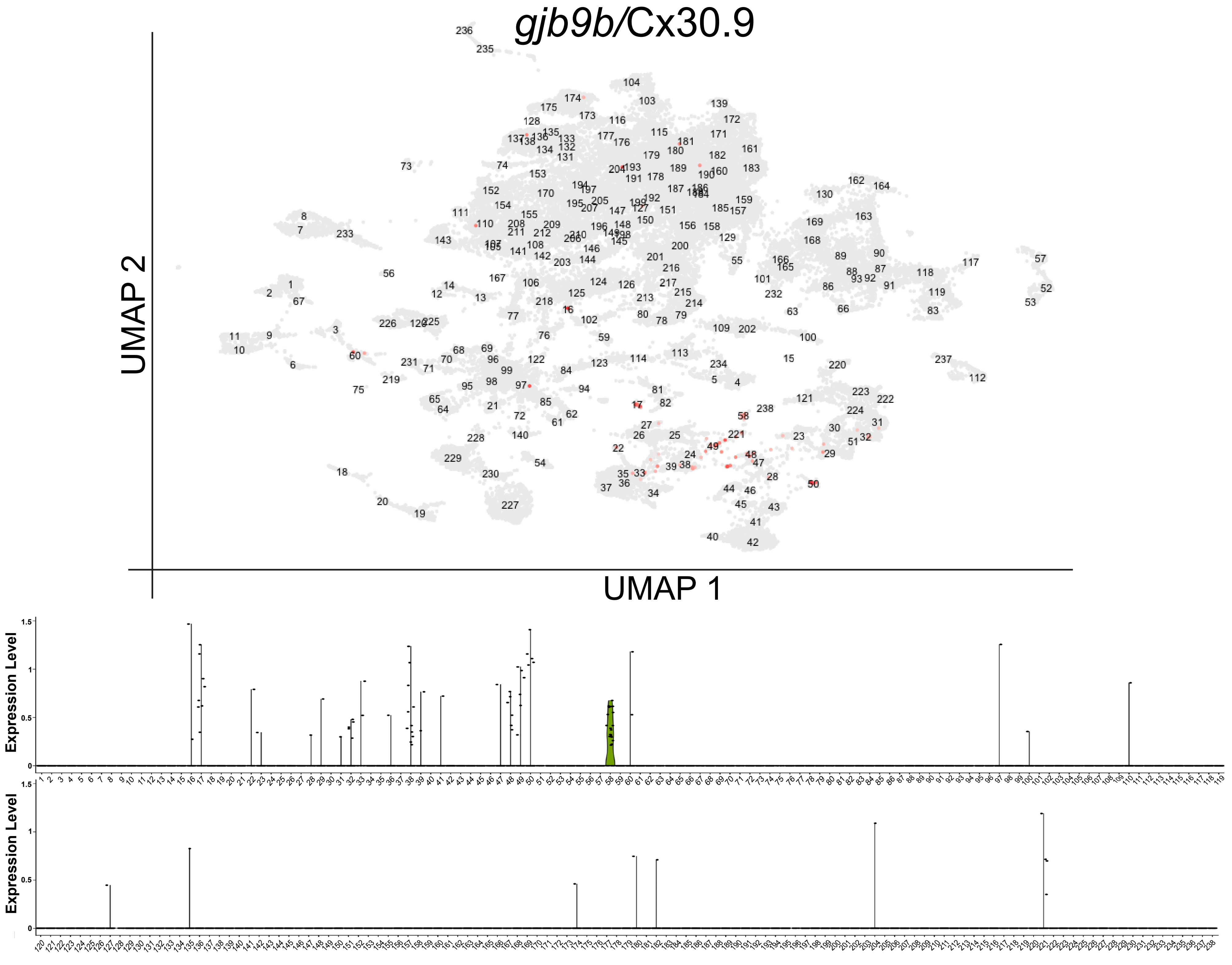
gjb9b/Cx30.9
UMAP 2
UMAP 1
Expression Level
Expression Level

Z

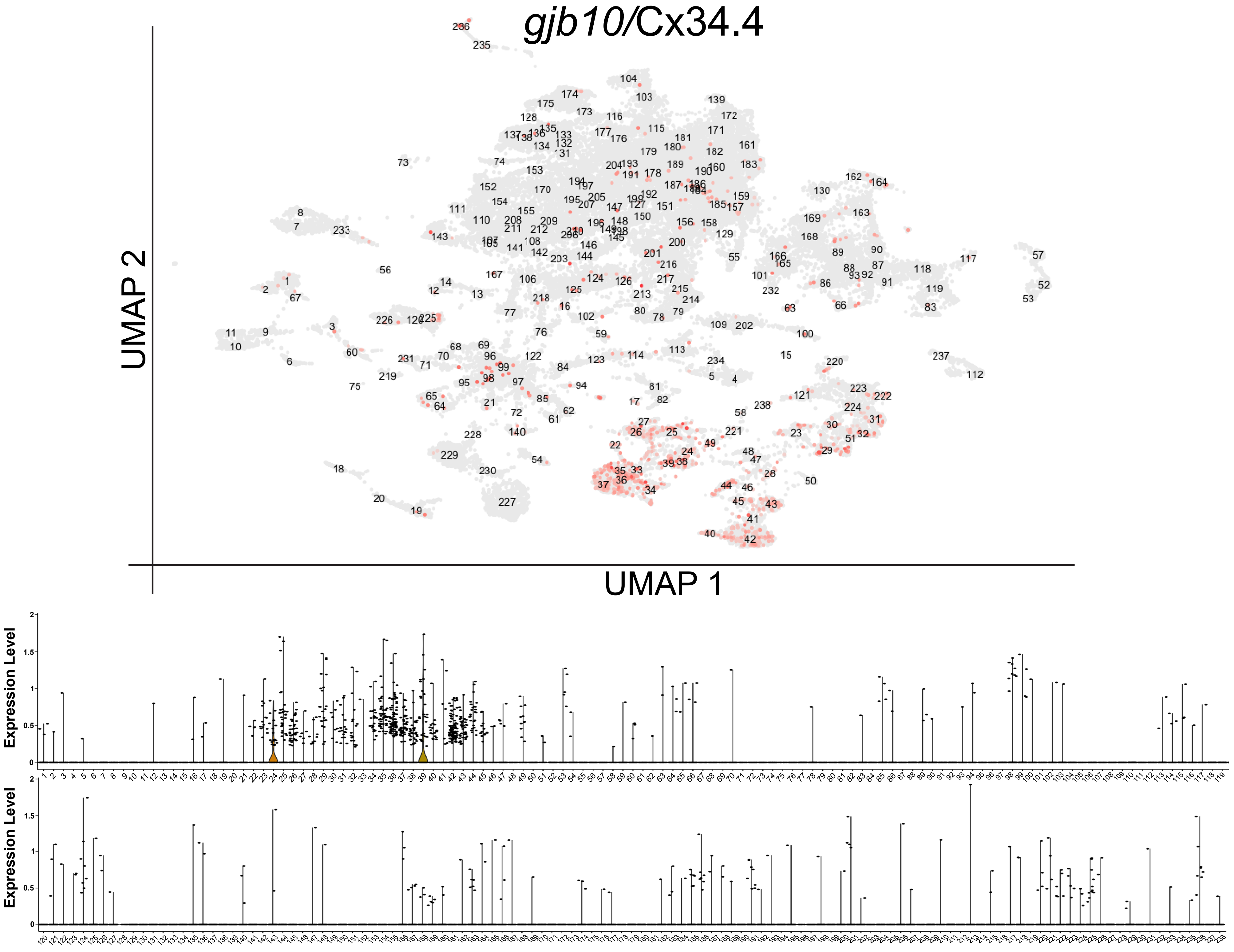
gjb10/Cx34.4
UMAP 2
UMAP 1
Expression Level
Expression Level

AA

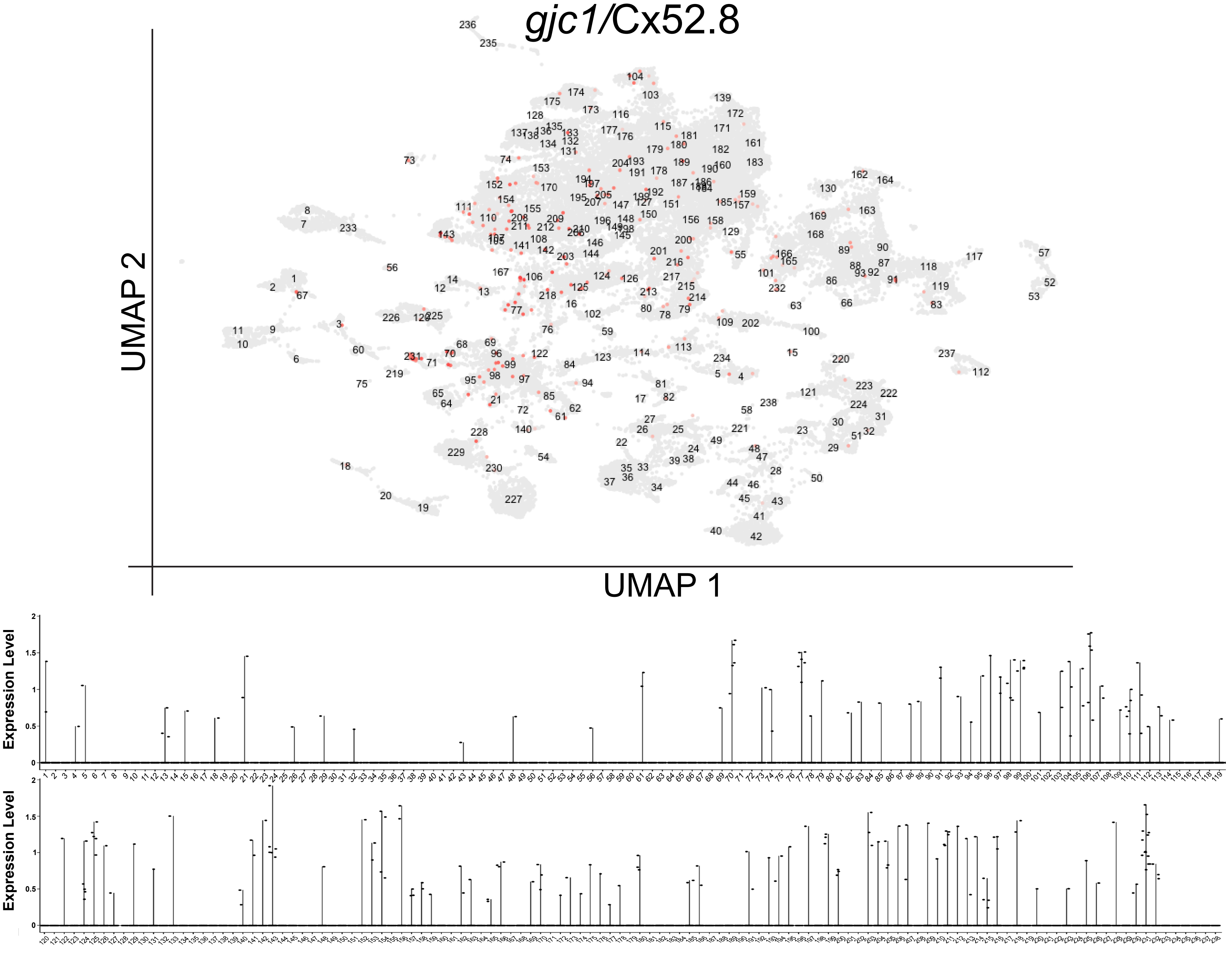

BB

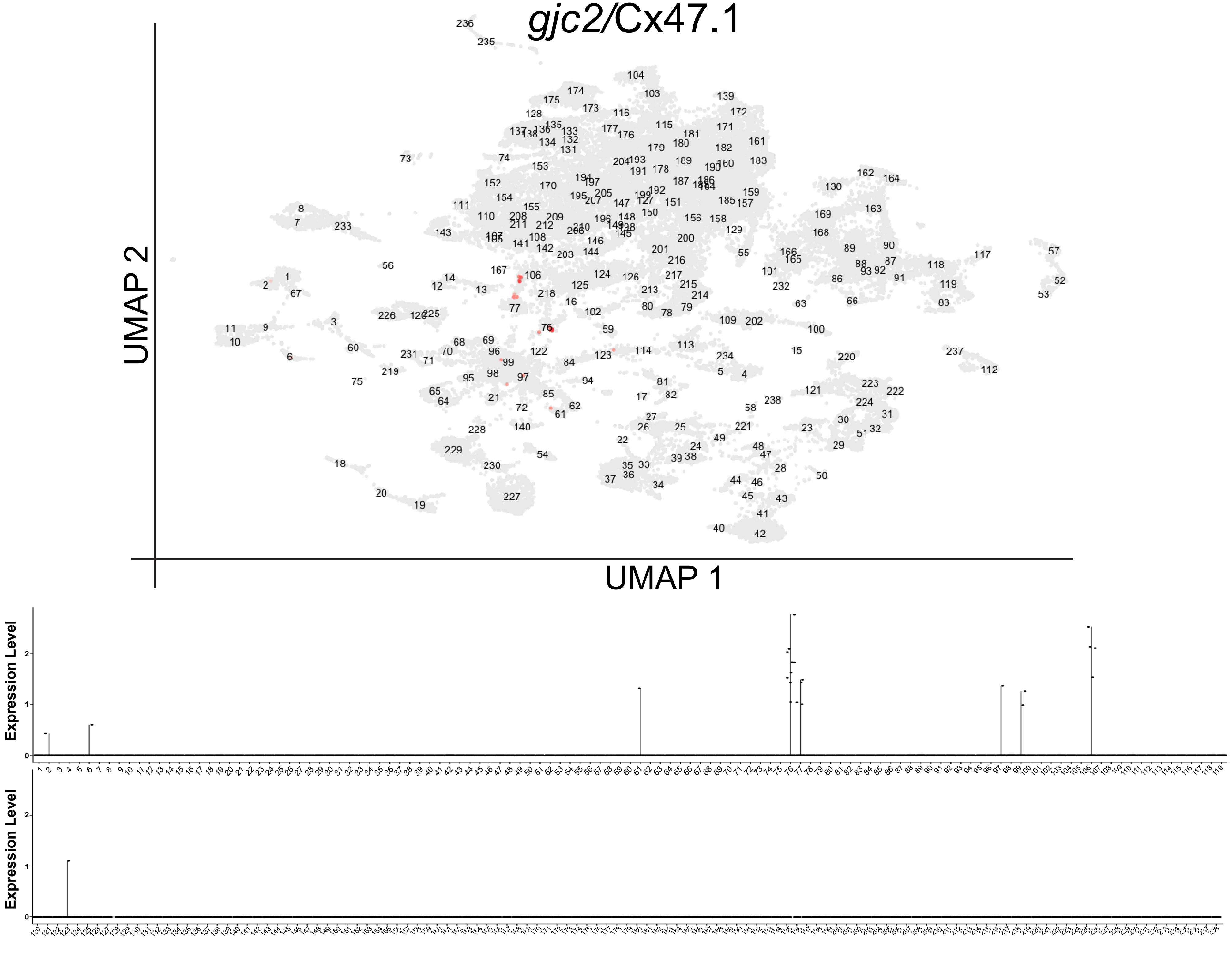

CC

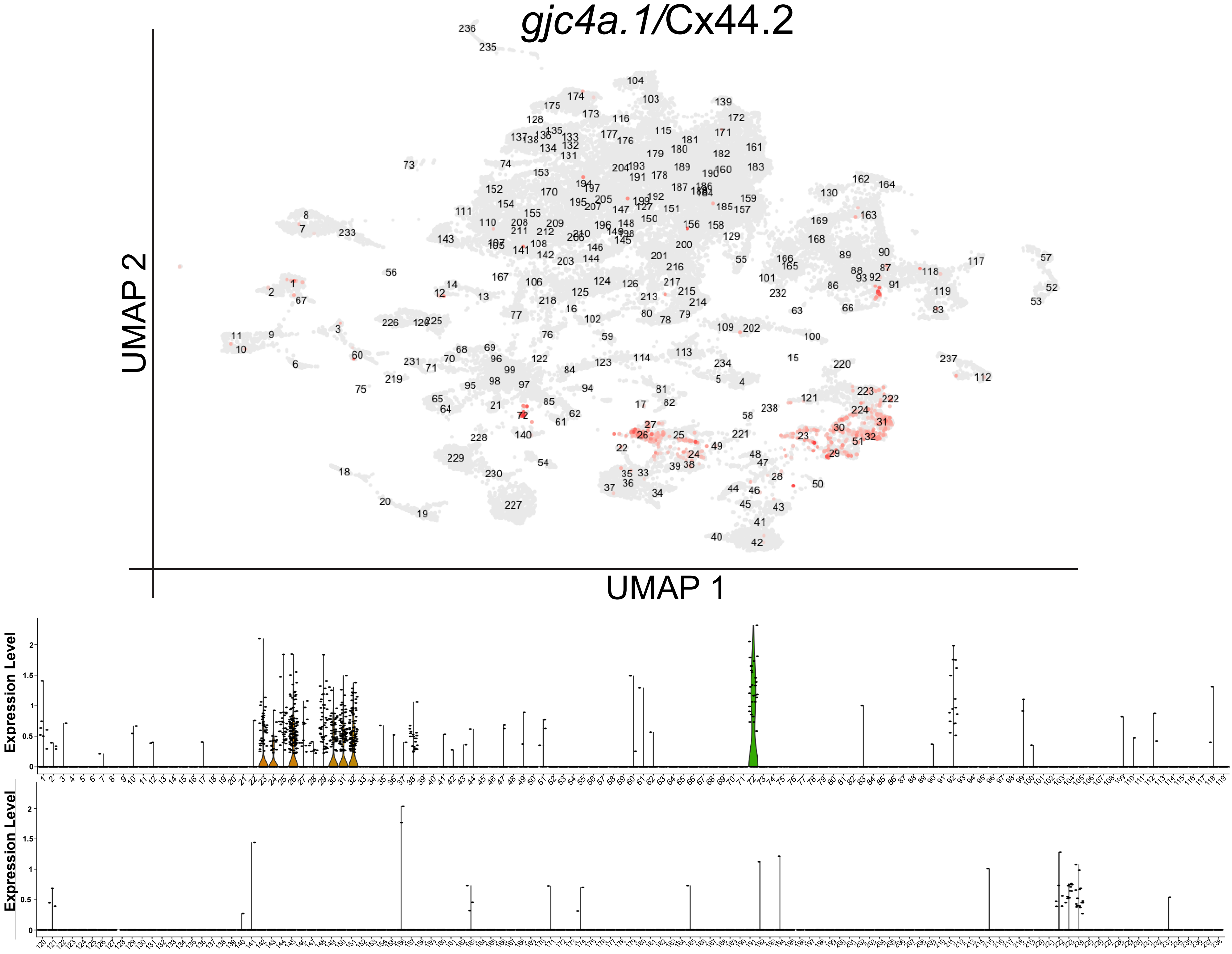

gjc4a.1/Cx44.2
UMAP 1
UMAP 2
Expression Level
Expression Level

DD

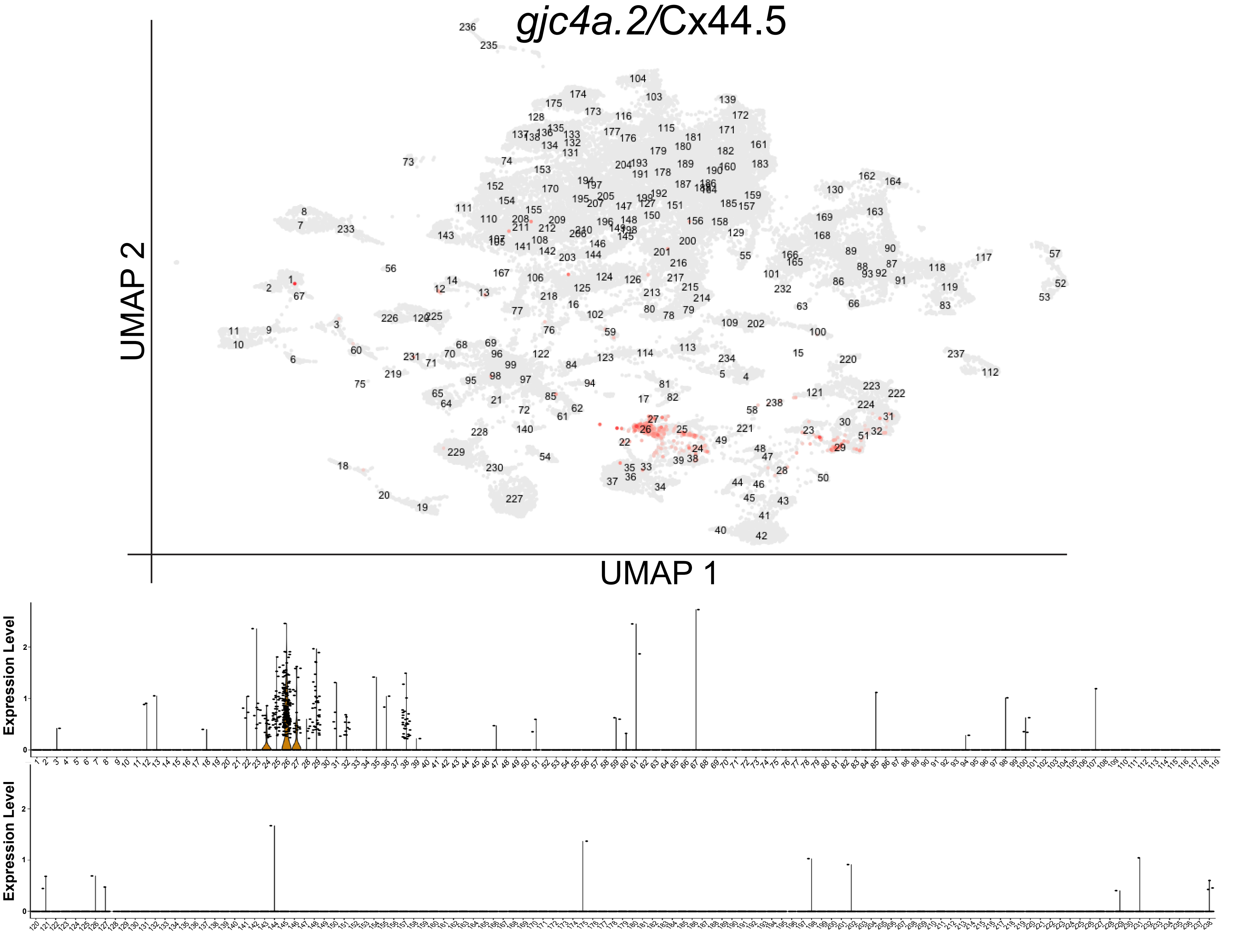

gjc4a.2/Cx44.5
UMAP 2
UMAP 1
Expression Level
Expression Level

EE

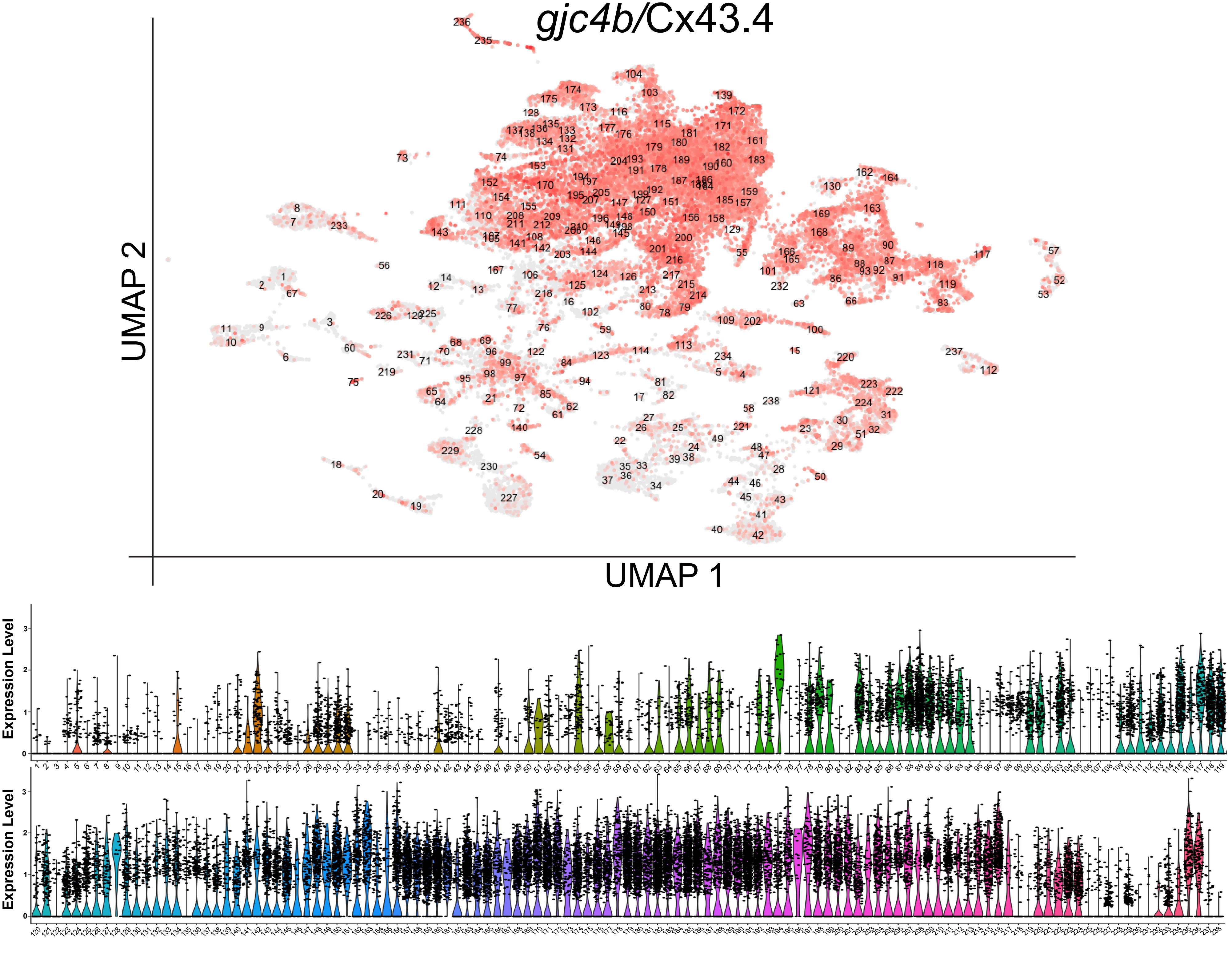

gjc4b/Cx43.4
UMAP 2
UMAP 1
Expression Level
Expression Level
0
1
2
3

FF

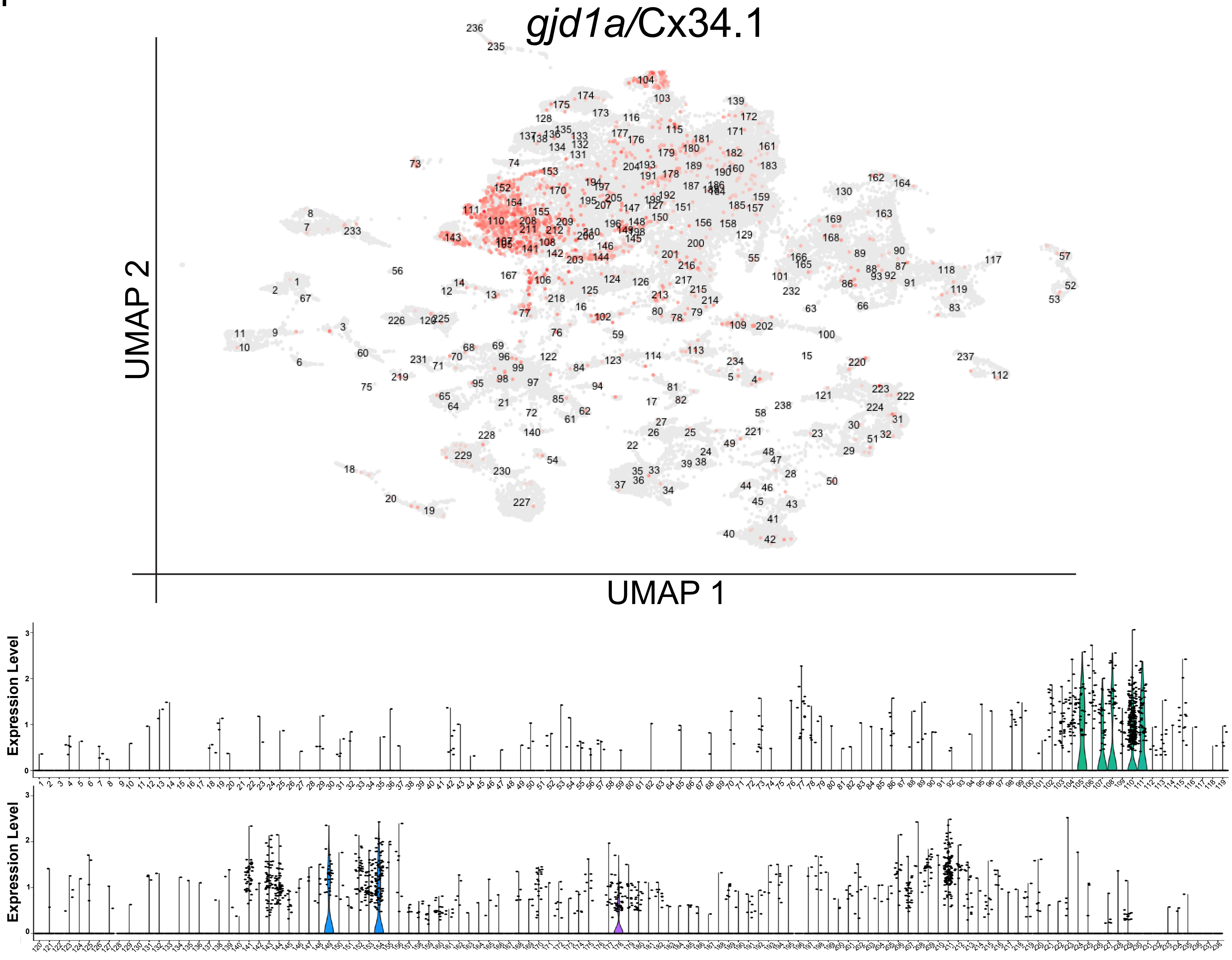

gjd1a/Cx34.1
UMAP 2
UMAP 1
Expression Level
Expression Level

GG

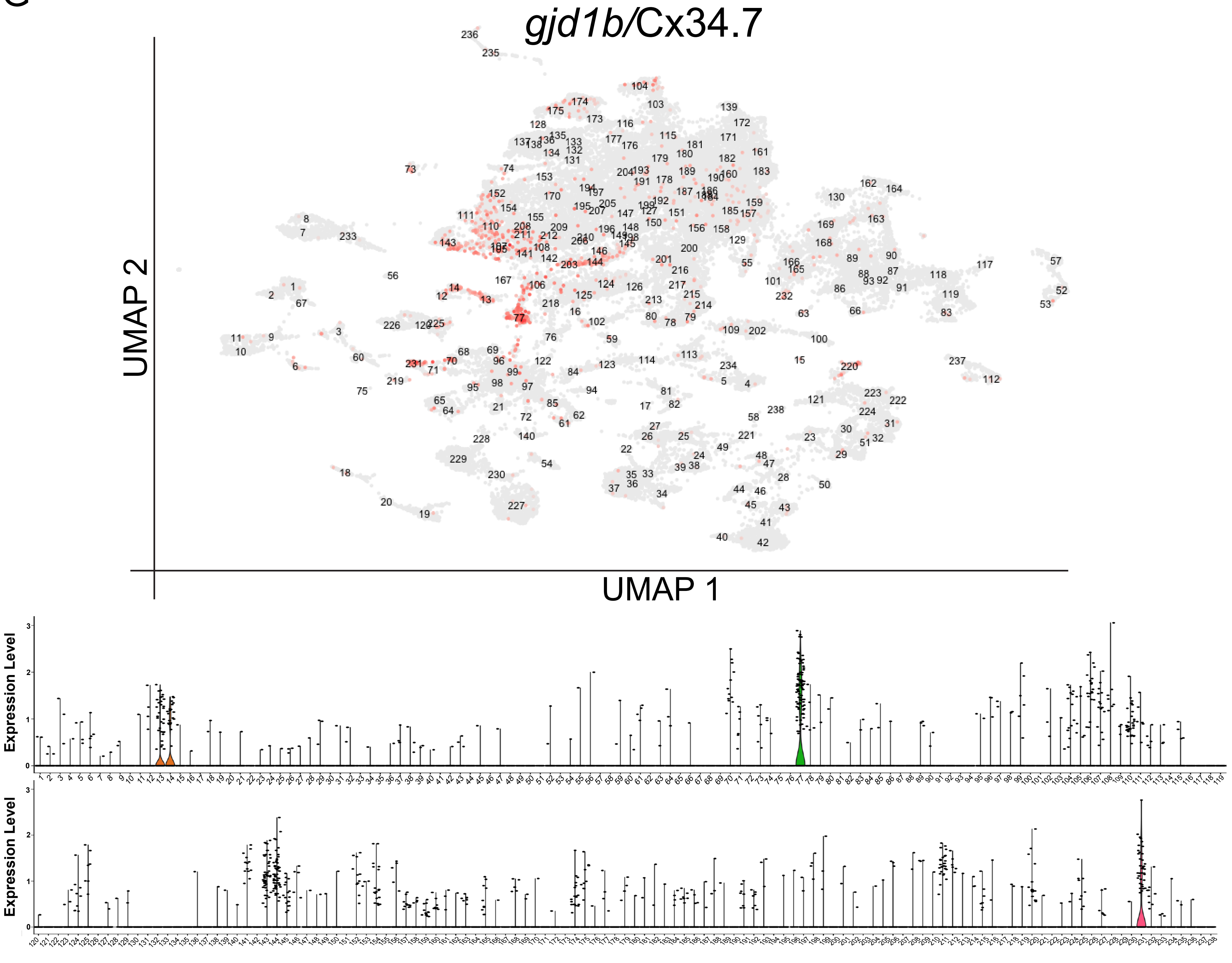
gjd1b/Cx34.7
UMAP 2
UMAP 1
Expression Level
Expression Level

HH

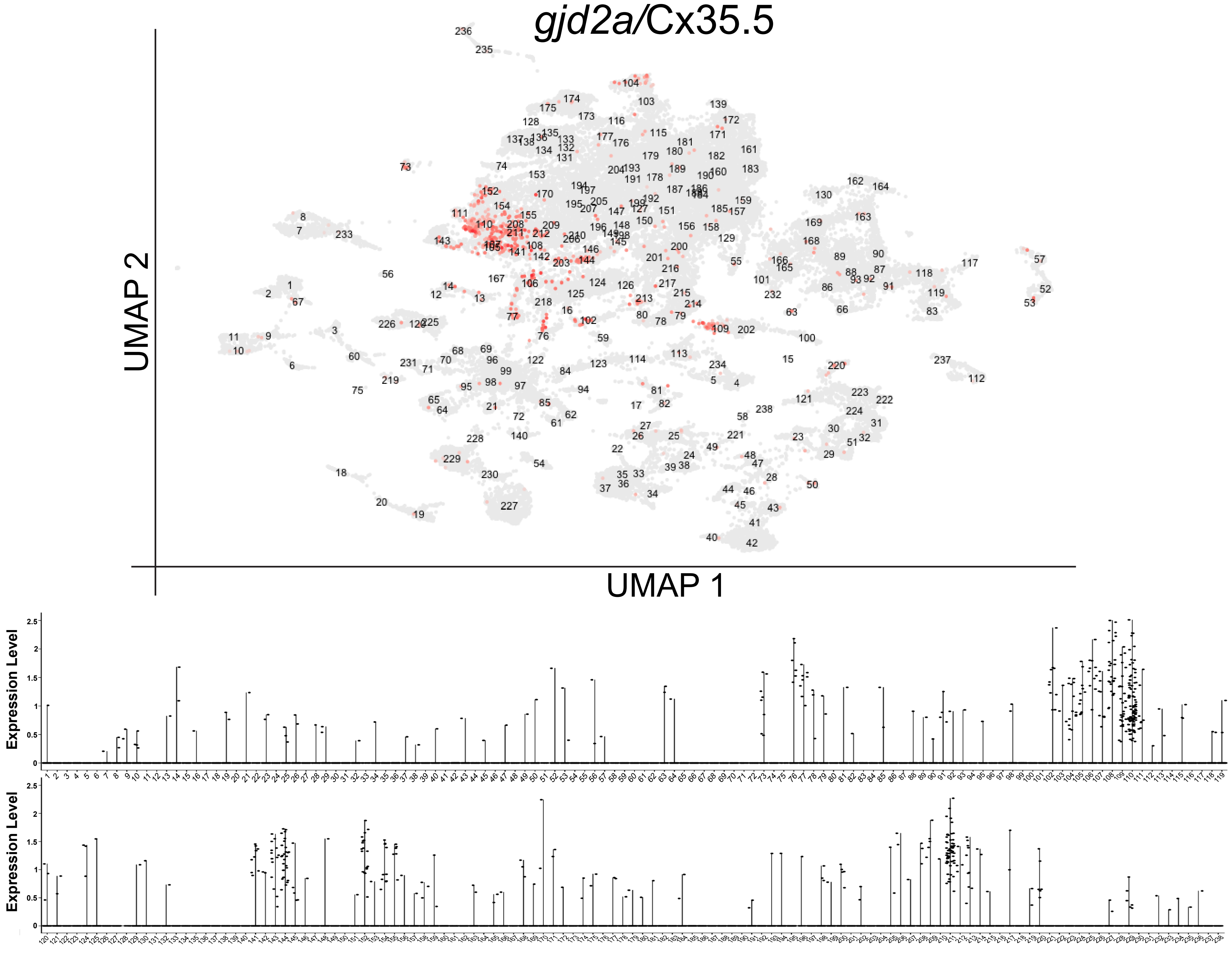

gjd2a/Cx35.5
UMAP 2
UMAP 1
Expression Level
Expression Level

II

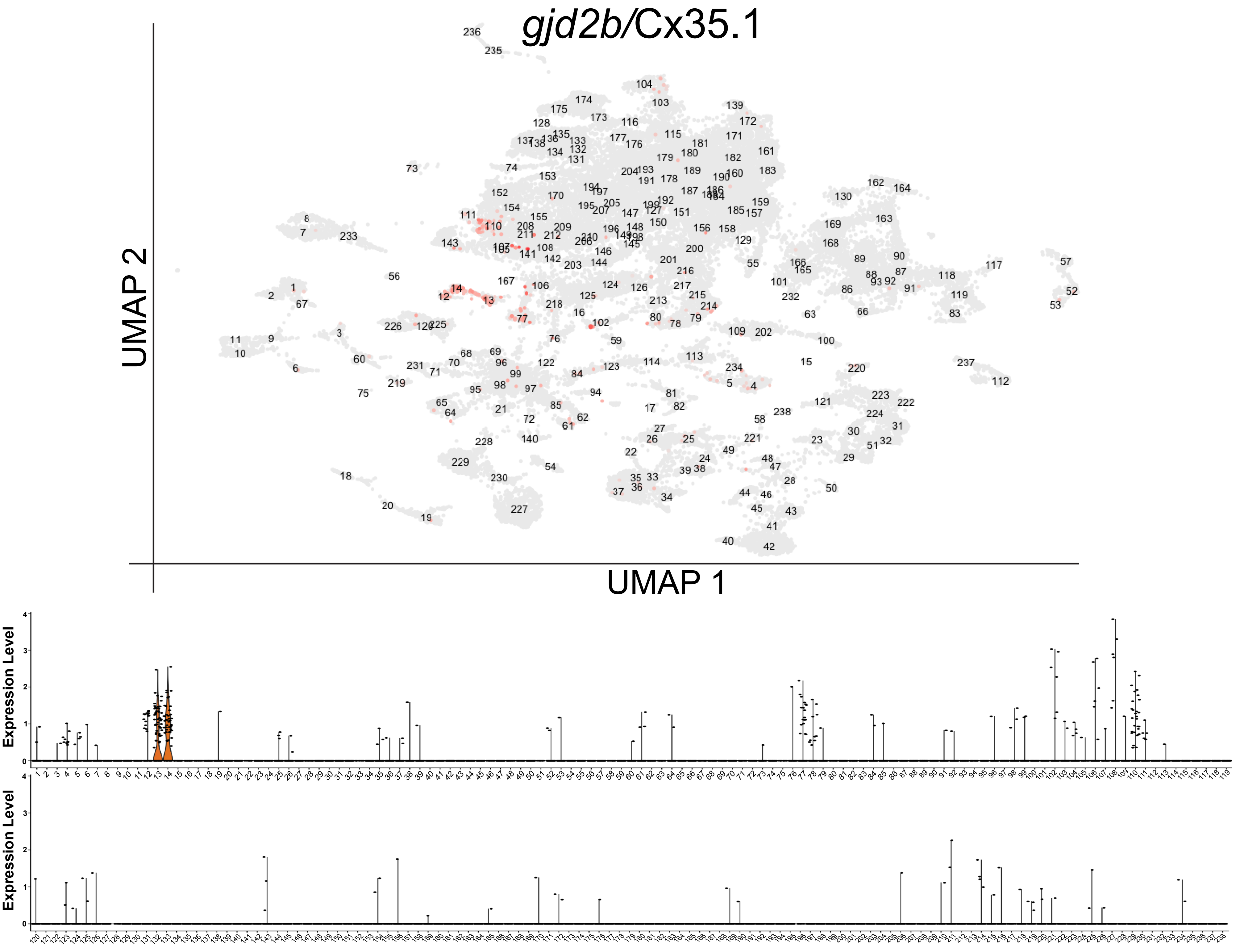

gjd2b/Cx35.1
UMAP 2
UMAP 1
Expression Level
Expression Level

JJ

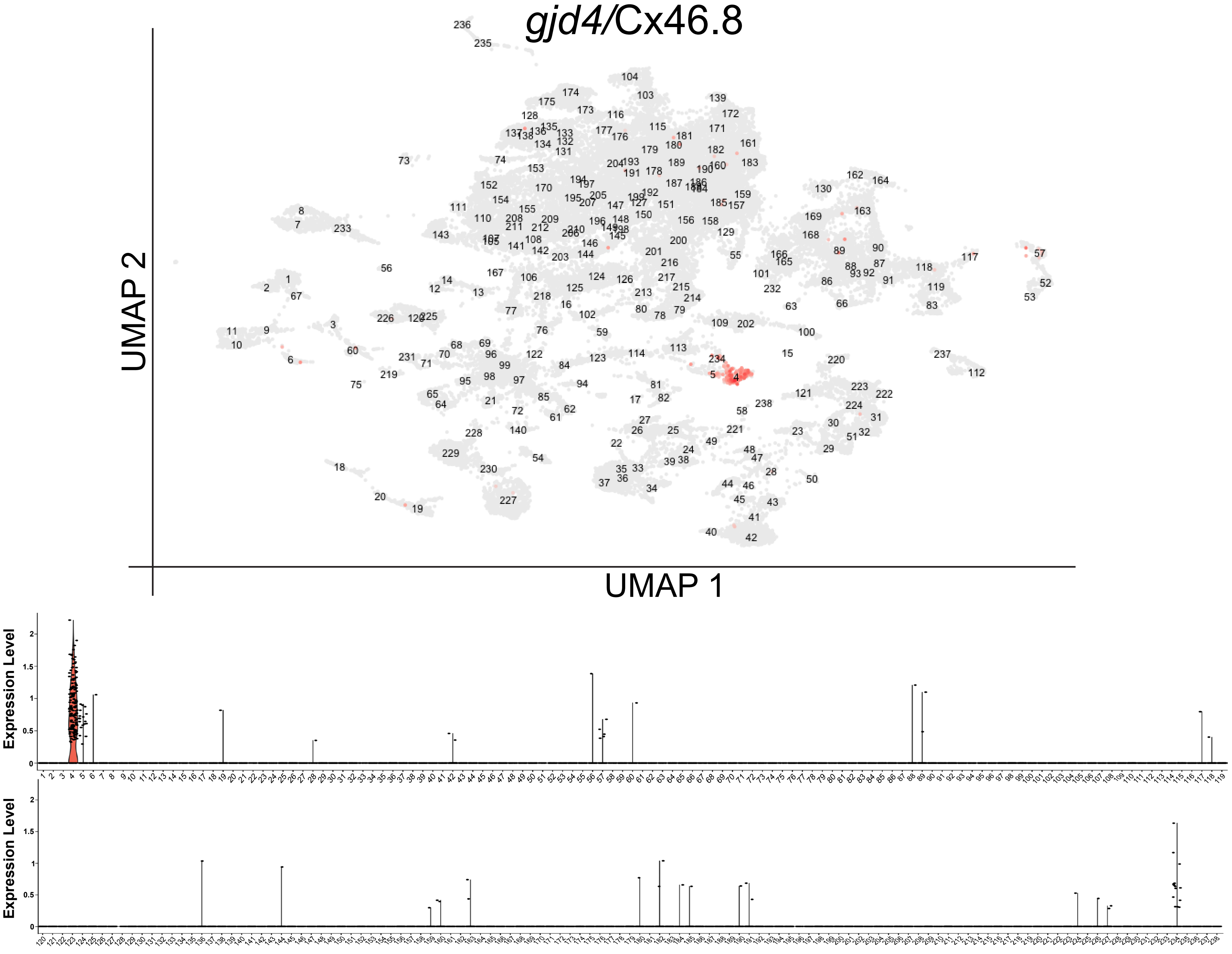

KK

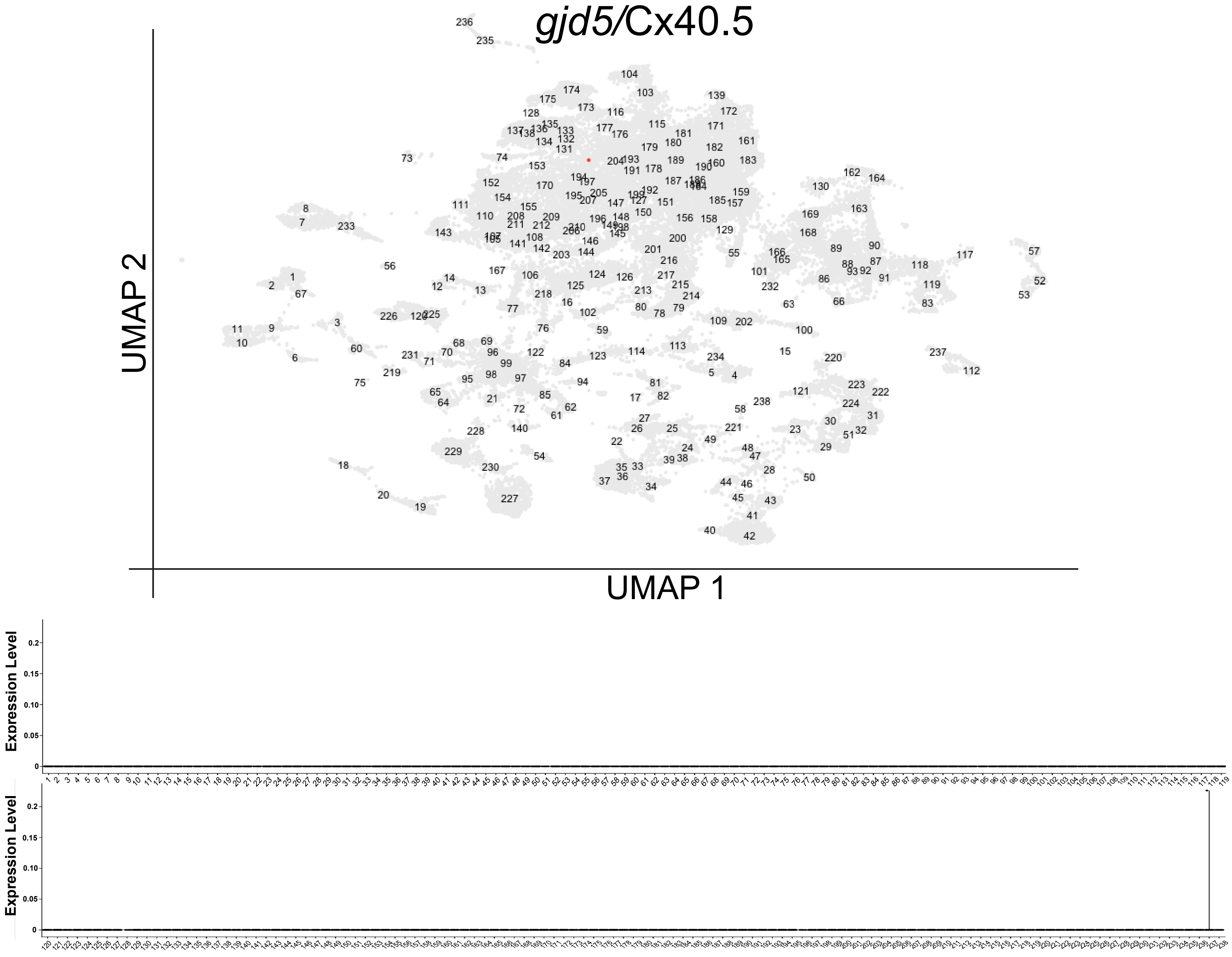

LL

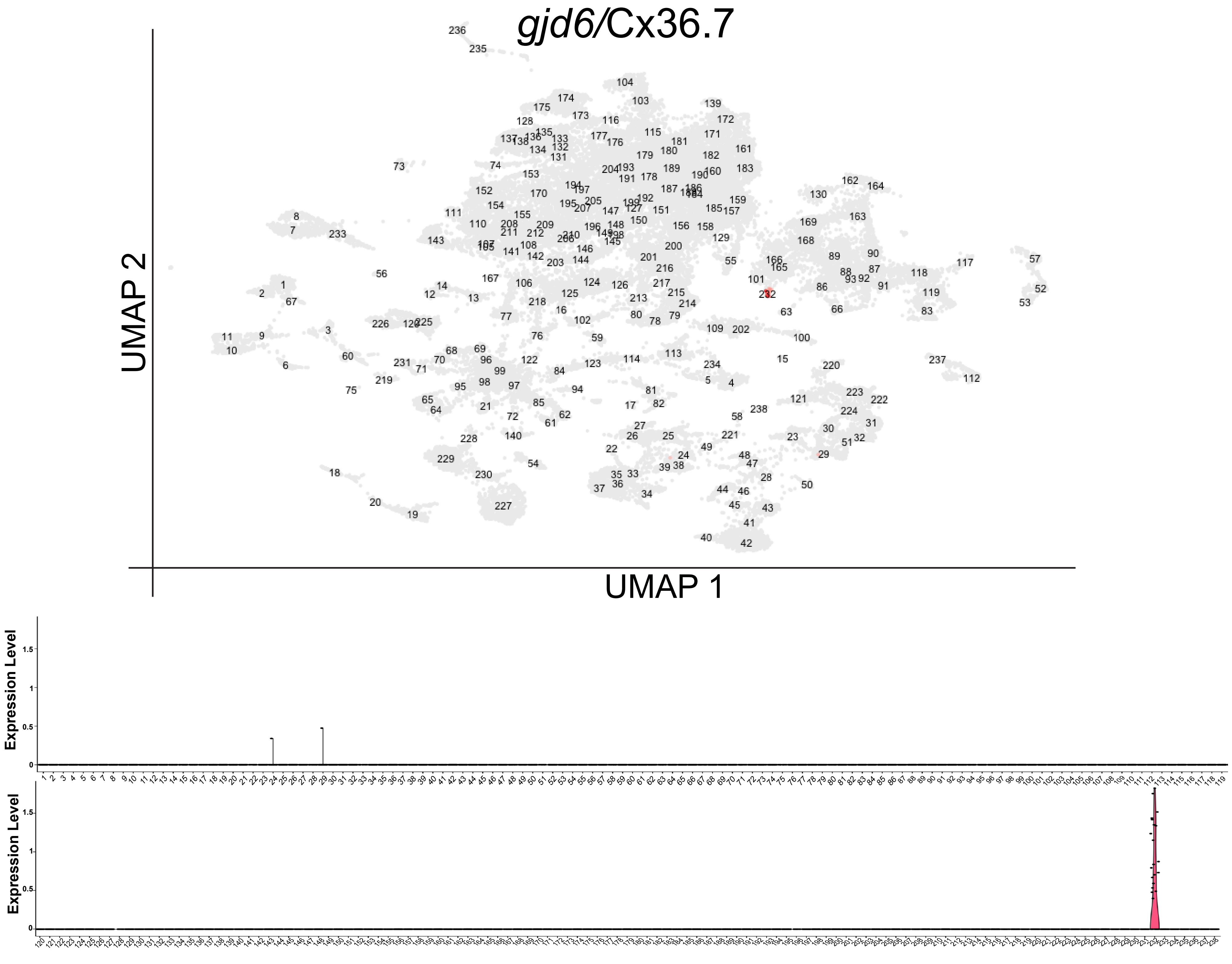

MM

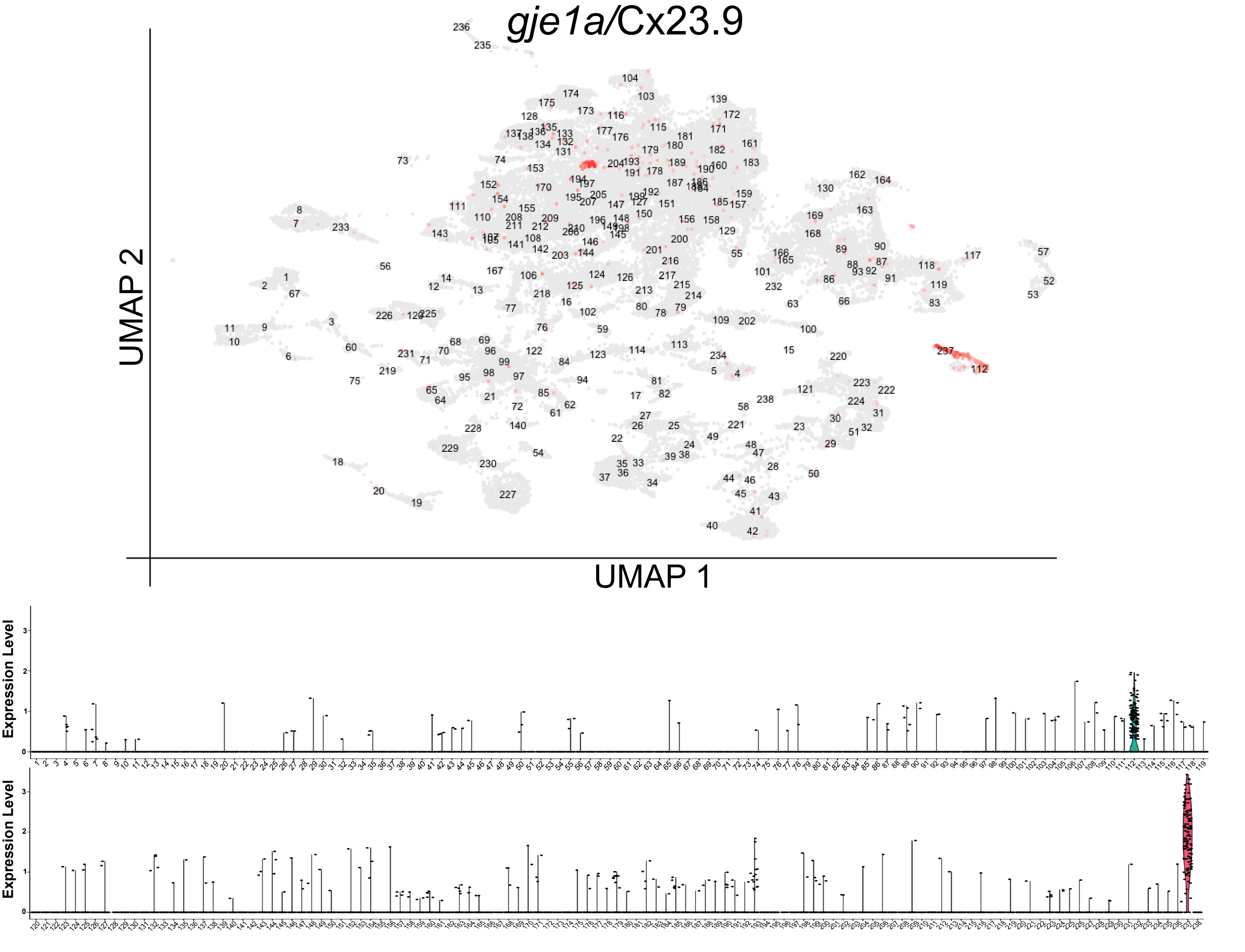

gje1a/Cx23.9
UMAP 2
UMAP 1
Expression Level
Expression Level

NN

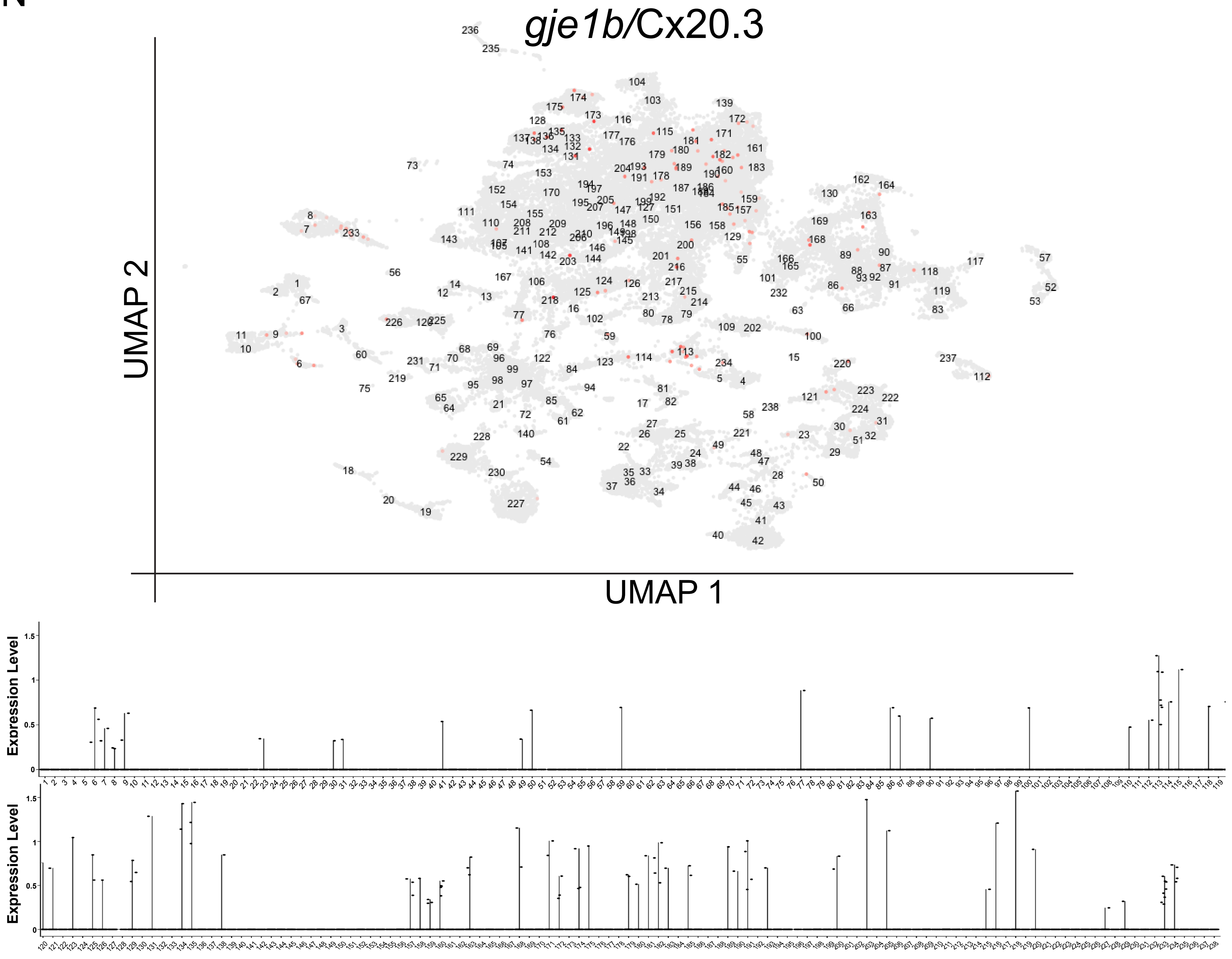

gje1b/Cx20.3
UMAP 2
UMAP 1
Expression Level
Expression Level

OO

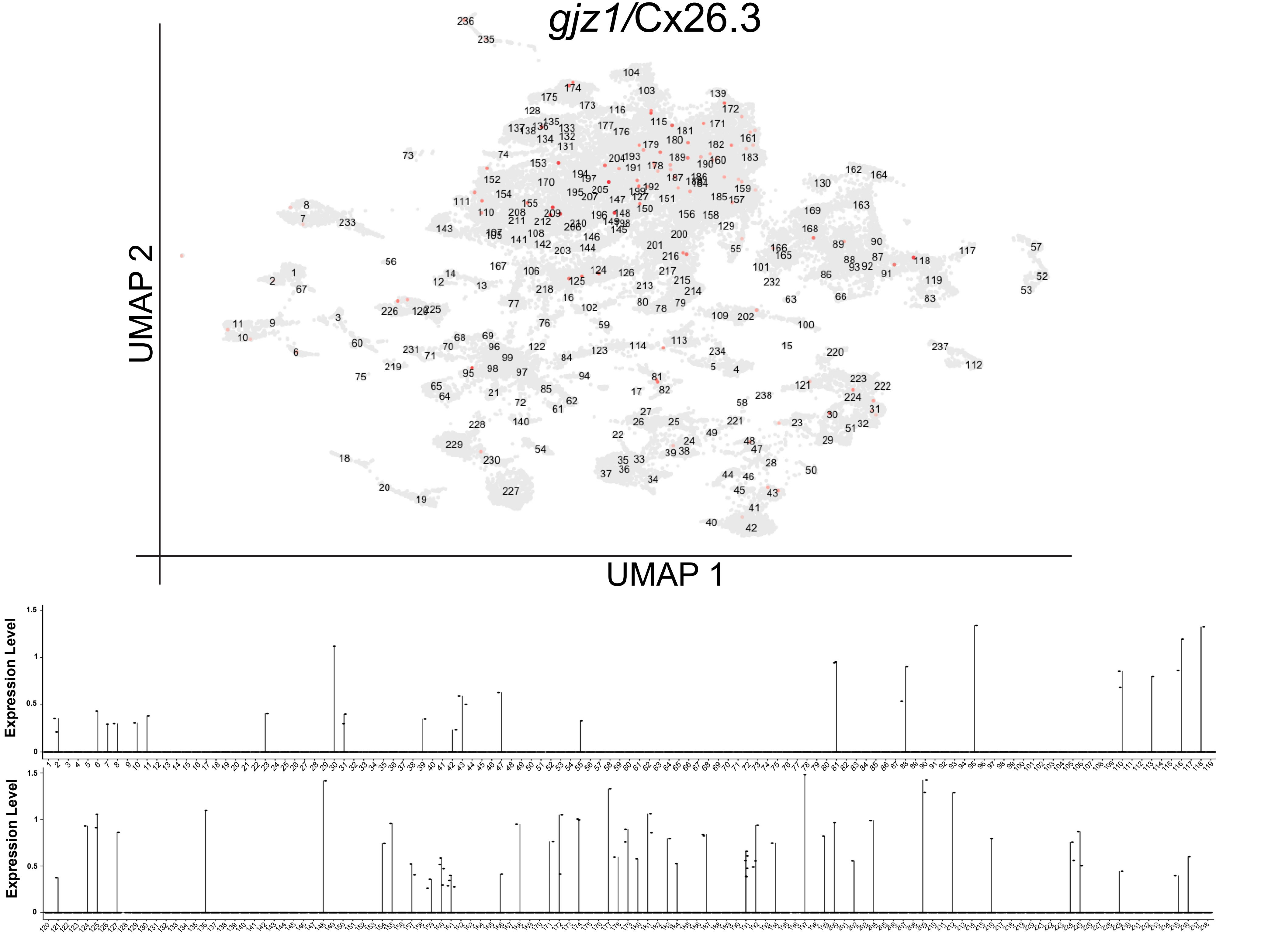

Supplemental Figure 5

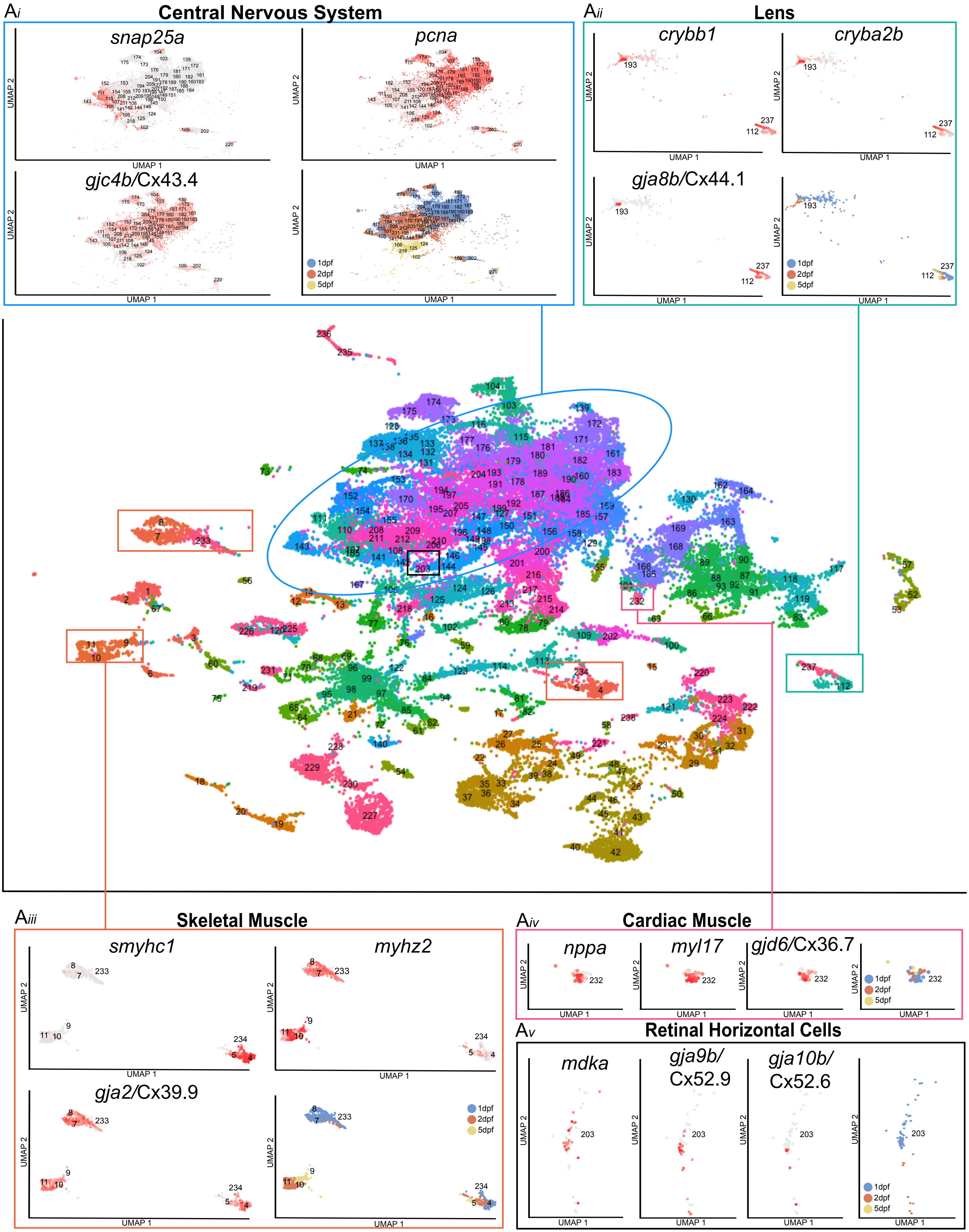

Supplemental Figure 6

A*i*

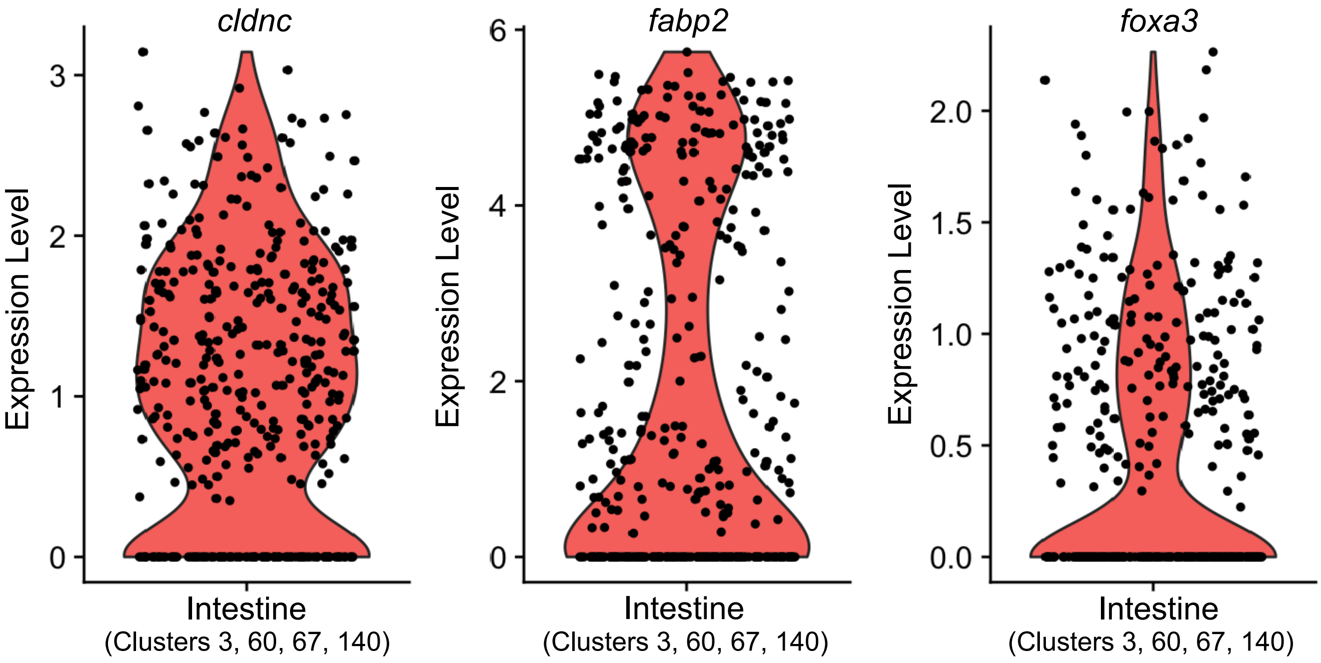

A*ii*

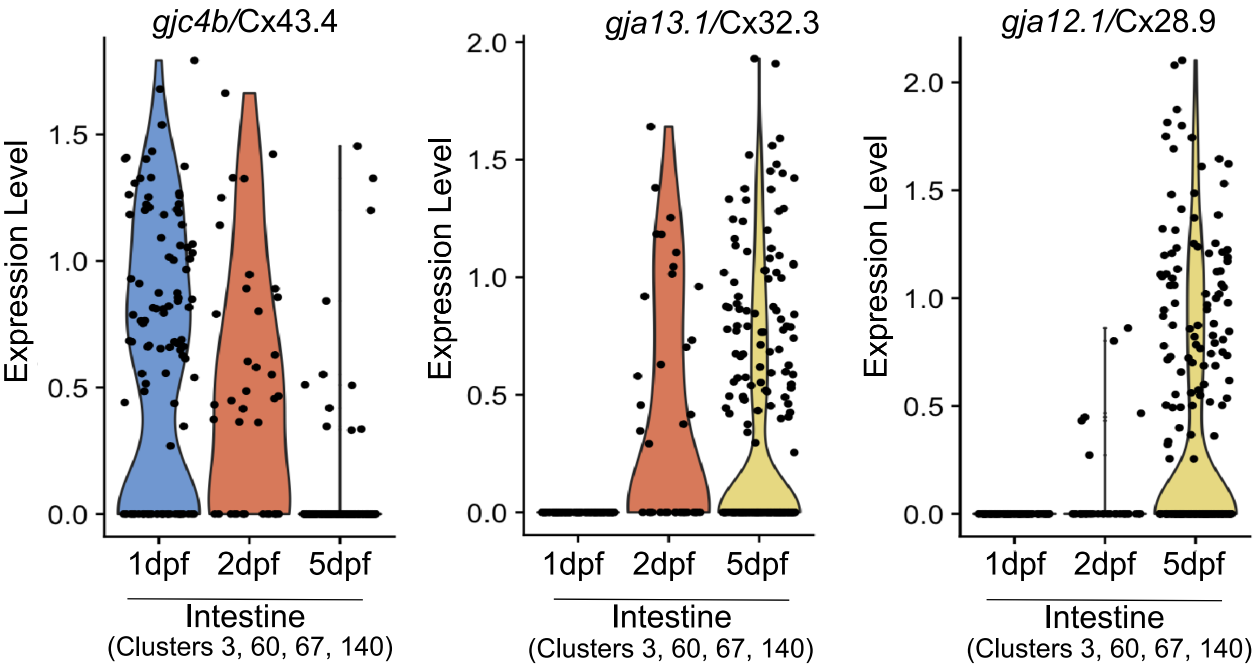

Supplemental Figure 7

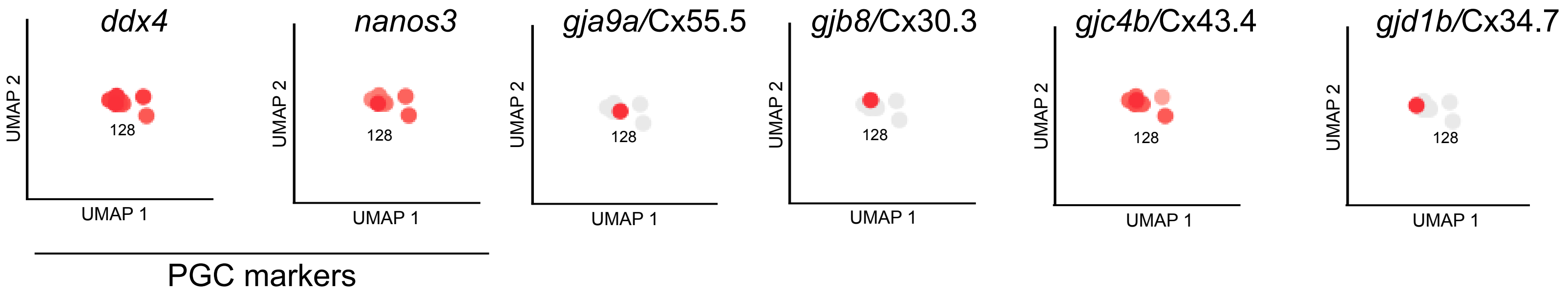

Supplemental Figure 8

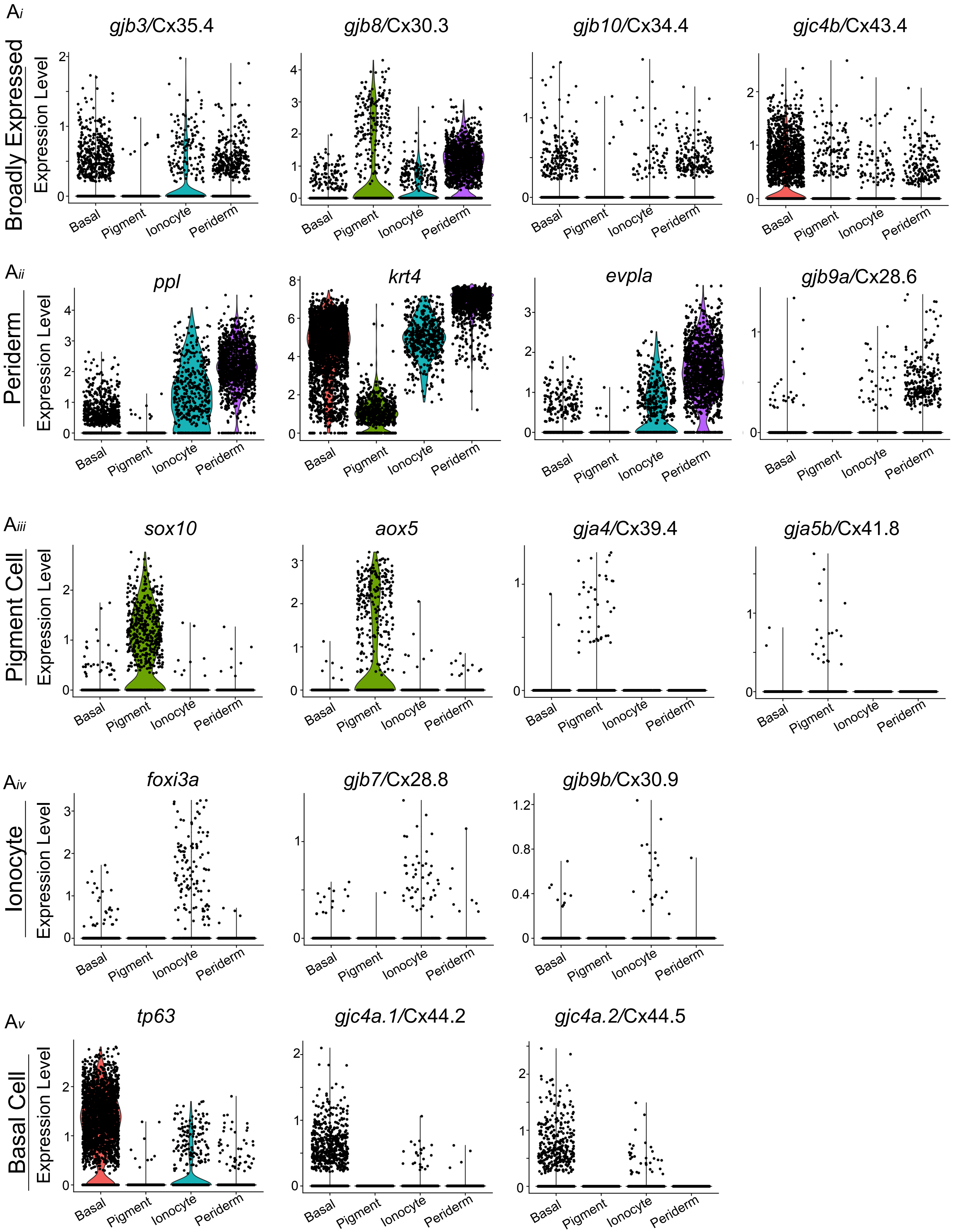

Supplement: jkac062_Supp_Figures [file jkac062_supp_figures.pdf]
