# Supplementary material for: Connexinplexity: the spatial and temporal expression of connexin genes during vertebrate organogenesis
Source: G3 (Bethesda). 2022 Mar 24;12(5):jkac062. doi: 10.1093/g3journal/jkac062 (PMC9073686; doi:10.1093/g3journal/jkac062)
Supplement: jkac062_Supplemental_Figure_Legend [file jkac062_supplemental_figure_legend.docx]

**Supplemental Figure and Tables Titles/Legends**

Supplemental Figure 1: Phylogeny of human (*hs*) and zebrafish (*dr*) Connexin proteins.

Supplemental Figure 2: Protein similarities and phylogeny of the novel *gjz1*/Cx26.3. (A) Zebrafish (*dr.*) Gjz1/Cx26.3 aligned with the most similar human (*hs*.) Connexin protein, GJB3. The predicted intercellular portions (coral), transmembrane domains (green) and extracellular loops (blue) for GJB3 are denoted above the sequence. (B) Ensembl-generated phylogenetic tree of *gjz1*/Cx26.3 (si:rp71-1c10.10 in red). Bony fishes are highlighted in pink, while lobed-fin lineages from coelacanth to mammals are in other colors. Protacanthopterygii and Euacanthomorphacea sub-trees are collapsed for visual purposes (grey triangle). Each related gene is represented on a node and colored based on Ensembl predications, including a gene node (white box), speciation nodes (dark blue box), duplication nodes (red box), and ambiguous nodes (teal box). Genomic sequence alignment similarity is denoted on the right with black boxes (representing 66-100% alignment), moderate sequence alignment is denoted on the right with green boxes (representing 33-66% sequence alignment) and gaps in alignments are denoted in white.

Supplemental Figure 3: Gene model updates to capture *connexin* expression. (A) Aligned sequencing view showing coverage pileups for scRNA-seq (1, 2, 5 dpf) and bulk RNA-seq, as well as bulk RNA-seq reads (grey boxes). The Ensembl GTF (light green) captures few reads associated with *gjd4*/Cx46.8. Extending the gene model (dark green) in the updated GTF captures these missed transcripts. (B) Comparisons between the Farnsworth et al., 2020 dataset and the updated dataset. (B*i*) Farnsworth cluster 57 has robust expression of slow muscle marker *smyhc2*, but poor expression of *gjd4*/Cx46.8. In the updated dataset, the related cluster 4 has similar expression of slow muscle marker *smyhc2* but a significant increase of *gjd4*/Cx46.8 representation (right). (B*ii*) Farnsworth cluster 205 has robust expression of cardiac muscle marker *myl17* and *gjd6/*Cx36*.7*. The corresponding cluster in the updated dataset did not significantly alter either of these expression patterns (right). (B*iii*) Farnsworth cluster 173 has low expression of Schwann Cell marker, *mbpa*, as well as *gjb1a/*Cx27.5*.* The corresponding cluster in the updated dataset captures robust expression for both *mbpa* and *gjb1a/*Cx27.5*.*

Supplemental Figure 4: *connexin* expression throughout the atlas. Note that this figure extends across 41 pages, one for each *connexin* gene, labeled A-OO. Expression of each *connexin* is plotted on the scRNAseq atlas and visualized through color intensity on UMAP plots and by violin plots for each gene and cluster.

Supplemental Figure 5: Tissue and cell type markers with corresponding *connexin* expression. (A*i*) Central nervous system clusters express *snap25a* and *pcna*, and *gjc4b*/Cx43.4. (A*ii*) Lens clusters (193, 112 and 237) express lens markers *crybb1* and *crybab2*, and *gja8b/*Cx44.1. (A*iii*) Skeletal muscle clusters express slow muscle marker *smyhc1* (4, 5, 234), fast muscle marker *myhz2* (7, 8, 9, 10, 11, 233), and *gja2*/Cx39.9. (A*iv*) Cardiac muscle cluster 233 expresses markers *nppa* and *myl17*, and *gjd6*/Cx36.7. (A*v*) Retinal horizonal neurons, cluster 203, express marker *mdka* and *gja9b*/Cx52.9 and *gja10b*/Cx52.6.

Supplemental Figure 6: Temporal expression patterns of *connexins* within the intestine. (A*i*) Intestinal clusters (3, 60, 67, 140) selected for expression of canonical markers *cldnc*, *fabp2*, and *foxa3*. (A*ii*) These clusters display an evolution of *connexin* expression at different developmental time points. *gjc4b*/Cx43.4 (left) is expressed at 1 and 2 dpf, *gja13*.1/Cx23.3 is expressed at 2 and 5 dpf, and *gja12*.1/Cx28.9 is expressed at 5 dpf.

Supplemental Figure 7: Primordial germ cells express several *connexins*. Putative primordial germ cell (PGC) cluster (128) expressing PGC markers like *ddx4* and *nanos3*, in addition to *connexins* including *gja9a/*Cx55.5, *gjb8/*Cx30.3, *gjc4b/*Cx43.4, and *gjd1b/*Cx34.7.

Supplemental Figure 8: *connexin* expression in the integument. Clusters classified into cell types are grouped and labelled accordingly. (A*i*) *gjb3*/Cx35.4, *gjb8*/Cx30.3, *gjb10*/Cx34.4, and *gjc4b*/Cx43.4 are all expressed broadly through the integument clusters. (A*ii*) *ppl*, *krt4,* and *evpla* are expressed highly in periderm clusters, with *gjb9a*/Cx28.6 being primarily expressed in periderm clusters. (A*iii*) *sox10* and *aox5* expression identifying pigment cell clusters with *gja4*/Cx39.4 and *gja5b*/Cx41.8. (A*iv*) *foxi3a* expression identifying ionocyte clusters with *gjb7*/Cx28.8 and *gjb9b*/Cx30.9. (A*v*) *tp63* expression identifying basal cell clusters with *gjc4a*.1/Cx44.2 and *gjc4a*.2/Cx44.5.

Supplemental Table 1: The zebrafish *connexin* family.

Supplemental Table 2: Transferring Farnsworth et. al, 2020 cluster annotations to the updated dataset. Updated cluster number (Column A) and the corresponding Farnsworth cluster (Column B). The count of cells from the Farnsworth cluster that ended in the corresponding updated cluster (Column C), and the proportion of cells from a given Farnsworth cluster that contribute to the updated cluster (Column D, E). Total cell counts are colored in blue and bolded. All previous Farnsworth annotations were transferred over (columns H-AE), and update cluster markers are included in orange. The most significant Farnsworth cluster contributor is denoted in black font.

Supplemental Table 3: List of differentially expressed genes for each cluster. (Sheet1) For each cluster (Column A), annotations at germ layer, tissue, cell type, and subtype (Columns B – E) level are listed. For each cluster, the top 16 most differentially expressed genes are listed (Column F - U). (Sheet 2) All differentially expressed genes for each updated cluster, generated using the FindAllMarkers command of Seurat, using the Wilcoxon rank sum test. Pct.1 (Column D) and Pct.2 (Column E) reflect the fraction of cells expressing each marker gene (Boolean) within each cluster and for all other cells, respectively.

Supplemental Table 4: The proportion of cells within each cluster that express each *connexin.*

Supplemental Table 5: All reagents used for fluorescent RNA in-situ and other immunohistochemistry in this study*.*
